# Abstracts from the ISSAID 2021 Periodic Congress: Virtual. May - Dec 2021

**DOI:** 10.1186/s12969-021-00659-2

**Published:** 2022-01-10

## Oral presentations

### O01 The possible effect of miR-30e-3p which targets IL-1Β in the pathogenesis of systemic autoinflammatory diseases

#### T. H. Akbaba^1^, Y. Akkaya-Ulum^1^, E. Batu^2^, E. Sönmez^2^, M. V. Gijn^3^, D. Foell^4^, M. Gattorno^5^, S. Ozen^2^, B. Balci-Peynircioglu^1^

##### ^1^Medical Biology; ^2^Pediatric Rheumatology Unit, Hacettepe University, Ankara, Turkey; ^3^University Medical Center, Utrecht, Netherlands; ^4^Department for Pediatric Rheumatology & Immunology, Muenster, Germany; ^5^Giannina Gaslini Institute (IRCCS), Genoa, Italy

###### **Correspondence:** T. H. Akbaba

**Introduction:** Systemic Auto Inflammatory Diseases(SAID) is a group of rare hereditary fever syndromes. Phenotypic heterogeneity among SAID patients is quite common, and epigenetic factors can be a cause of these variable clinical profiles. MiRNAs take an active role in the regulation of inflammation.

**Objectives:** This study aims to investigate the potential impact of miRNAs in autoinflammation.

**Methods:** Expression levels of miRNAs in blood samples of 6 severe FMF and 7 mild FMF, 6 other rare SAIDs, and healthy controls were analyzed by miRNA array, bioinformatics tools and pathway analyzes related to inflammatory pathways. The candidate miRNAs were functionally studied for expression levels of inflammatory genes, caspase I activation, apoptosis, cell migration assays in SW982 cells. Then, 3'UTR luciferase activity experiments were carried out to determine the target gene. Then, target gene expression studies were performed at both RNA and protein levels. Also, miR-30e-3p was analyzed in a group (total of 44 patients) of European patients (Germany, Italy, and the Netherland).

**Results:** The expression levels of miRNAs by miRNA array were confirmed by qRT-PCR. miR-30e-3p was significantly reduced among patient groups. After pre-miR transfection of miR-30e-3p; expression levels of inflammatory genes (IL1β, IL18, TNFα, TGFβ) and apoptosis rates decreased, caspase I activation and cell migration rate decreased significantly (p <0.05). As a result of functional analysis, target gene studies were performed with miR-30e, which has an anti-inflammatory effect in all experimental systems. It has been shown that miR-30e is the direct target of the IL-1β gene. Expression of the IL-1β gene at RNA and protein levels decreased in pre-miR-30e-3p transfected cells. Also, miR-30e-3p was decreased in a group of European SAID patients (Germany, Italy, and the Netherland).

**Conclusion:** The results showed that miR-30e-3p has an anti-inflammatory effect by regulating IL-1β expression, which is a key protein in inflammatory pathways.

**Acknowledgments**

This project has been funded by the ERARE3 project (INSAID,003037603) and The Technical and Scientific Research Council of Turkey, 315S096.

**Disclosure of Interest**

None Declared

### O02 Number of episodes can be used as a disease activity measure in familial mediterranean fever

#### D. Piskin^1^, Z. Arici^2^, D. Konukbay^3^, M. Romano^4^, S. Ozen^5^, K. Speechley^1^, E. Demirkaya^4^

##### ^1^University of Western Ontario, London, Canada; ^2^Pediatric Rheumatology, Sanliurfa Research and Training Hospital, Sanliurfa; ^3^University of Health Sciences, Ankara, Turkey; ^4^Pediatric Rheumatology, University of Western Ontario, London, Canada; ^5^Pediatric Rheumatology, Hacettepe University, Ankara, Turkey

###### **Correspondence:** D. Piskin

**Introduction:** Monitoring disease activity in Familial Mediterranean Fever (FMF) is essential to define disease effects on the general health and quality of life (QoL) of patients, to determine treatment response, optimize disease follow-up and prevent complications. The importance of monitoring disease activity and doing regularly are highlighted in the international recommendations for the management of autoinflammatory diseases. It is necessary to assess disease activity easily in research and clinical practice.

**Objectives:** To assess the validity of number of episodes as a feasible stand-alone measure of disease activity in these patients, we developed *a priori* predictions of expected associations that number of episodes in a year would have with particular PROMS based on the evidence in the literature regarding observed associations with level of disease activity in patients with FMF. Specifically, we predicted the following associations in children with FMF: functional status, quality of life, level of depressive symptoms and perceived level of pain would be negatively associated with number of episodes in the past year.

**Methods:** In this cross-sectional study, patients were recruited from the seven tertiary hospitals in Turkey. Demographic data, main clinical symptoms of the episodes, treatment modalities, and genetic mutations were recorded. The patients were grouped as: no episodes (Group 1), 1-4 episodes (Group 2), more than 4 episodes (Group 3) according to number of episodes in the past 12 months. The four-item Morisky Medication Adherence Scale (MMAS-4), Pediatric Quality Life Inventory (PedsQL), Children's Depression Inventory (CDI) and Wong-Baker FACES pain rating scale (FACES) scores were compared between groups.

**Results:** A total of 239 patients (male 44.4%, female 55.6%) were included. There were 74 patients (31%) in Group 1, 99 patients (41.4%) in Group 2 and 66 patients (27.6%) in Group 3. Age at diagnosis, gender, consanguinity, family history and history of amyloidosis were not different among the groups (p>0.05). The main clinical symptoms were similar among the groups and also the groups were compared according to the each item on the AIDAI symptom scale and there were no statistically significant differences among groups (p>0.05). Most of the patients (232 of 239 patients, 97.1%) were treated with colchicine. Groups were similar in terms of M694V, and V726A allele frequency (p=0.843, p=0.46). For parent and child PedsQL scale scores, patients in no episode group (Group 1) had higher scores that means better HRQoL than Group 2, and Group 2 had higher scores (better HRQoL) than Group 3. Both parent and child MMAS scores were not different among the groups. In Group 3, patients have higher parent CDI scores than no episode (Group 1) group (p<0.001). Child CDI scores were significantly lower in Group 1 than Group 2 (p=0.01), and in Group 2 than Group 3 (p=0.03). Both parent and child FACES scores were significantly lower in no episode group than Group 2, and patients in Group 2 lower than Group 3.

**Conclusion:** In a homogeneous patient population in terms of demographic features, mutation types, clinical symptoms, and treatment, increasing number of episodes was associated with worst PedsQL, CDI, and FACES scores. We conclude that number of episodes is the key element of disease activity in patients with FMF and can be used as a single measure to assess disease activity.

**Disclosure of Interest**

None Declared

### O03 Long-term cardiac outcome in patients with multisystem inflammatory syndrome in children (MIS-C)

#### S. Della Paolera^1^, F. Zunica^2^, D. Montin^3^, G. Zuccotti^4^, F. La Torre^5^, S. Mannarino^6^, M. Fabi^7^, A. Taddio^1,8^, M. Cattalini^2^

##### ^1^Institute for Maternal and Child Health, IRCCS “Burlo Garofolo”, Trieste; ^2^Pediatrics Clinic, ASST Spedali Civili di Brescia, University of Brescia , Brescia; ^3^Department of Pediatrics and Public Health, University of Turin, Turin; ^4^Department of Pediatrics, University of Milan, Children’s Hospital V Buzzi, Milan; ^5^Pediatric Rheumatology Center, Pediatric Unit, “Giovanni XXIII” Pediatric Hospital, Bari; ^6^Division of Cardiology, Children's Hospital V Buzzi, ASST FBF Sacco, Milan; ^7^Department of Pediatrics, University of Bologna, IRCCS Sant’Orsola-Malpighi Hospital, Bologna; ^8^University of Trieste, Trieste, Italy

###### **Correspondence:** A. Taddio

**Introduction:** Multisystem Inflammatory Syndrome in children (MIS-C) was initially described during the first phase of COVID-19 pandemic as a severe clinical condition with systemic inflammation and multi-organ involvement. Many patients show features of Kawasaki Disease. Cardiac involvement, from myocarditis to coronary abnormalities, is a key feature of the syndrome, but there are still little data concerning the long-term outcome in these patients.

**Objectives:** The aim of our study was to evaluate long-term cardiac outcome in patients diagnosed with MIS-C during the first phase of COVID-19 pandemic in Italy.

**Methods:** We previously published the results of the Italian multicenter survey of MIS-C, launched by the Rheumatology Study Group of Italian Pediatric Society during the first wave of COVID-19 pandemic. For each patient who received MIS-C diagnosis, we collected demographic, clinical, laboratory data, imaging findings, and treatment information in an online anonymized database (RedCAP). Data collection included all the admission period and all follow/up visits. For the purpose of this study, we analyzed all patients with at least 3months of follow/up mainly focusing on heart involvement.

**Results:** Fifty-three patients who received MIS-C diagnosis between February 1st and May 31st 2020 were included in our study. The median age at diagnosis was 7 years (IQR 4,5-11). Forty-one patients showed cardiac involvement during the course of the disease. Treatment with IVIG was reported in 66% of patients at diagnosis and glucocorticoids in 56,6%. Four patients received treatment with IL-1 receptor antagonist (anakinra) and one with hydroxychloroquine. Use of vasoactive agents was reported in 20,8% of patients. No case of death was reported in our population.

Data on cardiac outcome were available for 33 patients after a median time of follow-up of 6 months (IQR 7.2- 4.08). Twenty-eight out of 33 patients presented a cardiac involvement during hospitalization for MIS-C: 17 had myocarditis, 5 had pericarditis, 3 coronary artery abnormalities, 8 heart failure, 9 valvular insufficiency, 11 shock or hypotension. For each patient cardiac outcome was assessed by heart ultrasonography. At the end of our follow-up period only four patients still had heart abnormalities: all of them presented mild valvular insufficiency and 2 patients still had ultrasonographic signs of hypokinesia. None of them was on medication.

**Conclusion:** MIS-C is an emerging inflammatory condition that spreads among the pediatric population in parallel to SARS-CoV2 pandemic. The disease is frequently complicated by cardiological involvement but, differently to Kawasaki disease, myocarditis and shock are the most common complications. As we reported in our previous study, short-term outcome is usually good in children with MIS- C and heart involvement. With this study we also provide a long-term cardiac follow-up and we showed that only a minority of patients with previous cardiac involvement presented minor heart abnormalities. Furthermore, no patients developed new heart disease during follow-up.

**Acknowledgments**

Francesca Biscaro: UOC Pediatria, Ospedale Ca’ Foncello, Treviso. Andrea Campana: Bambino Gesù Children’s Hospital, Rome, Italy. Rosa Maria Dellepiane: Pediatric Intermediate Care Unit, Fondazione IRCCS Ca’ Granda Ospedale Maggiore Policlinico, Milan, Italy. Angela Mauro: Department of Paediatrics, Emergency Department, Santobono-Pausilipon Children’s Hospital, Naples, Italy. Alesssandra Meneghel: Department of Woman’s and Child’sHealth, University of Padova, 2 Padua, Italy. Alma Olivieri: Dipartimento della donna, del bambino e di chirurgia generale e specialistica, Università della Campania, “L Vanvitelli, Napoli. Rita Sottile: Department of Paediatrics, Santobono-Pausilipon Children’s Hospital, Naples, Italy.  Sara Stucchi and Barbara Teruzzi : Maternal and Child Health, Division of Paediatrics, ASST Grande Ospedale Metropolitano Niguarda, Milano, Italy.   Tatiana Utytatnikova: Dipartimento Materno-Infantile, Pediatria, ASST Bergamo-EST, Seriate, Bergamo. Gianluca Vergine: UOC Pediatria, Ospedale degli Infermi di Rimini, Rimini, Italy.

**Disclosure of Interest**

None Declared

### O04 SARS-CoV2 ORF3a protein drives inflammation in COVID-19 through NLRP3 inflammasome activation

#### A. Bertoni^1^, F. Penco^1^, H. Mollica^1,2^, P. Bocca^1^, I. Prigione^1^, A. Corcione^1^, D. Cangelosi^1^, A. Amaro^3^, N. Paladino^1^, E. Pontali^4^, M. Feasi^4^, S. Signa^1,2^, M. Bustaffa^1^, R. Caorsi^1^, R. De Palma^5,6^, U. Pfeffer^3^, P. Uva^1,7^, A. Rubartelli^1^, M. Gattorno^1^, S. Volpi^1,2^ on behalf of UOSD Centro per le Malattie Autoinfiammatorie e Immunodeficienze

##### ^1^IRCCS Istituto Giannina Gaslini; ^2^DINOGMI, University of Genova; ^3^IRCCS Ospedale Policlinico San Martino; ^4^Ente Ospedaliero Ospedale Galliera; ^5^IRCCS IST-Ospedale San Martino; ^6^Internal Medicine, University of Genova; ^7^Italian Institute of Technology, Genova, Italy

###### **Correspondence:** S. Volpi

**Introduction:** COVID-19 severe pneumonia has been associated to systemic inflammation and elevation of blood parameters and reminiscent of cytokine storm syndrome. Stimulation of PBMC from patients with severe COVID-19 have shown a high secretion of IL-1β, a pivotal cytokine driving inflammatory phenotypes, which maturation and secretion is regulated by NLRP3 inflammasome. Steroidal anti-inflammatory therapies have shown efficacy in reducing mortality in critically ill patients, however the mechanisms by which SARS-CoV2 virus triggers such an extensive inflammation remain unexplained.

**Objectives:** The overall objective of this study was to investigate if SARS-CoV2 drives inflammation in COVID-19 patients through NLRP3 inflammasome activation and IL-1β secretion.

**Methods:** Samples from SARS-CoV2 infected patients, were collected at day 0 and at 3 and 7 following treatment with anakinra. Fresh monocytes, purified through adherence, were cultured for 3, 6, 18 h in the presence or absence of LPS (100 ng/ml) and MCC950 (10μM). Release of IL-1β, IL-1Ra, IL-6, TNF-α, IL-18 was quantified by ELISA kit. Relative gene expression analysis of ORF3a gene was performed by RT-qPCR. THP-1 cells were transfected with a plasmid containing ORF3a sequence by nucleofection. NLRP3 inflammasome and ASC speck formation were detected by confocal microscopy and/or by FACS analysis.

**Results:** In the present study we show that circulating monocytes from COVID-19 patients display ASC specks, index of NLRP3 activation, and spontaneously secrete IL-1β in vitro. This spontaneous activation reverts following patient’s treatment with the IL-1 receptor antagonist anakinra. Transfection of a monocytic cell line with cDNA coding for the ORF3a SARS-CoV2 protein, resulted in NLRP3-dependent ASC speck formation. The involvement of ORF3a in inflammasome activation was further supported by the detection by RT-PCR of ORF3a in monocytes from COVID-19 patients.

**Conclusion:** In summary, these results provide a mechanistic explanation for the strong inflammatory manifestations associated to COVID-19 and further evidence that NLRP3 and IL-1β targeting could represent an effective strategy in this disease.

**Acknowledgments**

We thank the patients and their families for their willingness to participate in our study, the service of Immunohematology and transfusion medicine of Gaslini Institute for the collection of biological samples and the Laboratory Core Facility of Gaslini Institute for support for confocal analysis. SV received financial support from AMRI, “associazione malattie reumatiche infantili”. IRCCS G Gaslini is members of the European Reference Network for Rare Immunodeficiency, Autoinflammatory and Autoimmune Diseases -Project ID No 739543. This work was developed within the framework of the DINOGMI Department of Excellence of MIUR 2018-2022

**Disclosure of Interest**

None Declared

### O05 Defining clinical and genetic hallmarks of VEXAS syndrome

#### D. Ospina Cardona^1^, L. L. Wilson^1^, M. A. Ferrada^2^, A. K. Ombrello^1^, P. C. Grayson^2^, I. Aksentijevich^1^, D. B. Beck^1^, D. L. Kastner^1^

##### ^1^National Human Genome Research Institute; ^2^National Institute of Arthritis and Musculoskeletal and Skin Diseases, Bethesda, Maryland, United States

###### **Correspondence:** D. Ospina Cardona

**Introduction:** VEXAS (Vacuoles, E1 enzyme, X-linked, Autoinflammatory, Somatic) syndrome is a late-onset, autoinflammatory disorder discovered last year through shared genetic variants as opposed to shared clinical descriptions. VEXAS is caused by hematopoietic, somatic mutations in the X-chromosomal *UBA1* (ubiquitin activating enzyme 1) gene encoding the primary E1 enzyme of the essential ubiquitylation pathway. VEXAS patients share variants in the *UBA1* gene while displaying broad clinical heterogeneity in disease severity and onset.

**Objectives:** We aimed to further elucidate the clinical features that are predictive of VEXAS syndrome through analyzing new patient phenotypes, to allow for earlier detection and therapeutic intervention. We also sought to determine the variant distribution amongst VEXAS patients and compare with disease course to establish if a specific mutation can be a biomarker of disease severity.

**Methods:** To identify key phenotypic presentations of VEXAS that warrant genetic testing we compared the clinical characteristics of our first reported 25 patients and our newly identified additional 43 patients. We performed Sanger sequencing and digital droplet PCR (ddPCR) to detect and quantify mutation levels in our referred patients.

**Results:** After the initially reported clinical manifestations of VEXAS (25 patients last year), 80 patients have been referred to our clinic based on clinical similarities to our first report. 43 new patients have been identified with somatic mutations in *UBA1*, making our current diagnostic rate 54%. The clinical characteristics of newly diagnosed patients are highly consistent with the reported manifestations of the initial VEXAS cohort with some expanded phenotypes including renal, neurologic and hematologic manifestations. We determined that patients with history of chondritis and MDS have a higher positivity rate than those with general systemic inflammation. Variant distribution in our expanded cohort is consistent with our initial report, with threonine being the most common p.Met41 variant (56%) followed by valine and leucine (26% and 18% respectively). 35% of patients with the valine variant are deceased as compared to 16% for other mutations, indicating that p.Met41Val may confer a more severe phenotype.

**Conclusion:** Our 54% positivity rate indicates that the initially reported clinical manifestations are effectively identifying additional VEXAS patients. Through analyzing the symptoms of our larger cohort, we have further characterized the phenotype of this novel syndrome. Additionally, identifying the p.Met41Val variant as a marker of severe disease may offer more accurate prognosis and the possibility for earlier intervention. Together, this work provides further insights into VEXAS syndrome with the goal of increasing diagnosis and improving management

**Acknowledgments**

We would like to sincerely thank all the patients and their families involved in our continued studies for their incredible contributions.

**Disclosure of Interest**

None Declared

### O06 Adult-onset autoinflammation caused by somatic mutations in UBA1: a Dutch case series of VEXAS patients

This abstract has not been included here as it has been previously published

### O07 Short-term follow-up results of children with familial mediterranean fever after cessation of canakinumab

#### B. Sözeri^1^, H. E. Sönmez^2^, S. Karadağ^3^, E. Baglan^4^, K. Öztürk^5^, M. Çakan^6^, F. Demir^1^, G. Otar Yener^7^, S. Özdel^4^, N. Ayaz^8^

##### ^1^Department of Peditarics, Division of Rheumatology, Umraniye Research and Training Hospital, Istanbul; ^2^Department of Peditarics, Division of Rheumatology, Kocaeli University, Kocaeli; ^3^Department of Peditarics, Division of Rheumatology, Sadi Konuk Research and Training Hospital, Istanbul; ^4^Department of Peditarics, Division of Rheumatology, Sami Ulus Research and Training Hospital, Ankara; ^5^Department of Peditarics, Division of Rheumatology, Istanbul Medeniyet University Göztepe Research and Training Hospital; ^6^Department of Peditarics, Division of Rheumatology, University of Health Science, Zeynep Kamil Maternity and Children’s Diseases Research and Training Hospital, Istanbul; ^7^Department of Peditarics, Division of Rheumatology, Şanlıurfa Research and Training Hospital, Şanlıurfa; ^8^Department of Peditarics, Division of Rheumatology, Istanbul University, Istanbul, Turkey

###### **Correspondence:** H. E. Sönmez

**Introduction:** Familial Mediterranean fever (FMF) is a systemic autoinflammatory disease manifesting with recurrent attacks of serositis accompanied by fever, usually lasting 12-72 hours. Although colchicine has dramatically improved the quality of life in majority of the patients by resolving attacks, unfortunately, it is ineffective in 5–10% of patients with FMF.

**Objectives:** The aim of this study was to investigate the efficacy and safety o canakinumab (CAN) in patients with colchicine-resistant (cr) FMF and to report our experience on cessation of CAN in crFMF patients.

**Methods:** This is an observational retrospective cohort study including crFMF patients treated with CAN for at least 6 months. Data of the patients were recorded at treatment onset, 1^st^, 3^rd^, 6^th^, 12^th^, 18^th^, and 24^th^ months.

**Results:** A total of 114 patients followed up 2736 person-months were included in the study. During the 24-month follow-up, drug interval was not changed in 44 patients. Injection intervals were extended in 58 patients within a median of 6 (3-18) months. Of these 58 patients, 4 patients had a new attack after the prolongation of dose interval. CAN was ceased in 12 patients (5 at 12^th^ month, 7 at 18^th^ month), two of whom experienced a new attack within 3 months. CAN was restarted and applied every 8 weeks. The remaining 10 patients did not report any symptom at the 24^th^ month. The median attack-free period under CAN treatment was 669 (95% confidence interval: 644-696) days (Table 1).

**Conclusion:** Cessation of CAN or extended drug intervals may be feasible in crFMF. The real life experience may provide clinicians to create standardized treatment approaches for these patients.

**Table 1 (abstract O07).** Assessment of drug response in the patients with colchicine resistant familial Mediterranean fever

	Baseline (n=114)	1^**st**^ month (n=114)	3^**rd**^ month (n=114)	6^**th**^ month (n=114)	12^**th**^ month (n=114)	18^**th**^ month (n=114)	24^**th**^ month (n=114)
PGA*	8 (5-10)	1 (0-10)	0 (0-5)	0 (0-5)	0 (0-3)	0 (0-5)	0 (0-3)
CRP*	16.2 (11-31)	0.65 (0.02-58.6)	0.2 (0-15.8)	0.7 (0-68)	0.5 (0-24.9)	0.3 (0-41)	0.3 (0.16.1)
SAA*	89 (2.9-1870)	3.9 (0-17)	4 (0-23)	3.3 (0-70)	3.6 (0-152)	3 (0-42)	3 (0-24)
**Dose interval of CAN (weeks)**	4 (4-8) 4 weeks in 94 8 weeks in 20	4 (4-8) 4 weeks in 94 8 weeks in 20	4 (4-8) 4 weeks in 94 8 weeks in 20	6 (4-8) 4 weeks in 66 6 weeks in 21 8 weeks in 27	6 (4-8) 4 weeks in 50 6 weeks in 21 8 weeks in 41 12 weeks in 2	8 (4-12) 4 weeks in 45 6 weeks in 21 8 weeks in 40 12 weeks in 3 No drug in 5	8 (4-12) 4 weeks in 39 6 weeks in 25 8 weeks in 38 No drug in 12
**Dosage of CAN (mg/kg)**	2-4 mg/kg	2-4 mg/kg	2-4 mg/kg	2-4 mg/kg	2-4 mg/kg	2-4 mg/kg	2-4 mg/kg
**Number of patients experienced attacks**	NA	0	2	1	5	4	2
**Number of patients requiring higher dose or shortening drug interval**	NA	0	2 (recurrent attacks)	0	2 (recurrent attacks)	4 (recurrent attacks) (shortened from 8 to 6 weeks	2 (recurrent attacks, CAN was restarted)
**Number of patients whose prolonged drug interval**	NA	0	7 patients (prolonged from 4 to 8 weeks) 21 patients (prolonged from 4 to 6 weeks)	16 patients (prolonged from 4 to 8 weeks) 2 patients (prolonged from 8 to 12 weeks)	5 patients (prolonged from 4 to 8 weeks) 1 patient (prolonged from 8 to 12 weeks)	6 patients (prolonged from 4 to 8 weeks)	NA
**Number of patients discontinued CAN**	0	0	0	0	5 patients	7 patients	0
**Number of infections**	NA	1 (pneumonia)	1 (upper respiratory tract infection)	0	0	0	2 (upper respiratory tract infection)
**Number of adverse event**	NA	0	0	0	0	0	0

**Acknowledgments**

None

**Disclosure of Interest**

None Declared

### O08 Traditional laboratory parameters and new biomarkers in Macrophage Activation Syndrome (MAS) and Secondary Hemophagocytic Lymphohistiocytosis (SHLH)

#### A. De Matteis^1^, D. Pires Marafon^1^, I. Caiello^1^, M. Pardeo^1^, G. Marucci^1^, E. Sacco^1^, F. Minoia^2^, F. Licciardi^3^, A. Miniaci^4^, I. Maccora^5^, M. C. Maggio^6^, G. Prencipe^1^, F. De Benedetti^1^, C. Bracaglia^1^

##### ^1^Division of Rheumatology, IRCCS Ospedale Pediatrico Bambino Gesù, Roma; ^2^Pediatric Rheumatology, Fondazione IRCCS Ca’ Grande Ospedale Maggiore Policlinico, Milano; ^3^Department of Pediatrics and Infectious Diseases, School of Medicine, University of Turin, Regina Margherita Children’s Hospital, Torino; ^4^Department of Pediatrics, University of Bologna, S. Orsola-Malpighi Hospital, Bologna; ^5^Pediatric Rheumatology Unit, Meyer Children's University Hospital, Firenze; ^6^University Department Pro.Sa.M.I. “G. D’Alessandro”, University of Palermo, Palermo, Italy

###### **Correspondence:** A. De Matteis

**Introduction:** Macrophage Activation Syndrome (MAS) and Secondary Hemophagocytic Lymphohistiocytosis (sHLH) are hyperinflammatory conditions caused by a cytokine storm, in which IFNγ plays a pivotal role. Prompt recognition and early treatment are essential to improve the high disease mortality.

**Methods:** Routine laboratory parameters of disease activity and severity were collected from a cohort of 103 patients, 41 sHLH, 40 MAS in the context of sJIA, and 22 sJIA without MAS, from 6 Italian centers. The samples were collected at three different time points: active disease (T0), 7-10 days from starting therapy (T1) and in clinical inactive disease on medication (from 1 to 3 months from onset) (T2). Serum levels of the IFN-γ related biomarkers (CXCL9, CXCL10, Neopterin) and IL-18 were measured at each time points by ELISA.

**Results:** 367 samples were collected from the three groups of patients in the three different time points. Thirty-eight patients with sHLH (92.7%) met the HLH-2004 diagnostic criteria while 34 patients with MAS (85.0%) met the 2016 classification criteria for MAS.

Fever was present in the majority of patients (96%), while hepatomegaly and/or splenomegaly were observed more frequently in MAS and sHLH patients (76.9% and 82.9% respectively) compared to sJIA (36.8%).

Laboratory characteristics at T0 are detailed in table 1. Using the 2016 classification criteria for MAS, in our cohort we can confirm that platelet count is a specific parameter, only 4 patients with sJIA had a value <181x10^9^/liter; while ferritin is a sensitive parameter, 94.2% of patients with MAS had ferritin >684 mg/ml. Moreover, we have found that lactate dehydrogenase (LDH) values were statistically higher in MAS and sHLH groups compared to sJIA. ROC curve of LDH values in MAS group showed a statistically significant area under the curve (AUC= 0.78, p-value <0.0001). A cut-off of 681 U/L had a sensitivity of 72.6% and a specificity of 69.2%.

CXCL9, CXCL10, neopterin and IL-18 values in T0 were significantly higher in MAS and sHLH patients compared to sJIA. Furthermore IL-18 in MAS was significantly high than in sHLH group (p<0.0001). The ROC curves performed for each biomarker showed a statistically significant AUCs (p<0.01), except for IL-18 in sHLH group. We have identified a cut off value for each biomarker in MAS (CXCL9 900 pg/ml, CXCL10 280 pg/ml, Neopterin 6.0 ng/ml, IL-18 78,000 pg/ml) and sHLH (CXCL9 1,900 pg/ml, CXCL10 270 pg/ml, Neopterin 8.0 ng/ml). CXCL9, CXCL10, neopterin and IL-18 levels lowered progressively at T1 with a normalization in T2. CXCL9 decreased faster compared to neopterin, similarly to the decrease of routine laboratory parameters.

**Conclusion:** Our results confirm that platelet count and ferritin are two relevant laboratory parameters, with high specificity and sensitivity, respectively, to diagnose MAS in the context of sJIA. Even if LDH is not included in 2016 classification criteria for MAS in sJIA, we have found that this parameter could help to discriminate MAS in sJIA, in addition to the others. Moreover, our results confirm that the IFN-γ related biomarkers and IL-18 are significantly high in patients with MAS and sHLH and might be useful for diagnosis in addition to the traditional laboratory parameters. As already reported, IL-18 could be also useful to distinguish sHLH from MAS. Moreover, these biomarkers seem to be helpful to monitor clinical evolution and treatment response.

**Disclosure of Interest**

A. De Matteis: None Declared, D. Pires Marafon: None Declared, I. Caiello: None Declared, M. Pardeo: None Declared, G. Marucci: None Declared, E. Sacco: None Declared, F. Minoia: None Declared, F. Licciardi: None Declared, A. Miniaci: None Declared, I. Maccora: None Declared, M. C. Maggio: None Declared, G. Prencipe: None Declared, F. De Benedetti Consultant for: Abbvie, SOBI, Novimmune, Novartis, Roche, Pfizer, Employee of: SOBI, C. Bracaglia Consultant for: SOBI and Novartis

**Table 1 (abstract O08).** Laboratory parameters in T0. Values are shown as median (IQR); p-value: Mann-Whitney U test

	MAS (N=52)	sHLH (N=48)	sJIA (N=40)	MAS vs sHLH	MAS vs sJIA	sHLH vs sJIA
Ferritin (ng/ml)	4,593 (1,770-10,842)	4,098 (2,075-16,867)	728 (335-2,786)	0.72	**<0.0001**	**<0.0001**
Platelet count (x10^9/l)	200 (114-310)	97 (47-184)	449 (275-559)	**0.0003**	**<0.0001**	**<0.0001**
AST (U/L)	86 (51-149)	150 (51-340)	33 (23-46)	0.07	**<0.0001**	**<0.0001**
Triglycerides (mg/dl)	188 (148-286)	226 (166-381)	108 (78-140)	0.11	**<0.0001**	**<0.0001**
Fibrinogen (mg/dl)	330 (225-437)	228 (137-331)	565 (441-691)	**0.0018**	**<0.0001**	**<0.0001**
LDH (U/L)	975 (666-1,446)	1,255 (701-2,748)	599 (407-724)	0.11	**<0.0001**	**<0.0001**
CXCL9 (pg/ml)	1,728 (798-1,1507)	4,159 (1,880-10,016)	300 (300-1,989)	0.15	**0.0010**	**<0.0001**
CXCL10 (pg/ml)	905 (229-2,646)	729 (235-3,042)	150 (150-452)	0.82	**<0.0001**	**<0.0001**
Neopterin (ng/ml)	9.4 (4.9-16.0)	20.3 (9.6-35.0)	4.3 (3.0-7.4)	**0.0023**	**<0.0001**	**<0.0001**
IL-18 (pg/ml)	159,833 (50,000-250,000)	13,640 (2230-83,909)	36,764 (8,958-82,714)	**<0.0001**	**<0.0001**	0.16

### O09 Dissecting netosis in adenosine deaminase 2 Deficiency (DADA2) patients

#### F. Schena^1^, S. Signa^2^, G. Del Zotto^3^, M. Bartolucci^4^, A. Petretto^4^, R. Bertelli^5^, R. Caorsi^6^, M. Gattorno^1,7^

##### ^1^Centre for Autoinflammatory Diseases and Immunodeficiencies, IRCCS Giannina Gaslini Institute; ^2^DINOGMI , University of Genoa; ^3^Core Facilities Flow Cytometry and Cell imaging Lab; ^4^Core Facilities-Clinical Proteomics and Metabolomics; ^5^Laboratory of Human Genetics; ^6^Second Pediatric Division and Centro Malattie Autoinfiammatorie e Immunodeficienze; ^7^Second Pediatric Division, IRCCS Giannina Gaslini Institute, Genoa, Italy

###### **Correspondence:** F. Schena

**Introduction:** Deficiency of Adenosine deaminase 2 is a monogenic autoinflammatory disorder presenting a broad spectrum of clinical manifestations, including vasculitis, immunodeficiency and hematologic disease. The genetic mutations in ADA2 gene have been associated to an insufficient ADA2 activity leading to reduction in deamination of adenosine to deoxyadenosine, and a consequent accumulation of extracellular adenosine. The pathogenic mechanisms investigated so far have elucidated a skewed polarization from the M2 macrophage subtype to the proinflammatory M1 subtype with an increased production of inflammatory cytokines (TNF-α, Interferon IFN). More recently a chronic neutrophil activation and a dysregulation of NETosis, as process induced by extracellular Adenosine, inducing TNFα secretion from Macrophages stimulated by NETs, has been implicated in the pathogenesis of DADA2.

**Objectives:** The aim of the project is to dissect NETosis directly in neutrophils isolated from DADA2 patients and healthy controls (HDs), and to quantify suicidal and vital NETosis induced by several stimuli (PMA, Adenosine, LPS) using a multispectral Imaging Flow Cytometry. To determine if NET epitopes can change depending from the inflammatory microenvironment and if protein composition of NETs is disease specific, we used quantitative proteomics approach to characterize NET proteins, released from neutrophils after different stimuli in DADA2 patients, HDs and nongenetic vasculitis. Moreover we investigated the mechanisms of NETs removal, quantifying DNAse in the plasma samples.

**Methods:** We analyzed and quantified NETosis by Imaging Flow Citometry (IFC): neutrophils isolated from peripheral blood were stimulated in vitro to induce NETosis and were analyzed in the ImageStreamXMark II IFC equipped with a MultiMag system. We evaluated also NETs remnants and DNAse in the plasma samples by ELISA assay. LC-MS/MS analyses were conducted on Orbitrap Fusion Tribrid mass spectrometer and NET protein quantification was carried out using Label-Free Quantification method.

**Results:** Neutrophils from DADA2 show a significant increased suicidal NETosis, identified as nuclear decondensation and Myeloperoxidase (MPO) colocalization with DNA, following PMA stimulation and we observed an increase also with LPS and Adenosine. Then we analyzed vital NETosis, identified as elongated shape of cells, nuclei polarized within the cell and not colocalized with MPO and we found an increased vital NETosis in DADA2 neutrophils. Accordingly, plasmatic levels of circulating nucleosomes (NET remnants) were elevated in patients, whereas DNAse levels were normal. We set up experimental conditions for proteomic analysis of NETs, induced by PMA, Adenosine and TNFα, testing two patients, HDs and patients with nongenetic vasculitis: in total we identified 1770 proteins among which a hundred of proteins were significantly up or down-modulated in DADA2 NETs compared to controls NETs.

**Conclusion:** Our findings demonstrate that neutrophils of DADA2 patients are prone to undergo NETosis and that inflammation mediators such as Adenosine or TNFa in this disease can modify this pathogenic process.

**Disclosure of Interest**

None Declared

### O10 Next generation sequencing based multiplex long-range PCR for routine genotyping of autoinflammatory disorders

This abstract has not been included here as it has been previously published

### O11 Gene therapy for IL-1-mediated systemic autoinflammatory diseases

#### M. Colantuoni^1,2^, R. Jofra Hernandez^1^, E. Pettinato^1^, M. Zoccolilllo^1,3^, L. Magnani^2^, L. Basso-Ricci^1^, S. Scala^1^, L. Sergi Sergi^1^, A. Kajaste-Rudnitski^1,2^, L. Naldini^1,2^, A. Aiuti^1,2,4^, A. Mortellaro^1^

##### ^1^San Raffaele Telethon Institute for Gene Therapy (SR TIGET), IRCCS San Raffaele Scientific Institute; ^2^Vita-Salute San Raffaele University; ^3^Department of Medicine and Surgery, Tor Vergata University; ^4^Pediatric Immunohematology and Bone Marrow Transplantation Unit, IRCCS San Raffaele Scientific Institute, Milan, Italy

###### **Correspondence:** M. Colantuoni

**Introduction:** Systemic autoinflammatory diseases (SAIDs) delineate a group of clinical entities caused by an anomaly of the innate immune response that includes rare periodic fever syndromes characterized by uncontrolled production of the proinflammatory cytokine interleukin-1 (IL-1). Patients suffering from these conditions experience severe and recurrent inflammation ranging from fevers to gradual hearing loss, blindness, and organ failure due to amyloid accumulation. Conventional therapy includes anakinra, the recombinant form of the IL-1 receptor antagonist (IL-1RA), but it has a short half-life, poor tissue distribution, and some patients respond poorly. Besides being a lifelong therapy, this treatment is no definitive cure for these patients.

**Objectives:** Here, we propose a gene therapy approach based on autologous hematopoietic stem cells transduced with a lentiviral vector expressing IL-1RA.

**Methods:** Mouse HSPCs were transduced either with a purified human IL-1RA or GFP (control) lentiviral vectors (LV). The transduction efficiency, IL-1RA production, clonogenic potential, and growth rate of HSPCs were analysed and compared after the transduction. Bone marrow (BM) chimeric mice were generated by transplanting transduced (IL-1RA-LV or GFP-LV) C57BL/6 Ly5.1 lineage-negative cells into congenic C57BL/6 Ly5.2 mice. Engraftment level and immune cell reconstitution were assessed by flow cytometry. IL-1RA and GFP chimeras were injected intraperitoneally with MSU crystals to induce IL-1 mediated inflammation. After 6 hours, peritoneal cells were collected, and the percentage of neutrophils (CD11b+Ly6G+ cells) was determined by flow cytometry. BM-derived mouse dendritic cells (BMDCs) and human monocyte cell line THP1 were transduced with IL-1RA LV, untransduced cells were used as control. Inflammasome mediated-cytokines production was measured by qRT-PCR and by ELISA.

**Results:** LV-mediated IL-1RA gene transfer in mouse HSPCs did not alter progenitor cells’ clonogenic capacity as IL-1RA transduced HSPCs proliferated and differentiated similarly to GFP transduced HSPCs *in vitro*. There was a direct correlation between vector-dose and IL-1RA levels in HPSC-derived supernatants. In IL-1RA chimeras, HSPC transduction with IL-1RA-LV led to robust and sustained IL-1RA levels in plasma. Supraphysiological expression of IL-1RA did not impact the engraftment and the clonogenic potential of HSPCs *in vivo*. Full immune reconstitution of lineage-committed cells was achieved in mice that received IL-1RA-transduced HSPCs. LV-derived IL-1RA production efficiently suppressed neutrophilic infiltration in a mouse model of IL-1-mediated peritonitis. Additionally, in vitro IL-1RA transduction of BMDCs and THP1 resulted in a reduced production of inflammatory cytokines compared to control cells.

**Conclusion:** We have successfully generated an LV delivering steady IL-1RA release in mouse HSPCs *in vitro* and *in vivo*. Gene transfer of IL-1RA in mouse HSPCs resulted in long-term hematopoietic reconstitution and decreased IL-1-mediated inflammation in an acute peritonitis mouse model, suggesting that this approach is safe and well tolerated. On-going studies investigate whether IL-1RA-LV HSPC gene therapy transduction suppresses the exaggerated IL-1 activity in cells from patients and a mouse model recapitulating SAIDs phenotype.

**Disclosure of Interest**

None Declared

### O12 Application of systems biology-based in silico tools to optimize treatment strategy in Still’s disease

This abstract has not been included here as it has been previously published

### O13 Disease flares in CANDLE/PRAAS with dose reductions of baricitinib

This abstract has not been included here as it has been previously published

### O14 Systemic juvenile idiopathic arthritis associated lung disease in Europe

#### C. Bracaglia^1^ on behalf of MAS/sJIA Working Party of PReS, F. Minoia^2^, C. Kessel^3^, S. Vastert^4^, M. Pardeo^1^, A. Arduini^1^, O. Basaran^5^, N. Kiper^6^, M. Kostik^7^, M. Glerup^8^, S. Fingerhutova^9^, R. Caorsi^10^, A. Horne^11^, G. Filocamo^2^, H. Wittkowski^3^, M. Jelusic ^12^, J. Anton^13^, S. Khaldi-Plassart^14^, A. Belot^14^, P. Dolezalova^9^, A. Ravelli^15^, S. Ozen^5^, F. De Benedetti^1^ on behalf of MAS/sJIA Working Party of PReS

##### ^1^Division of Rheumatology, IRCCS Ospedale Pediatrico Bambino Gesù, Roma; ^2^Fondazione IRCCS Ca' Grande Ospedale Maggiore Policlinico, Milano, Italy; ^3^Department of Pediatric Rheumatology & Immunology, WWU Medical Center (UKM), Muenster, Germany; ^4^Pediatric Rheumatology & Immunology, University Medical Center Utrecht, Utrecht, Netherlands; ^5^Department of Pediatrics, Division of Pediatric Rheumatology, Hacettepe University; ^6^Department of Pediatrics, Division of Pediatric Pulmonology, Hacettepe University, Ankara, Turkey; ^7^Saint-Petersburg State Pediatric Medical University, Saint-Petersburg, Russian Federation; ^8^Department of Pediatrics, Aarhus University Hospital, Aarhus, Denmark; ^9^Paediatric Rheumatology and Autoinflammatory Diseases Unit, General University Hospital, Prague, Czech Republic; ^10^Department of Pediatrics and Rheumatology, IRRCS Istituto G. Gaslini, Genova, Italy; ^11^Department of Pediatric Rheumathology Karolinska University Hospital and Department of pediatrics, Karolinska Institute, Stockholm, Sweden; ^12^Department of Paediatrics, University of Zagreb School of Medicine, Zagreb, Croatia; ^13^Pediatric Rheumatology, Hospital Sant Joan de Déu, Universitat de Barcelona, Barcelona, Spain; ^14^Pediatric Nephrology, Rheumatology, Dermatology Unit, Hôpital Femme Mère Enfant, Hospices Civils de Lyon, Lyon, France; ^15^IRRCS Istituto Giannina Gaslini and Università degli Studi di Genova, Genova, Italy

###### **Correspondence:** C. Bracaglia

**Introduction:** Chronic parenchymal lung disease (LD) is a new emerging severe life-threatening complication of sJIA. The number of sJIA patients with LD is apparently increasing and interestingly it is reported more frequently in North America. Data regarding frequency and features of sJIA-LD in Europe are not available.

**Objectives:** To evaluate the burden of sJIA-LD in Europe.

**Methods:** Patients with diagnosis of sJIA with LD, including pulmonary alveolar proteinosis (PAP), interstitial lung disease (ILD) and as pulmonary arterial hypertension (PAH), followed in European paediatric rheumatology centres were identified through a survey sent to the members of the MAS/SJIA Working Party.

**Results:** Data from 28 sJIA-LD patients, diagnosed in 12 European paediatric rheumatology centres between 2005 and 2020, were collected. Twenty-seven patients were Caucasian and 1 African-American, 16 were female, the median age at sJIA onset was 6 years and LD onset occurred after a median time of 2.8 years. Sixteen patients had a chronic persistent sJIA disease course, 11 had a polycyclic course and only 1 patient had a monocyclic course; 25 (89%) had active sJIA at time of LD diagnosis. During the disease course, 22 (78%) patients developed MAS, 10 (35%) of whom had MAS at sJIA onset and 15 (54%) had full-blown MAS at time of LD diagnosis; 17 (77%) patients had >1 MAS episode. Twenty-two (78%) patients were treated with at least one IL-1 or IL-6 inhibitor before LD diagnosis: 18 with anakinra, 13 with canakinumab and 10 with tocilizumab. Eleven (39%) patients experienced drug adverse reaction to a cytokine inhibitor: 8 to tocilizumab and 3 to anakinra. Twenty-one (75%) patients developed ILD, 5 (18%) PAP and 3 (11%) PAH. 13 (46%) patients presented acute digital clubbing; 11 (39%) patients developed hypoxia and 6 (21%) developed pulmonary hypertension. A chest CT scan was performed in all patients with evidence of septal thickening and peri-bronchovascular thickening in the majority of patients (22 and 15 respectively). In 14 patients a bronchoalveolar lavage was performed and 11 underwent a lung biopsy. The histopathological pattern was alveolar proteinosis in 4 patients, endogenous lipoid pneumonia in 3, vasculitis in 1 and fibrosis in 1. Most of the patients (93%) were treated with glucocorticoids (GCs) at time of diagnosis, and 24 (86%) received IL-1 or IL-6 inhibitor after the diagnosis (18 anakinra, 12 canakinumab, 14 tocilizumab). Twelve (43%) patients required ICU admission and 4 (14%) died.

**Conclusion:** Lung involvement is an emerging life-threatening complication of sJIA in Europe, particularly in patients with a history of MAS, and a prompt recognition is crucial. New strategies are needed to reduce the risk and improve outcome of this complication.

**Disclosure of Interest**

C. Bracaglia Consultant for: SOBI, Novartis, F. Minoia Consultant for: SOBI, C. Kessel Consultant for: SOBI, Novartis, S. Vastert Consultant for: SOBI, Novartis, M. Pardeo: None Declared, A. Arduini: None Declared, O. Basaran: None Declared, N. Kiper: None Declared, M. Kostik: None Declared, M. Glerup: None Declared, S. Fingerhutova: None Declared, R. Caorsi Consultant for: Novartis, Lilly, A. Horne: None Declared, G. Filocamo Consultant for: SOBI, H. Wittkowski: None Declared, M. Jelusic : None Declared, J. Anton Grant /Research support from: Sobi, Novimmune, Novartis, abbvie, Pfizer, GSK, Roche, Amgen, Lilly, BMS, Sanofi, Consultant for: Sobi, Novimmune, Novartis, Pfizer, GSK, Speaker bureau of: Sobi, Novimmune, Novartis, GSK, Pfizer, S. Khaldi-Plassart: None Declared, A. Belot: None Declared, P. Dolezalova: None Declared, A. Ravelli Consultant for: AbbVie, Novartis, Pfizer, Angelini, Reckitt Benkiser, S. Ozen Consultant for: SOBI, Novartis, Pfizer, F. De Benedetti Employee of: SOBI, Novimmune, Novartis, Roche, Pfizer

### O15 Tocilizumab for VEXAS syndrome with relapsing polychondritis: extension study

#### Y. Kunishita, Y. Kirino, N. Tsuchida, A. Maeda, L. Hirahara, H. Nakajima

##### Department of Stem Cell and Immune Regulation, Yokohama City University Graduate School of Medicine, Yokohama, Japan

###### **Correspondence:** Y. Kunishita

**Introduction:** Recently, VEXAS syndrome, a severe autoinflammatory disease in adults caused by *UBA1* somatic mutations, has been reported. In addition to systemic inflammatory pathology, VEXAS syndrome is associated with hematological abnormalities such as myelodysplastic syndrome, making it difficult to treat in clinical practice and forcing patients to continue moderate or higher doses of steroids. Therefore, there is an unmet need to develop therapies that can reduce steroids and improve prognosis in VEXAS syndrome.

**Objectives:** To identify promising therapeutic targets and therapies for VEXAS syndrome.

**Methods:** Patients with clinically diagnosed relapsing polychondritis (RP) in our department and known *UBA1* mutations identified by Sanger sequencing (VEXAS-RP), who had been introduced to tocilizumab (TCZ), an IL-6 receptor antibody, were included in the study. Clinical information and laboratory findings were collected retrospectively from electronic medical records to validate the efficacy of TCZ. Fractional abundance of *UBA1* variant in peripheral blood was detected using dd-PCR. Serum cytokine levels before and after TCZ administration were measured using a cytometer bead array.

**Results:** Three patients with VEXAS-RP, all male, were receiving TCZ in our department. All patients were still receiving TCZ at their last visit and could continue for more than six months. After the initiation of TCZ, fever was relieved in all patients, and blood tests showed a decrease in CRP levels and an increase in albumin levels, which was thought to be the effect of blocking the IL-6 pathway to reduce systemic inflammation. As a result, all patients were able to reduce steroid use after starting TCZ. In the patient with severe anemia and thrombocytopenia (RP16 on Table 1), the frequency of receiving red blood cell transfusions decreased after TCZ administration, and his platelet counts had improved to over 100,000/μl. However, macrocytic changes in erythrocytes were still observed in all patients after TCZ administration, suggesting that the effect of IL-6 blockade on hematological abnormalities was partial.

**Conclusion:** TCZ improved fever and inflammatory response in VEXAS-RP and allowed steroid reduction. Future studies are needed to verify its safety with long-term use and its effects on hematological abnormalities and structural changes in chondritis.

**Disclosure of Interest**

None Declared

**Table 1 (abstract O15).** Clinical features of the VEXAS syndrome patients with RP using TCZ

Patient ID	RP13	RP15	RP16
Age at diagnosis (years)	66.3	73.5	66.6
*UBA1* variants p.Met41	c.122T>C: p.Met41Thr	c.122T>C: p.Met41Thr	c.121A>C: p.Met41Leu
Clinical findings at diagnosis	High-grade fever, skin rash, RP, scleritis, peritonitis, pericarditis, meningitis	High-grade fever, skin rash, RP, macrocytic anemia	High-grade fever, skin rash, GCA, RP, MDS, DVT, scleritis, airway involvement
Treatments before TCZ	PSL	PSL, MTX	PSL, AZP, colchicine
Symptoms existed at TCZ induction*	High-grade fever, myalgia, headache	Low-grade fever	High-grade fever, skin rash, RBC and PLT transfusion-dependence
PSL dose before TCZ administration	9 mg	22.5 mg	30 mg
PSL dose at last visit	1 mg	12.5 mg	12.5 mg
Duration of TCZ administration at last visit	12.0 months	6.3 months	7.1 months
Symptoms existed after 6 months	None	None	RBC transfusion-dependence, mild skin rash

RP; relapsing chondritis, GCA; giant-cell arteritis, Hb; hemoglobin, DVT, deep vein thrombosis; MDS, myelodysplastic syndrome; PSL, prednisolone; MTX; methotrexate, AZP, azathioprine; TCZ, tocilizumab, RBC; red blood cells, PLT: platelets, * symptoms existed during the past 1 month before TCZ induction

### O16 Genetic and immunologic characterization of male and female patients with nemo-deleted exon 5 autoinflammatory syndrome

#### A. A. de Jesus^1^, B. Lin^1^, E. Karlins^2^, D. Kahle^1^, A. Rastegar^1^, J. Mitchell^1^, S. Torreggiani^1^, F. Bhuyan^1^, S. Alehashemi^1^, K. Cetin Gedik^1^, K. Uss^1^, C.-C. Lee^3^, H. Kuehn^4^, S. Rosenzweig^4^, K. Calvo^4^, M. Walkiewicz^5^, J. Lack^6^, E. Hanson^7^, A. Khojah^8^, E. Wu^9^, C. Scott^10^, T. R. Leahy^11^, E. MacDermott^11^, O. Kileen^11^, T. Arkachaisri^12^, Z. Gucev^13^, K. Cook^14^, V. Mamadova^15^, G. Nasrullayeva^15^, M. Marques^16^, S. Canna^17^, S. Ozen^18^, D. Kuhns^19^, C. Dalgard^20^, T. Moran^9^, A. Oler^2^, R. Goldbach-Mansky^1^

##### ^1^Translational Autoinflammatory Diseases Section; ^2^BCBB, NIAID, NIH; ^3^NCI, NIH; ^4^DLM, NIH; ^5^CSI; ^6^NCBR, NIAID, NIH, Bethesda; ^7^Indiana University School of Medicine, Indianapolis; ^8^Lurie Children’s Hospital, Chicago; ^9^University of North Carolina School of Medicine, Chapel Hill, United States; ^10^University of Cape Town, Cape Town, South Africa; ^11^Children’s Health Ireland (CHI) at Crumlin, Dublin, Ireland; ^12^KK Women's and Children's Hospital, Kallang, Singapore; ^13^University Children's Hospital, Skopje, Republic of North Macedonia; ^14^Akron Children’s Hospital, Akron , United States; ^15^Azerbaijan Medical University, Baku, Azerbaijan; ^16^NIAMS, NIH, Bethesda; ^17^The Children’s Hospital of Philadelphia, Philadelphia, United States; ^18^Hacettepe University Faculty of Medicine, Ankara, Turkey; ^19^Frederick National Laboratory for Cancer Research, Frederick; ^20^The American Genome Center, Bethesda, United States

###### **Correspondence:** A. A. de Jesus

**Introduction:** Splice site variants in *IKBKG* that lead to exon 5 deletion cause NEMO-deleted exon 5 autoinflammatory syndrome (NEMO-NDAS). NEMO-NDAS clinically mimics the interferonopathy chronic atypical neutrophilic dermatosis with lipodystrophy and elevated temperature (CANDLE) but genetic diagnosis is challenging and treatment with JAK inhibitors provides only partial benefit.

**Objectives:** To characterize the immunodysregulation present in NEMO-NDAS patients (pts) and to validate a bioinformatics approach to diagnose these pts.

**Methods:** A bioinformatics pipeline to mask the *IKBKG* pseudogene was refined using splice prediction tools (SpliceAi, TraP, MaxEntScan and Human Splicing Finder). Screening of *IKBKG* exon 5 ± 30bp was validated in 701 samples from subjects enrolled in an IRB-approved protocol, and in internal (n=2655, whole exome sequencing (WES)) and public (n=2498, whole genome sequencing (WGS)) databases. The variants detected were validated by Sanger or amplicon deep sequencing (seq) and spliced product was confirmed by Western blot, cDNA seq and RNA-seq. Nanostring gene expression, PBMC stimulation and cytokine assays were performed. CRISPR generated U937 cell line clones were functionally assessed.

**Results:** 13 pts (9 females and 4 males), had 9 different de novo splice site variants in *IKBKG*. cDNA seq (13/13) or Western blot (5/5) confirmed the splice product in all pts tested. RNA-seq (n=12) showed a high frequency of exon 5 skipping (median 55% (35-80%)). *IKBKG* exon 5 splice site variants were screened in internal and public WES/WGS databases (n=5149) and 11 variants in 22 subjects passed filters. Sanger seq confirmed 1 of the 11 variants (9%); amplicon deep seq is pending. The most common clinical features in NEMO-NDAS pts were panniculitis with systemic inflammation (100%), ectodermal dysplasia (83%), hepatosplenomegaly (77%) and B-cell lymphopenia (80%). Compared to CANDLE pts (n=5), NEMO-NDAS pts’ skin biopsies (n=7) showed histiocytic panniculitis, vacuolar interface changes and dyskeratosis. Liver biopsies (n=3) showed granulomatous hepatitis; 2 other pts had portal hypertension. All pts were steroid-dependent with partial responses to anti-TNF (n=9) or JAK inhibition (n=9). Nanostring IFN and NF-κB scores were elevated in all 13 pts. Pts with NEMO-NDAS had higher serum levels of IFN-γ, IL-12p40, IL-17 and IL-23 than seen in CANDLE pts (p<0.0001 for all). Stimulated M1 and M2 macrophages from NEMO-NDAS pts (n=3) produced higher levels of CCL3/4 (MIP1-α/β) than healthy control (HC) cells (n=7)(p<0.05). NEMO-NDAS pts (n=2) had normal T and B cell proliferation, and Fas-induced T cell death was comparable to HC. U937 cell line clones lacking exon 5 normally degraded IκBα upon LPS stimulation and mutant U937 clones showed enhanced TNF induced cell death compared to wildtype and PSMB8-/- clones.

**Conclusion:** Our bioinformatics pipeline masking *IKBKG* pseudogene provides a sensitive diagnostic tool for the early recognition of NEMO-NDAS pts. The role of cytokine dysregulation and TNF induced cell death in the specific pathogenesis of NEMO-NDAS is being evaluated to improve therapeutic options.

**Acknowledgments**

This work was supported by the NIH Intramural Research Program (IRP) of NIAID

**Disclosure of Interest**

None Declared

### O17 Existing transition practices for autoinflammatory diseases across Europe

#### M. Israni^1^, B. Nicholson^1^, N. Mahlaoui^2^, L. Obici^3^, L. Rossi^4^, H. Lachmann^5^, G. Hayward^6^, M. Zajc Avramovič^7^, A. Guffroy^8^, V. Dalm^9^, R. Rimmer^10^, L. Solis^11^, C. Villar^12^, A. R. Gennery^13^, S. Skeffington^14^, J. Nordin^11^, K. Warnatz^15^, A. S. Korganow^8^, J. Antón^16^, M. Cattalini^17^, E. Morrison^18^, T. Amin^19^, P. Wekell^20^, S. Berg^21^, P. Soler-Palacín^22^, S. Burns^23^, M. Campbell^24^ on behalf of RITA-ERN Transition Working Group Consortium

##### ^1^Department of Immunology, Royal Free Hospital, London, United Kingdom; ^2^Pediatric Immuno-Haematology and Rheumatology Unit, Necker Enfants Malades University Hospital, Assistance Publique-Hôpitaux de Paris (AP-HP), Paris, France; ^3^Fondazione IRCCS Policlinico San Matteo, Centro per lo Studio e la Cura delle Amiloidosi Sistemiche, Pavia, Italy; ^4^Unité Policlinique pédiatrique, Hôpital Bicêtre, Assistance Publique-Hôpitaux de Paris (AP-HP), Paris, France; ^5^Division of Medicine, National Amyloidosis Centre , University College London, London; ^6^Paediatric and Adult Rheumatology, Leeds Teaching Hospital Trust, Leeds, United Kingdom; ^7^University Children's Hospital Ljubljana, Ljubljana, Slovenia; ^8^Department of Clinical Immunology and Internal Medicine, National Reference Center for Systemic Autoimmune Diseases (CNR RESO), Tertiary Center for Primary Immunodeficiency, Hôpitaux Universitaires de Strasbourg, Strasbourg, France; ^9^Department of Immunology, Erasmus University Medical Center, Rotterdam, Netherlands; ^10^Rare Autoinflammatory Conditions Community – UK (RACC – UK), England and Wales; ^11^IPOPI, Downderry, United Kingdom; ^12^Barcelona PID Foundation, Barcelona, Spain; ^13^Paediatric Haematopoietic Stem Cell Transplant Unit, Great North Children's Hospital (GNCH), Royal Victoria Infirmary, Newcastle upon Tyne, United Kingdom; ^14^Irish Vasculitis Organisation, Ireland, Ireland; ^15^Department of Immunology, Universitätsklinikum Freiburg, Freiburg, Germany; ^16^Department of Pediatric Rheumatology , Sant Joan de Déu Hospital, Barcelona, Spain; ^17^Pediatrics Clinic, University of Brescia and ASST Spedali Civili di Brescia, Brescia, Italy; ^18^Department of Rheumatology, Southern General Hospital, Glasgow; ^19^Children's Rheumatology, The Leeds Teaching Hospitals NHS Trust, Leeds, United Kingdom; ^20^NU-sjukvården - Barn- och ungdomskliniken , Uddevalla; ^21^Department of Pediatrics, Institute of Clinical Sciences, The Sahlgrenska Academy, University of Gothenburg, Gothenburg, Sweden; ^22^Pediatric Infectious Diseases and Immunodeficiencies Unit, Hospital Universitari Vall d’Hebron. Universitat Autonoma de Barcelona, Barcelona, Spain; ^23^Immunity and Transplantation, University College London; ^24^Department of Immunology, Royal Free London NHS Foundation Trust, London, United Kingdom

###### **Correspondence:** M. Israni

**Introduction:** Substantial improvements in the diagnosis and management of Autoinflammatory Diseases (AID) have translated into longer life expectancies among patients, thus prompting a need for defined transition protocols for long-term follow-up in adult care. Presently, there are limited data on existing transition programs for these disorders and their efficacy.

**Objectives:** This study aimed to examine the prevalence of transition programs and existing practices for the transition of young people with AID to adult services across European health centres. The results from this survey will be used to develop best practice guidelines for the transition of patients with AID.

**Methods:** A survey examining existing transition practices was developed by the European Reference Network on Rare Immunodeficiency, Autoinflammatory and Autoimmune Diseases Transition Working group and electronically circulated to paediatric AID centres across Europe.

**Results:** The survey generated 32 responses from AID centres spread across 29 cities in 13 countries. Majority of the respondents transitioned young patients to specialist AID services (24/31) or Adult Rheumatologists (10/31). However, approximately 10% (3/31) of the centres reported experiencing difficulties with identifying specialist adult centres for onwards referral - with 19% (6/31) of paediatric AID services transitioning patients to non-specialist Adult Internal Medicine Physicians.

Most AID centres started the transition process in early adolescence - with 55% (17/31) of the services beginning transition between the ages of 10 and 16. 61% (19/31) of the AID centres tended to end transition and transfer care to adult services after the age of 18.

Whilst 77% (24/31) of the services had an internal process for transition, only 20% (6/30) reported having access to national guidelines for transition. 68% (21/31) of the services offered patients joint appointments with multidisciplinary healthcare professionals from paediatric and adult services prior to transfer, and 73% (22/30) of the services described complete integration of medical records across centres.

Only 29% (9/31) of the paediatric services referred patients to adult centres with dedicated ‘young adult clinics' - with even fewer services transitioning patients to adolescent centres prior to adult care (16% or 5/31). Only 29% (9/31) of the AID centres reported transition-specific research programs at their site.

**Conclusion:** Whilst most paediatric AID centres followed an internal, integrated transition protocol, defined national and international guidelines are required to support successful transition of young patients to adult centres. Transition programs must be adapted to provide adolescent-friendly care that prepares young patients for transfer to adult care. Finally, the development of more transition-specific research programs is warranted to determine the predictors of successful transition and design effective transition interventions.

**Keywords:** transition, primary immunodeficiencies, autoinflammatory diseases, network.

**Disclosure of Interest**

M. Israni: None Declared, B. Nicholson: None Declared, N. Mahlaoui: None Declared, L. Obici: None Declared, L. Rossi: None Declared, H. Lachmann: None Declared, G. Hayward: None Declared, M. Zajc Avramovič: None Declared, A. Guffroy Grant /Research support from: CSL Behring and Takeda, V. Dalm: None Declared, R. Rimmer: None Declared, L. Solis: None Declared, C. Villar: None Declared, A. R. Gennery Grant /Research support from: Mallinckrodt and JAZZ Pharmaceuticals, S. Skeffington: None Declared, J. Nordin: None Declared, K. Warnatz: None Declared, A. S. Korganow Grant /Research support from: CSL Behring and Takeda, J. Antón Grant /Research support from: European Union, Insituto Carlos III, Fundación Daniel Bravo, Novartis, Sobi, Novimmune, Roche, Pfizer, Lilly, AbbVie and Amgen, Conflict with: Received personal fees or travel expenses from Novartis, Sobi, Roche, Pfizer, Lilly, AbbVie and Gebro, M. Cattalini Speaker bureau of: Novartis Farma, Abbvie, Sobi, and Pfizer , E. Morrison: None Declared, T. Amin: None Declared, P. Wekell: None Declared, S. Berg: None Declared, P. Soler-Palacín Grant /Research support from: European Union, Instituto Carlos III, Grifols and CSL Behring, Conflict with: Received personal fees or travel expenses from CSL Behring, Takeda and Grifols , S. Burns Grant /Research support from: European Union, National Institute of Health Research, UCLH and GOSH/ICH Biomedical Research Centers and CSL Behring, Conflict with: Received personal fees or travel expenses from Immunodeficiency Canada/IAACI, CSL Behring, Baxalta US Inc and Biotest, M. Campbell Grant /Research support from: National Institute of Health Research, PIDUK, CSL Behring and the Royal Free Charity, Conflict with: Received personal fees or travel expenses from Biotest, BPL, CSL Behring, Grifols, HAEUK, Shire and Takeda

### O18 COVID-19 infection and vaccination in patients with autoinflammatory diseases on biologics

#### C. J. Peet^1,2^, C. Papdopoulou^1^, B. R. Sombrito^1^, M. Wood^1^, H. J. Lachmann^1^

##### ^1^CAPS and Autoinflammatory Diseases Treatment Service, National Amyloidosis Centre, Royal Free London NHS Foundation Trust & Division of Medicine University College London; ^2^Department of Medical and Molecular Genetics, King's College London, London, United Kingdom

###### **Correspondence:** C. J. Peet

**Introduction:** Inflammatory cytokines are central to the pathogenesis of the systemic autoinflammatory diseases (SAID) and agents targeting these pathways have transformed the management of SAID. These same pathways are involved in the balance between viral clearance and hyperinflammation during infection and have therefore been areas of active research interest into COVID-19. As a result, among patients with SAID on biologics, there are legitimate concerns regarding the risk of COVID-19 infection and safety of vaccination, which are exacerbated by the scarcity of published data on the outcomes of COVID-19 infection and vaccination in these patients.

**Objectives:** This study establishes the prevalence and outcomes of COVID-19 infection and early outcomes of vaccination among a cohort of 248 patients with SAID on biologics.

**Methods:** Data were collected from the electronic medical records of 248 patients with SAID on biologics at a national centre up to 26/7/21. Patients were also surveyed using a web-based survey completed in clinic or remotely between 4/3/21 and 26/7/21.

**Results:** No deaths were recorded in this cohort of 248 patients between 29/1/20 and 26/7/21. In line with national guidance, all patients in this cohort were advised that the diagnosis of SAID managed with single-line biologic therapy was not an indication for shielding and that they should continue their biologic therapy unless otherwise instructed by a medical professional. Survey responses were received from 195 patients (195/248, 78.6%). Biologic therapy was as follows: anakinra (129/195, 66.2%), canakinumab (55/195, 28.2%), tocilizumab (8/195, 4.1%), etanercept (3/195, 1.5%), adalimumab (co-prescribed with anakinra) (1/195, 0.5%). Among survey respondents, 32 cases of suspected COVID-19 infection were reported, of which 15 were confirmed on testing (15/195, 7.7%). Two patients on anakinra were hospitalised due to dehydration. No patient required supplemental oxygen, mechanical ventilation or intensive care admission. Biologic therapy continued uninterrupted in all patients managed in the outpatient setting and was temporailty discontinued in the two hospitalised patients by the admitting team. Three patients with suspected or confirmed COVID-19 infection reported new symptoms that persisted for more than twelve weeks. 164/195 (84.1%) respondents had received the first vaccine dose and 79/195 (40.5%) had received two doses. 162/164 (98.8%) patients continued biologic therapy uninterrupted around the time of vaccination. Of the 243 vaccine doses administered, 138 (56.8%) were the Oxford Astra-Zeneca vaccine and 105 (43.2%) were the Pfizer-BioNTech vaccine. Following vaccination, no serious adverse events were reported and no patient required hospital admission. Side effects were reported following 130/243 (53.5%) doses and symptoms reported by patients to be in keeping with a flare of the underlying SAID were reported following 40/243 (16.5%). Side effects had fully resolved by 72 hours following 92.2% of doses (224/243). No cases of COVID-19 were reported following administration of both vaccine doses and only one early case was reported after the first dose, with symptom onset within five days of vaccine administration. Median follow up following vaccination was 10.5 weeks following the first dose and 6.0 weeks following the second.

**Conclusion:** This study shows COVID-19 infection rates broadly in line with that of the UK general population among patients with SAID on biologics and demonstrates no significant concerns regarding outcomes of infection in these patients. We also present the largest series of patients with SAID or on IL-1/6 biologics to have received an adenoviral vector or mRNA vaccine and observe no significant early safety concerns. Whilst longer follow-up is needed to establish the efficacy of vaccination in these patients, these findings will support patients and their clinicians to make informed decisions around continuation of biologic therapy and vaccination during the ongoing COVID-19 pandemic.

**Acknowledgments**

CJP is funded by a British Heart Foundation Clinical Research Training Fellowship.

**Disclosure of Interest**

C. J. Peet: None Declared, C. Papdopoulou: None Declared, B. R. Sombrito: None Declared, M. Wood: None Declared, H. J. Lachmann Consultant for: Novartis and Sobi

## Abstracts accepted for publication only

### P01 In pursuit of colchicine resistance prediction in familial mediterranean fever: exploring a novel scoring model and simultaneous validation

#### N. Aktay Ayaz^1^, F. G. Demirkan^1^, T. Coskuner^2^, F. Demir^2^, A. Tanatar^1^, M. Çakan^3^, S. G. Karadag^4^, G. Otar Yener^5^, S. Caglayan^6^, K. Ulu^7^, B. Sozeri^2^, H. E. Sonmez^8^

##### ^1^Pediatric Rheumatology, Istanbul University, Istanbul Faculty of Medicine; ^2^Pediatric Rheumatology, University of Health Sciences, Umraniye Research and Training Hospital; ^3^Pediatric Rheumatology, University of Health Sciences, Zeynep Kamil Women and Children's Diseases Training and Research Hospital; ^4^Pediatric Rheumatology, University of Health Sciences, Bakırkoy Dr. Sadi Konuk Training and Research Hospital, Istanbul; ^5^Pediatric Rheumatology, Sanlıurfa Training and Research Hospital, Sanlıurfa, Sanlıurfa; ^6^Pediatric Rheuamtology, University of Health Sciences, Umraniye Research and Training Hospital; ^7^Pediatric Rheumatology, University of Health Sciences, Umraniye Research and Training Hospital, Istanbul, Istanbul; ^8^Pediatric Rheumatology, Kocaeli University, Kocaeli School of Medicine, Kocaeli, Kocaeli, Turkey

###### **Correspondence:** N. Aktay Ayaz

**Introduction:** Over the years, definition and prediction of colchicine resistance have been the most intriguing issues of researches concerning familial Mediterranean fever (FMF).^1,2^

**Objectives:** The aim of this study is to develop a novel scoring system based on the initial clinical features and laboratory findings via synchronous validation of it in an independent group for predicting colchicine resistance in children with FMF.

**Methods:** The medical records of the cases with FMF who applied to the pediatric rheumatology outpatient clinic were evaluated retrospectively. To define the predictive factors for colchicine resistance, baseline clinical and laboratory findings prior to initiation of colchicine were evaluated. After generating a predictive score in the initial cohort, it was applied to an independent cohort in the database of Pediatric Rheumatology Academy (PeRA)-research group (RG) established in 2019^3^, for external validation of effectiveness and reliability.

**Results:** A total of 327 patients with FMF were included in the study. Among them, 78 (23.9%) (Group I) were colchicine resistant (cr)-FMF patients and 249 (76.1%) (Group II) were colchicine responsive. Erysipelas-like erythema (ELE), myalgia, arthritis, chronic arthritis, protracted febrile myalgia, amyloidosis, vasculitis, anemia, and proteinuria were more common in patients who were in group I than those in group II. A logistic regression model was used to estimate a model to predict the colchicine resistance in FMF patients. According to the regression analysis, *recurrent arthritis, protracted febrile myalgia, presence of ELE, chronic anemia and elevated serum amyloid A levels* in the attack-free period were determined as the scoring parameters for predicting patients with cr-FMF (Table 1). Scores were assigned according to β coefficients in the final model. The cut-off value of predictor score as 4.5 was 88% sensitive and 82% specific to foresee the risk of colchicine resistance in the ROC.

The predictive score was applied to 374 patients (colchicine resistant = 75, colchicine responsive = 299) which were registered to PeRA-RG database up to December 2020. Sixty-five (86.6%) cr-FMF patients and 3 (1%) colchicine responsive patients had a total score of more than 4.5. The cut-off value of the score as 4.5 was 86.6% sensitive and 99% specific to identify the risk of colchicine resistance in the ROC.

**Conclusion:** In advance of the previous studies, we intended to design a composite predictive scoring system to foresee unresponsive patients.^4^ By constructing this novel reliable predictor tool, we enunciate that physicians will be capable of anticipating drug resistance in children with FMF at the initiation of the disease and, thereby have a chance to interfere timely before the emergence of complications during the disease course.

**Key Words:** Familial Mediterranean fever, predictive score, colchicine resistance

**REFERENCES**

**1.** Ozen S, Kone-Paut I, Gül A. Colchicine resistance and intolerance in familial mediterranean fever: Definition, causes, and alternative treatments. Seminars in arthritis and rheumatism. 2017;47(1):115-20.

**2.** Babaoglu H, Armagan B, Bodakci E, Satis H, Atas N, Sari A, et al. Predictors of persistent inflammation in familial Mediterranean fever and association with damage. Rheumatology (Oxford, England). 2021;60(1):333-9.

**3.** Betul Sozeri HES, Ferhat Demir, Mustafa Çakan, Kübra Öztürk, Semanur Özdel, Gulcin Otar Yener, Şerife Gül Karadağ, Nuray Aktay Ayaz. Time to collaborate: Objectives, Design, and Methodology of PeRA-Research Group. The North Clinics of Istanbul. 2020.

**4.** Özçakar ZB, Elhan AH, Yalçınkaya F. Can colchicine response be predicted in familial Mediterranean fever patients? Rheumatology (Oxford, England). 2014;53(10):1767-72.

**Disclosure of Interest**

None Declared

**Table 1 (abstract P01).** Multivariate logistic regression analysis of prediction of colchicine resistance and scoring system

Predictors	Odds Ratio (95% CI)	***P*** value	Score assigned
Recurrent arthritis	2.6 (0.99-7.02)	0.03	2
Protracted febrile myalgia	6.8 (1.43-32.34)	0.01	6
Erysipelas-like erythema	2.3 (0.92-6.21)	0.02	2
Anemia	3.4 (1.26-9.65	0.02	3
Elevated SAA in the attack free period	0.95 (0.91-1.01)	0.04	1

*SAA, Serum amyloid A*

### P02 Diagnostic delay in a rare autoinflammatory syndrome

#### F. Annunziata^1^, M. L. Pennacchio^1^, M. Tardi^2^, F. Paciello^1^, R. Borrelli^2^, R. Naddei^1^, M. Alessio^1^, L. Martemucci^2^, F. Orlando^2^

##### ^1^Department of Translational Medical Sciences, Section of Pediatrics, University of Naples Federico II; ^2^Pediatrics, AORN Santobono Pausilipon, Naples, Italy

###### **Correspondence:** F. Annunziata

**Introduction:** TRNT1 is a nuclear gene encoding a ubiquitous enzyme (CCA-adding tRNA nucleotidyltransferase enzyme) necessary for aminoacylation of both mitochondrial and cytosolic tRNA. Mutations of that gene lead to heterogeneous phenotypes and systemic involvement of variable severity and progression.

**Objectives:** To consider mutations of TRNT1 as an important differential diagnosis in patients with overlap of immunodeficiency and autoinflammatory features to avoid diagnostic delay.

**Methods:** A 10-year-old female admitted to our rheumatology pediatric unit of Santobono Children's Hospital of Naples for febrile illness associated with vomit and diarrhoea, evolved in shock. She was treated in intensive care with broad spectrum antibiotics and cardiovascular support.

**Results:** The anamnesis revealed recurrent fever accompanied by vomit, metabolic acidosis, dyselectrolytemia and increase of inflammatory markers, without evidence of infective causes, since second month of life. Even in the absence of evidence of infective origins, these febrile episodes were often treated with systemic antibiotic therapy with poor clinical response. However, it was observed resolution of symptoms after steroids therapy. She presented to our attention with following clinical conditions: microcytic anaemia (requiring blood transfusion),bilateral cataract, hypotonia, intellectual disability andhypogammaglobulinemia. Physical examination documented occurrence of facial dysmorphisms,brittle hair and intellectual disability. At the admission, laboratory assessment showed: white blood cell 4920/ul (n.v. 5-19), Haemoglobin 9.4 g/dl (n.v. 10.18), MCV 60.9 fL (85-120), RDW 43 fL (n.v. 38.7-45.1), Platlets 119000/ul (n.v. 140-440), Neutrophiles 3870/uL (n.v. 1300-8500), Lymphocytes 619/uL (n.v.1300-8500), Eosinophiles50/uL (n.v. 0-80), C Reactive Protein 324 mg/l (n.v. 0.5), Procalcitonin 610 ng/ml (n.v. <0.5), Ferritin 2071 ng/ml (n.v. 6-67), lactic dehydrogenase 1198 U/l (n.v. 230-500). Immunological screening was performed, showing hypogammaglobulinemia, with IgA <0.22 g/l (n.v 0.5-3) and IgG 6,3 g/dl (n.v. 7-15), and low levels of T total (TCD3 49%, n.v.55-78%) and T helper lymphocites (TCD4 29%, n.v. 27-53%). No infective causes were found. Because of the history characterized by recurrent fever, immunological alterations and dismorphic appearance, TRNT1 mutation associated disease was suspected. Genetic analysis was done, confirming our suspicion.Based on case reports available in literature, after the molecular diagnosisshe started onanti-TNF alfa (Etanercept) therapy.

**Conclusion:** The presence of recurrent fever associated with elevation of inflammatory indexes, without evidence of infections, led to consider TRNT1 mutation related disease as an autoinflammatory syndrome. Currently, 49 case are reported in literature. This condition was firstly associated to congenital sideroblastic anaemia with immunodeficiency, fevers, and developmental delay (SIFD) but over the years phenotypic heterogeneity was described. Due to the rarity of that syndrome, dignostic delay is observed, as in our patient. Early diagnosis of this condition would enable patients to promptly access to therapies. Symptomatic treatments, including red blood cells transfusions, immunoglobulin replacement therapy and steroids, are the most used; however, mortality is high. Etanercept has been recently described as effective treatment. TNFa inhibitors can lead to a significative response for those patients who can benefit early in life. Our patient started that treatment but we are still waiting to define the clinical benefits of this therapy.

**Disclosure of Interest**

None Declared

### P03 Long-term efficacy and safety of canakinumab in patients with traps (tumor necrosis factor receptor-associated periodic syndrome) - interim analysis of the reliance registry

#### N. Blank^1^, J. Henes^2^, T. Kallinich^3^, P. T. Oommen^4^, C. Schuetz^5^, M. Borte^6^, J. Weber-Arden^7^, J. Kuemmerle-Deschner^2^

##### ^1^University Hospital, Heidelberg; ^2^University Hospital, Tuebingen; ^3^Charité University Medicine, Berlin; ^4^University Hospital, Duesseldorf; ^5^Medizinische Fakultaet Carl Gustav Carus, Dresden; ^6^Hospital St. Georg gGmbH, Leipzig; ^7^NOVARTIS PHARMA, Nürnberg, Germany

###### **Correspondence:** N. Blank

**Introduction:** Tumor necrosis factor receptor-associated periodic syndrome (TRAPS) is a rare autoinflammatory condition characterized by severe systemic and organ inflammation. In clinical trials TRAPS patients have been successfully treated with the interleukin-1β inhibitor canakinumab. Canakinumab has been approved and applied for the treatment of TRAPS patients since 2017.

**Objectives:** The present study explores the long-term efficacy and safety of canakinumab under routine clinical practice conditions in pediatric (age ≥2 years) and adult TRAPS patients.

**Methods:** RELIANCE is a prospective, non-interventional, multi-center, observational study based in Germany with a 3-year follow-up period. Patients with clinically confirmed diagnoses of TRAPS who routinely receive canakinumab are enrolled in order to evaluate efficacy and safety of canakinumab under standard clinical practice conditions. Inflammatory markers, disease activity and remission by physician assessment, disease activity and fatigue by patient assessment, were assessed at baseline and at 6-monthly intervals.

**Results:**

The interim analysis of TRAPS patients enrolled by December 2020 includes baseline (N=16, including 1 patient with atypical TRAPS) and preliminary 18-month data. Mean age in this cohort was 23 years (3-43 years) and the median duration of prior CAN treatment was 1.0 year (0-4 years). All patients already were on canakinumab when being enrolled. Prior treatments were colchicin (N=2), anakinra (N=10) and tocilizumab (N=1).

Physician assessment indicated 60-80% remission and laboratory parameters were within normal range. Disease control by patient assessment showed no major changes regarding the analyzed parameters (table 1). Of the three serious adverse events reported none was classified as drug-related.

**Conclusion:** Preliminary analysis of 18-month interim data of TRAPS patients treated with CAN available from the RELIANCE study indicate stable efficacy and safety of CAN long-term treatment.

**Table 1 (abstract P03).** Baseline characteristics and interim analysis data of patients with TRAPS

	Baseline	6 months	12 months	18 months
Number of patients, N	16	13	10	6
Females (%)	11 (69)	9 (69)	7 (70)	3 (50)
Median duration of prior CAN therapy at baseline, years (min; max)	1.0 (0; 4)	1.0 (0; 4)	1.0 (0; 4)	1.5 (0; 2)
Number (%*) of patients in disease remission (physician assessment)	9 (60.0)	9 (81.8)	7 (77.8)	4 (80.0)
Physician Global Assessment, percentage of absent/mild-moderate/severe rating	40 / 53 / 0	82 / 9 / 0	44 / 44 / 11	80 / 20 / 0
Patient assessment of current disease activity; 0–10, median (min; max)	1.5 (0; 5)	1.0 (0; 4)	1.0 (0; 6)	0.0 (0; 3)
Patient assessment of current fatigue; 0–10, median (min; max)	2.0 (0; 8)	1.0 (0; 7)	2,5 (0; 8)	4.0 (0; 7)
Number (%*) of patients without impairment of social life by the disease	4 (50)	5 (63)	2 (33)	3 (60)
CRP, SAA median (mg/dl)	0.1/0.5	0.1/0.4	0.1/0.4)	0.0/0.3

*not reported for all patients

CRP, c-reactive protein; SAA, serum amyloid A

**Disclosure of Interest**

N. Blank Grant /Research support from: Novartis, Sobi, Consultant for: Novartis, Sobi, Lilly, Pfizer, Abbvie, BMS, MSD, Actelion, UCB, Boehringer-Ingelheim, Roche, J. Henes Grant /Research support from: Novartis, Roche, Consultant for: Novartis, AbbVie, Sobi, Roche, Janssen, Boehringer-Ingelheim, T. Kallinich Grant /Research support from: Novartis, Consultant for: Sobi, Novartis, Roche, P. T. Oommen Grant /Research support from: Novartis, C. Schuetz: None Declared, M. Borte: None Declared, J. Weber-Arden Employee of: Novartis, J. Kuemmerle-Deschner: None Declared

### P04 Evaluation of E148Q and concomitant AA amyloidosis secondary to familial mediterranean fever after adjusted clinical-demographic characteristics

This abstract has not been included here as it has been previously published

### P05 Non-urticarial Muckle-Wells syndrome and mitochondrial cytopathy – hand-in-hand in the NLRP3 inflammasome

#### A. Lamas^1^, D. G Oliveira^1,2^, M. Rodrigues^3^, R. Faria^2,4^

##### ^1^Medicine, Centro Hospitalar e Universitário do Porto; ^2^UMIB, ICBAS - Universidade do Porto, Porto; ^3^Neurology Department, Hospital de Braga, Braga; ^4^Consulta de Sindromas Autoinflamatórios, Unidade de Imunologia Clínica, Centro Hospitalar e Universitário do Porto, Porto, Portugal

###### **Correspondence:** D. G Oliveira

**Introduction:** Muckle-Wells syndrome (MWS) is a rare inherited cryopyrin-associated periodic syndrome (CAPS) with NLRP3 gain-of-function mutations. Although urticaria is a prominent clinical feature, cases have been reported with systemic and neurological involvement without cutaneous manifestations. Inflammasome activation is a complex process and there is mounting evidence of the key role played by mitochondria.

**Objectives:** To describe the clinical phenotype of two adult sisters harbouring a heterozygous NLRP3 mutation (c.2582A>G p.(Tyr861Cys) and a homoplasmic MT-TS2 mitochondrial variant of uncertain significance (VOUS) (m.12236G>A).

**Results:** The patients described are siblings from a sibship of 5 (1 male and 4 females), in a family with a history of sensorineural deafness (SND) (at least 3 generations affected, onset during adolescence). A niece was diagnosed with MWS without mitochondrial mutation at another centre.

Patient 1: a 52-year-old woman with a history of SND and chronic headaches (onset at age 10), associated with nausea, vomiting and fever (mostly concomitant with headaches), recurrent aseptic meningitis and stroke-like episodes. Other features included recurrent conjunctivitis, polyarthritis, inflammatory/iron deficiency anaemia and accelerated atherosclerosis (myocardial infarction at age 43). There was no history of cutaneous involvement. Patient 2: a 46-year-old woman with a history of SND (onset at age 18), presented with chronic recurrent headaches (onset at age 15), with concomitant nausea, vomiting and fever, with documentation of aseptic meningitis, and stroke-like episodes. Polyarthritis, synovitis and enthesopathy were more severe, and clinodactyly was present. There was no history of cutaneous involvement. Other features included recurrent conjunctivitis and inflammatory/iron deficiency anaemia.

Both presented with markedly elevated serum amyloid A, erythrocyte sedimentation rate and C-reactive protein. Serum lactate levels were normal. Cerebrospinal fluid analysis revealed elevated protein levels, with associated pleocytosis. Electromyography was normal and muscle biopsy showed no evidence of mitochondrial myopathy. Abdominal fat biopsy showed no evidence of amyloid deposition.

PCR and Sanger sequencing detected a homoplasmic mitochondrial VOUS in MT-TS2 gene (m.12236G>A) and a heterozygous missense NLRP3 mutation (c.2582A>G p.Tyr861Cys, Y861c or previously known as Y859c – in mice). Extensive genetic studies for the exclusion of other mitochondrial cytopathies were performed.

A diagnosis of MWS syndrome was assumed and treatment started with IL-1 receptor antagonist (anakinra), with significant clinical improvement (headaches, fever, arthritis) and inflammatory marker remission.

**Conclusion:** The clinical features fulfil diagnostic criteria for CAPS, as proposed by *Kuemmerle-Deschner et al.* (2016). They are also compatible with the phenotype previously described for this exon 6 missense mutation, coding for a protein at Y861, a dephosphorylation site in the leucine-rich repeat (LRR) domain of NLRP3. Phosphorylation/dephosphorylation of NLRP3 at key sites, as Y861, has been shown to be an essential step for the regulation of the NLRP3 inflammasome, with Y861c mutation leading to spontaneous inflammasome activation and interleukin-1β hyperproduction.

The mitochondrial VOUS m.12236G>A is associated with non-syndromic SND, MELAS syndrome (*mitochondrial encephalomyopathy, lactic acidosis, and stroke-like episodes)* and respiratory chain deficiency (affecting complexes I, III and IV). Both patients did not fulfil diagnostic criteria for MELAS syndrome (*Hirano et al*. (1992) or *Yatsuga et al*. (2012)), an insufficient hypothesis to explain all the clinical manifestations. Mitochondrial dysfunction with increased production of reactive oxygen species and oxidized mitochondrial DNA (mtDNA) may act synergically in NLRP3 activation, autoinflammation and “inflammaging”. This case illustrates the intricacies of NLRP3 inflammasome hyperactivation through a rare mutation in exon 6, resulting in a distinct clinical phenotype. It also underlines the complex interplay between mitochondria and NLRP3 activation, and its potential role in the modulation/definition of clinical phenotypes.

**Disclosure of Interest**

None Declared

### P06 Long-term safety and effectiveness of canakinumab in patients with familial mediterranean fever (FMF) interim analysis of the reliance registry

#### J. Henes^1^, J. Kümmerle-Deschner^2^, T. Kallinich^3^, F. Dressler^4^, F. Weller-Heinemann^5^, B. Kortus-Goetze^6^, I. Foeldvari^7^, G. Horneff^8^, M. Hufnagel^9^, F. Meier^10^, J. Weber-Arden^11^, N. Blank^12^

##### ^1^University Hospital, Tuebingen, Germany; ^2^Pediatric Department, University Hospital, Tuebingen; ^3^Charité University Medicine, Berlin; ^4^Hannover Medical School, Hannover; ^5^Prof. Hess Kinderklinik, Bremen; ^6^University Hospital, Marburg; ^7^Centre for Pediatric and Adolescence Rheumatology Hamburg, Hamburg; ^8^Asklepios Clinic , Sankt Augustin; ^9^University Hospital, Freiburg; ^10^University Hospital, Frankfurt; ^11^NOVARTIS PHARMA, Nürnberg; ^12^University Hospital, Heidelberg, Germany

###### **Correspondence:** J. Henes

**Introduction:** Familial Mediterranean Fever (FMF) is characterized by recurrent attacks of fever and serositis as well as elevated inflammatory markers. FMF treatment goals according to EULAR are to control acute attacks and subclinical inflammation and to improve patients´ quality of life.

**Objectives:** The present study explores the long-term efficacy and safety of canakinumab (CAN) in routine clinical practice in pediatric (age ≥2 years) and adult FMF patients.

**Methods:** RELIANCE is a prospective, non-interventional, multi-center, observational study based in Germany for patients with clinically confirmed FMF who routinely receive CAN. Disease activity parameters by physicians´ and patients’ assessment were recorded at baseline and were assessed at 6-monthly intervals.

**Results:** This interim analysis of FMF patients (N=54) enrolled by December 2020 includes baseline as well as 6-, 12- and 18-month data. Mean age in this cohort was 25 years (4-56 years) and the proportion of female patients was 46 % (N=25). At baseline, median duration of prior CAN treatment was 2.0 years (0-6 years). 34 patients (63%) were concomitantly treated with colchicine.

While physician ratings report around 62% of patients in disease remission, 52% with absent and 34% with mild-moderate disease activity, patient-reported disease activity decreased during the observation period, especially in patients without prior CAN therapy (table 1). A total of 11 serious adverse events was reported, of which one case of tonsillectomy in a 6-year-old patient was classified as drug-related.

**Conclusion:** Interim data of FMF patients from the RELIANCE study confirm efficacy and safety of long-term CAN treatment.

**Table 1 (abstarct P06).** Baseline characteristics and third interim analysis data of patients with FMF

	Baseline		6 months		18 months	
All patients | Patients without prior CAN therapy	All patients	Patients without prior CAN	All patients	Patients without prior CAN	All patients	Patients without prior CAN
Number of patients, N	54	11	35	7	16	3
Number (%*) of patients in disease remission (physician assessment)	18 (48.6)	1 (20.0)	19 (73.1)	3 (75.0)	8 (61.5)	1 (100.0)
Patient assessment of current disease activity; 0–10, median (min; max)	3.0 (0; 10)	7.0 (0; 10)	2.5 (0; 7)	2.0 (0; 5)	2.0 (0; 6)	0.5 (0; 1)
Patient assessment of current fatigue; 0–10, median (min; max)	5.0 (0; 10)	5.0 (0; 9)	3.5 (0; 10)	3.0 (1; 6)	3.0 (0; 7)	0.5 (0; 1)
Number (%*) of patients without impairment of social life by the disease	19 (46.3)	3 (37.5)	18 (66.7)	3 (75.0)	5 (55.6)	2 (66.7)
CRP/SAA, median (mg/dl)	0.2/0.7	1.1/6.8	0.2/0.8	0.1/0.4	0.1/0.6	0.5/0.7

*not reported for all patients

CRP, c-reactive protein; SAA, serum amyloid A

**Disclosure of Interest**

J. Henes Grant /Research support from: Novartis, Roche, Consultant for: Novartis, AbbVie, Sobi, Roche, Janssen, Boehringer-Ingelheim, J. Kümmerle-Deschner Grant /Research support from: Novartis, AbbVie, Sobi, Consultant for: Novartis, AbbVie, Sobi, T. Kallinich: None Declared, F. Dressler Grant /Research support from: Novartis, Consultant for: Abbvie, Mylan, Novartis, Pfizer, F. Weller-Heinemann: None Declared, B. Kortus-Goetze Consultant for: Novartis, I. Foeldvari Consultant for: Novartis, G. Horneff Grant /Research support from: AbbVie, Chugai, Merck Sharp & Dohme, Novartis, Pfizer, Roche, Speaker bureau of: AbbVie, Bayer, Chugai, Merck Sharp & Dohme, Novartis, Pfizer, Roche, M. Hufnagel Grant /Research support from: Novartis, F. Meier Speaker bureau of: Novartis, J. Weber-Arden Employee of: Novartis, N. Blank Grant /Research support from: Novartis, Sobi, Consultant for: Novartis, Sobi, Lilly, Pfizer, Abbvie, BMS, MSD, Actelion, UCB, Boehringer-Ingelheim, Roche

### P07 A family with an autosomal dominant CDC42-mutation covering the full clinical spectrum from developmental defects to autoinflammation

#### V. Hentgen^1^, B. Bekhouche ^2^, A. Terre-Becker^2^, J. Cherfils^3^, M. Andre^4^, S. Georgin-Lavialle^5^, G. Boursier^6^, A. Smahi^2^

##### ^1^Reference Center for AutoInflammatory diseases and amyloidosis, Versailles Hospital, Le Chesnay; ^2^INSERM U1163, Imagine Institute, Paris; ^3^Laboratoire d’Enzymologie et Biochimie Structurales, CNRS, Gif-sur-Yvette; ^4^Service de médecine interne, Montpied Hospital, Clermont-Ferrand; ^5^Reference Center for AutoInflammatory diseases and amyloidosis, Tenon Hospital, Paris; ^6^Laboratory of Rare and Autoinflammatory Genetic Diseases, CHRU Montpellier, Montpellier, France

###### **Correspondence:** V. Hentgen

**Introduction:** The *CDC42* (Cell Division Cycle 42) gene product, CDC42, is a member of the family of small Rho GTPases, which are implicated in a broad spectrum of physiological functions in cell cycle regulation. Missense variants in *CDC42* underlie a clinically heterogeneous group of phenotypes characterized by variable neurodevelopmental, growth, hematological, and immunological disorders.

**Objectives:** Here, we report a family of 4 affected members with a dominant missense mutation in the *CDC42* gene and analyze the pathophysiological mechanisms explaining a large part of the phenotype

**Methods:** The family (a mother with 3 of her children) was referred to our reference center for a suspected tumor necrosis factor receptor-associated periodic syndrome. No pathogenic variant was identified in 8 autoinflammation-related genes including *TNFRSF1A*, *NLRP3* and *MVK*. Affected members presented with recurrent fevers lasting several weeks, severe arthromyalgias, arthritis and splenomegaly. Two children had in addition labio-palatine cleft and one developmental delay. All the affected members presented with other slight dysmorphic signs: hypertelorism, wide nasal bridge and mouth with widely spaced teeth. Inflammatory manifestations responded remarkably well to IL-1beta inhibition. Patients were included to the France Genomique research program allowing us to perform whole genome sequencing in trio and investigate the pathophysiological mechanisms.

**Results:** A novel heterozygous p.Thr43Ile mutation in the *CDC42* gene was identified in all affected family members. *In silico* modelisation predicted no structural change of CDC42, but a probable impaired interaction with DOCK proteins, a family of proteins involved in intracellular signaling networks. Fibroblasts as well as Peripheral Blood Mononuclear cells (PBMC) from the patients showed an inflammatory signature with an increased expression of IL-1β, IL-6, TNFα, IL-8 and IL-18 on a basal state in the untreated patient and after stimulation in treated ones. In respect to PBMC controls, patients showed an over-activation of both pyrine and NLRP3 inflammasomes using both agonist triggers and specific inibitors (NLRP3 inflammasome inhibitor MCC 112 and pro-caspase-1 inhibitor Z-VAD).The spontaneous increased ASC/Speck aggregation in mutated PBMCs confirms this observation strongly.

These observations allow us to hypothesize that the inflammatory profile of the *CDC42* mutations, passes through abnormal activation of the pyrin and probably the NLRP3 inflammasome thus linking the *CDC42* abnormalities to the same inflammatory pathways than familial Mediterranean fever and mevalonate kinase deficiency.

**Conclusion:** We describe in four affected members of the same family a novel heterozygous dominant p.Thr43Ile mutation in the *CDC42* gene. *CDC42* encodes a small GTPase of the Rho subfamily, playing a pivotal role in cell cycle regulation. In the family studied, diverse clinical manifestations compose a syndromic phenotype with autoinflammation, facial dysmorphism and neurodevelopmental delay. The pathophysiological assessment showed that the inflammatory profile of the p.Thr43Ile *CDC42* mutation is linked to the pyrin and to a lesser extend to the NLRP3 inflammasome, unravelling a heretofore-unrecognized connection between seemingly unrelated autoinflammatory diseases. CDC42 mutations should be searched for USAID patients with splenomegaly, recurrent arthralgia and a clinical response to IL1 blockers, sporadic or dominant transmission.

**Disclosure of Interest**

None Declared

### P08 Evaluation of fatigue level and sleep quality in patients with familial mediterranean fever

#### Ç. İncesu^1^, G. Kavrul Kayaalp^2^, F. G. Demirkan^2^, O. Köker^2^, F. Çakmak^2^, Ö. Akgün^2^, N. Aktay Ayaz^2^, R. Eker Ömeroğlu^2^

##### ^1^Pediatrics; ^2^Pediatric Rheumatology, Istanbul University, Faculty of Medicine, İstanbul, Turkey

###### **Correspondence:** G. Kavrul Kayaalp

**Introduction:** Familial Mediterranean fever (FMF) is the most prevalent hereditary autoinflammatory disease. It is characterized by recurrent inflammatory episodes of fever and serositis. Its lifelong and inflammatory nature can cause detrimental effects on all aspects of patients' quality of life. Sleep quality and fatigue are essential factors affecting quality of life, which may be impaired in FMF patients due to recurrent episodes or ongoing subclinical inflammation.

**Objectives:** In this study, it is aimed to evaluate whether sleep quality and fatigue level are affected in children with FMF compared to healthy children and to assess the parameters that may affect sleep quality and fatigue status in FMF patients.

**Methods:** 225 children aged 7- 18 years who are followed up with the diagnosis of FMF in Istanbul University Faculty of Medicine Pediatric Rheumatology Clinic were included in the study. 182 age matched healthy peers were included as control group. Demographic features, family history, *MEFV* gene mutations, medications and The International Severity Score for FMF (ISSF) scores of the patients were recorded. The PedsQL Multidimensional Fatigue Scale module which consists of three parts as general fatigue, sleep/rest-related fatigue and cognitive fatigue was used to assess fatigue. Five answer options were offered and raw scores were extrapolated to a scale of 0 to 100 reversely (0 converted to 100, 1 to 75, 2 to 50, 3 to 25 and 4 to 0). Pittsburgh Sleep Quality Index (PSQI) was used to assess sleep quality. The scores ranged 0 to 21were calculated according to the calculation sheet. A total score of 5 and above was considered as poor sleep quality.

**Results:** Gender distribution was 59.3% female and 40.7% male in the patient group and 51.6% female, 48.4% male in the control group. The mean age was 12.2 ± 2.4 years, and 12.1 ± 3.3 years in the patient and control groups respectively. Mean ISSF score was 2.2 ± 1.1 points. Medications of the patients were 88.4% colchicine, 11.6% colchicine and anti IL-1 treatment (canakinumab or anakinra). The *MEFV* mutations were homozygous in 25.8%, heterozygous in 38.2%, compound heterozygous in 28% of the patients group. In the last year 13.3% of the patients didn’t have any attack, 86.7% had at least 1 attack. The median PSQI score was significantly higher in the patient group (5.1±2.3 in the patient group, 3.2±1,3 in the control group; p <0.05) and the median PEDsQL score was significantly higher in the control group (66.0±17.3 in the patient group, 70.4±15.9 in the control group; p <0.05). A significant positive correlation was observed between the number of attacks in the last 1 year and the PSQI score (r=0.721, p <0.05). There was no significant difference in terms of ISSF score, PSQI score, PEDsQL score, general fatigue score, sleep/rest fatigue score, and thought fatigue score between homozygous, heterozygous and compound heterozygous groups.

**Conclusion:** This study demonstrates that children with FMF have lower sleep quality and higher fatigue levels compared to their healthy peers. The disease activity also effects the sleep quality and fatigue status of FMF patients. Maintaining low disease activity in the of the patients with FMF and evaluation of sleep quality in the routine follow-ups can improve the quality of life of the patients.

**Disclosure of Interest**

None Declared

### P09 Next generation sequencing in diagnosis of periodic fevers: one center experience

#### Z. Korobova^1,2^, I. Svitina^2^, K. Belozerov^2^, A. Tumakova^2^, A. Romanko^3^, E. Suspitsyn^2,3^, M. Kostik^2,4^

##### ^1^Saint Petersburg Pasteur Institiute; ^2^Saint Petersburg State Pediatric Medical University; ^3^N.N. Petrov National Medical Research Center of Oncology; ^4^Almazov National Medical Research Centre, Saint-Petersburg, Russian Federation

###### **Correspondence:** Z. Korobova

**Introduction:** Periodic fever syndromes (PFS) is a group of disorders characterized by recurrent attacks of systemic and organ-specific inflammation. Establishing a reliable diagnosis on clinical and laboratory grounds is difficult due to non-specific clinical presentation. Besides monogenic autoinflammatory conditions periodic fevers may be a component of other primary immunodeficiency syndrome (PIDs), rheumatic diseases or cancer.

**Objectives:** To evaluate benefits of next generation sequencing (NGS) in patients with periodic or persistent fevers and no evidence of infectious disease.

**Methods:** 55 patients with suspected autoinflammatory PFS were tested for genetic mutations in 344 PID-associated genes using targeted NGS.

**Results:** Out of 55 patients, 53% (n=29) presented periodic fevers (recurrent rises of body temperature over 38.0°C), 44% (n=24) had persistent fevers (i.e. lasting rises of body temperature over 38.0°C for at least 3 weeks). Two patients had clinical and laboratory signs of autoinflammatory disease but no fever. Other symptoms included: local or generalized lymphadenopathy (n = 23; 42%), rash (n=29; 53%), arthritis (n=36; 65%), vasculitis (n=17; 31%), serositis (n=17; 31%). We noticed a rise of inflammatory laboratory markers such as ESR, CRP, leukocyte numbers in all the patients.

Based on the results of targeted NGS, we divided the patients into 3 groups:

Group I (n = 25) included patients with mutations associated with autoinflammatory diseases. These included mutations in MEFV (n=10), TNFRSF1A (n=6), NLRP12 (n=5), TNFAIP3 (n=2), MVK (n=1), NOD2 (n=1).

Group II (n=5) included patients with mutations in the genes associated with other PIDS: WAS, NFKB2 (n=2), CTLA4 and a MBL2.

Group III included patients (n = 25) with no mutation identified. This group included patients with a systemic juvenile idiopathic arthritis, undifferentiated systemic vasculitis and systemic connective tissue diseases.

We compared clinical and laboratory findings in patients of groups II and III with those of group I. No significant difference in age of onset, clinical manifestation or laboratory activity was found.

**Conclusion:** Usage of NGS makes it possible to find causative mutations - in those cases where they are detected it is easier to find better therapeutic options.

**Acknowledgments**

This work was supported by the Russian Foundation for Basic Research (grant № 18-515-57001).

**Disclosure of Interest**

None Declared

### P10 Long-term efficacy and safety of canakinumab in Cryopyrin-Associated Periodic Syndromes (CAPS) – 30-month data from the reliance registry

This abstract has not been included here as it has been previously published

### P11 Autoimmunity within autoinflammatory diseases: not so far, not so rare

#### J. R. Marques Soares^1^ on behalf of Autonflammatory Diseases Unit - Hospital Vall Hebron, M. Martinez Gallo^2^, M. Antolin Mate^3^, S. Bujan Rivas^1^ on behalf of Autoinflammatory Diseases Unit - Hospital Vall Hebron

##### ^1^Internal Medicine; ^2^Immunology; ^3^Genetics, Hospital Vall D´Hebron (Barcelona) Spain, Barcelona, Spain

###### **Correspondence:** J. R. Marques Soares

**Introduction:** Autoinflammation and autoimmunity are two sides of the immune system. Altought phisiopathological mechanism are supposed to be clearly different, some autoimmune features have been identified as signifcative parts of new entities like SAVI, DADA2 or COPA syndrome. In some cases, the clinical and antibody profile in some autoinflammatory diseases can remind autoimmune conditions.

**Objectives:** To evaluate the presence of sera autoantibodies and organ-specific / systemic autoimmune diseases according to the international clasification criteria among a cohort of autoinflammatory adult patients followed at a reference unit of autoinflammatory diseases froma thir-care centre.

**Methods:** Retrospective review of clinical and laboratory reports of a cohort of 75 adult patients followed at the Autoinflammatory Diseases Unit from Hospital Vall Hebron (Barcelona, Spain). Demographic, clinical and immunological laboratory data were collected.

**Results:** The mean age of the cohort was 46 y with a female/male ratio 0.875. All but one patient were caucasian.

The SAIs of the cohort were as follow: FMF 33 (44%), TRAPS 9 (12%), MKD 2 (2.6%), CAPS 10 (13.3%), PFAPA 8 (10.6%), indeterminate SAI 6 (8%) and others 7 (9.3%) including 2 Blau syndrome, 2 Schnitzler syndrome, 1 DADA2, 1 SAVI and 1 DITRA.

The allelic variants found among the cohort were mainly monoallelic in 48 (64%) cases, biallelic in 2 (2.6%) cases, double monoallelic in 9 (12%) cases while no variant was found in 16 /21% cases (PFAPA, Schnitzler syndrome and indeterminate SAI patients).

Among the 75 patients, 10 (20.1%) presented any autoimmune systemic / organ-specific autoimmune disease: 2 spondyloarthritis, 2 autoimmune thyroiditis, and 1 case each of ANCA-associated vasculitis, autoimmune thrombopenia, rheumathoid arthritis, AIJ, urticaria-vasculitis and PLA2R-associated glomerulonephritis. The most prevalent associated SAI was FMF (7 , 70%).

Among the autoantibodies profile, 17 (22%) patients showed any autoantibody with 4 cases with >1 autoantiboy isolated. Antinuclear antibodies were detected in 8 patients at a titer ≥ 1/160. ANCA antibodies and rheumatoid factor were found in 3 cases each. Anti-slim muscle and anti-thyroid antibodies were found in 2 cases each while anti-Ro52, anti-PLA2R, anti-gastric parietal cell antibodies, anti-CCP and direct Coombs were identified in 1 case each. The most prevalent SAI among autoantibody carrier patients was also FMF (6 cases, 35%).

No autoantibody was found in 4 out of 10 patients with diagnosis of any autoimmune condition. On the opposite point of view, 6/17 patients with autoantibodies had an autoimmune disease.

**Conclusion:** Autoimmune diseases are not so unfrequent among adult patients diagnosed with SAI of our cohort, mainly in FMF patients, as expected for the most prevalent SAI.

Positivity for any autoantibody were present among >20% of the cohort patients but the presence of autoantibodies was linked to an autoimmune disease in only 1/3 of cases.

The coexistence on the same patient of autoimmune and autoinflammatory conditions is unfrequent but not mutually excluding. On the other hand, positivity for autoantibodies have proved in this cohort a limited value as a clue for the identification of an underlying autoimmune diesase.

**Disclosure of Interest**

None Declared

### P12 A new autoinflammatory variation in the human domain of NLRC4 gene associated with infantile enterocolitis

#### L. Martinez Mitjana^1^, M. Lopez Corbeto^1^, E. Moreno Ruzafa^1^, E. García-Arumí^2^, E. Tizziano^2^, M. Antolín^2^

##### ^1^Pediatric Rheumatology, Hospital Universitario Vall d'Hebron; ^2^Department of Clinical and Molecular Genetics, Hospital Universitario Vall d’Hebron, Barcelona, Spain

###### **Correspondence:** L. Martinez Mitjana

**Introduction:** NLR family CARD domain-containing protein 4 (NLRC4) is a key component of inflammasomes that indirectly senses specific proteins from pathogenic bacteria and fungi and responds by assembling an inflammasome complex that promotes caspase-1 activation, cytokine production and macrophage pyroptosis. Mutations in this gene have been reported as part of NLRC4-related familial cold autoinflammatory syndrome and periodic fever-infantile enterocolitis autoinflammatory syndrome. We report a case of a 15-year old male with an enterocolitis autoinflammatory syndrome phenotype with a not previously reported mutation in NRLC4.

**Objectives:** To establish a molecular diagnosis in a patient with unreported family history of neonatal-onset enterocolitis, periodic fever and arthritis, well controlled with Anakinra.

**Methods:** We report the case of a 15-year-old male patient who has been followed up in the pediatric rheumatology unit since the neonatal period. Born to non-consanguineous parents of Catalan origin, he had no family history of immune-mediated disorders. He required admission at 3 days old due to fever, salmon-coloured macular truncal rash and gastroenterological and neurological signs suggesting both enterocolitis and meningitis. At an analytical level, the elevation of acute phase reactants with ESR> 120 mm/h and CRP> 10 mg/dl stood out. All microbiological tests were negative. A Sanger study was requested for CIAS1 which was negative. Given the suspicion of autoinflammatory syndrome, without being able to identify the exact cause, treatment with corticosteroids was started with clinical improvement. At 2 years of age, he was admitted due to arthritis in both hips requiring new corticosteroid treatment that could not be withdrawn due to persistent local inflammation. Finally, at 11-years-old, treatment with Ankinra at 100 mg/day was started with good clinical control.

At 13-years-old, new DNA samples were obtained from the patient and his parents and the genetic autoinflammatory disease study was widen by capturing and sequencing the flanking exonic and intronic areas of the following 17 genes: *MEFV, MVK, TNFRSF1A, NOD2, NLRP3, NLRC4, NLRP12, IL1RN, IL6RN, PLCG2, PSTPIP1, CARD14, TMEM173, PSSMB8, LPIN2, CECR1* and *TNFAIP3.*

The study was conducted using the SeqCap EZ HyperCap (NimbleGen) methodology on a Illumina's MiSeq platform. Bioinformatic analysis was performed with the sequences obtained: alignment against the hg38 genome with BWA-MEM, detection of changes with GATK and annotation with ANNOVAR. Changes with a variant/reading ratio> 0.2 wereconsidered variants. Variants identified as pathogenic or probably pathogenic, as well as areas with coverage <20x, were studied by capillary sequencing. The nomenclature used is the one recommended by HGVS.

**Results:** The c.1015C>G p.(Leu339Val) missense variant was detected in heterozygosis in *NLRC4* NM_0212209.4, a gene intolerant to missense variation (z-score=0.977). Moreover, this variant which was not found in population database (gnomAD), was located in the NLRC4_HUMAN domain 'NACHT', where most of the missense variants were previously described as pathogenic, and a change in the same aminoacid,p.(Leu339Pro), has been recently reported in a symptomatic individual with an autoinflammation with infantile enterocolitis (AIFEC) phenotype. Additional studies in the progenitors of our patient demonstrated that it was a *de novo* variant. Taking into account all this findings and according to the classification proposed by the American College of Medical Genetics and Genomics (ACMG) the c.1015C>G p.(Leu339Val) missense variant should be considered as a likely pathogenic variant.

**Conclusion:** We describe a de novo mutation in *NLRC4* gene encoding a Leu339Val substitution, which confirms the clinical diagnosis of autoinflammation with infantile enterocolitis (AIFEC).

**Disclosure of Interest**

None Declared

### P13 Recurrent fever diagnosis: a case disguised as PFAPA syndrome

#### C. Naharro-Fernández^1^, N. Palmou-Fontana^2^, A. E. López- Lundh^1^, S. Armesto Alonso^1^, B. Jimenez-Montero^3^, C. Alvarez-Alvarez^3^, M. J. Cabero-Perez^3^

##### ^1^Dermatology; ^2^Rheumatology, Universitary Hospital Marques De Valdecilla; ^3^Paediatrician, Hospital Universitario Marquez De Valdecilla, Santander, Spain

###### **Correspondence:** C. Naharro-Fernández

**Introduction:** Fever is a very common symptom in childhood. In the first years of life, a healthy child could have annually up to twelve fever episodes, especially during the cold season. In most cases, these episodes correspond to viral infections. However, in other circumstances, infectious etiology is not responsible,and diagnosis becomes a challenge for the pediatrician.

Differential diagnosis of recurrent feverencompassesneoplastic processes, autoinflammatory diseases and immunodeficiencies.

**Keywords:** Recurrent fever; Aphthae; Aphthous stomatitis PFAPA syndrome; Familiar Mediterranean Fever; FMF; MEVF

**Abbreviations:** FMF: Familiar Mediterranean Fever; MEVF: Familiar Mediterranean Fever Gene; PFAPA: Periodic Fever; Aphthous Stomatitis; Pharyngitis; Adenitis

**Objectives:** Recurrent fever is common in childhood. Differential diagnosis should be conducted in some patients. Autoinflammatory diseases could be responsible in some circumstances but diagnosis is not easy, as different syndromes could appear overlapped. We present a clinical case of a boy with recurrent fever, aphthous stomatitisand growing delay.

**Methods:** We present the case of a 10-year-old boy with recurrent fever and aphthous stomatitis.

He was asthmatic and he received treatment with inhaled corticosteroids. The child had short stature with a delay in bone age of nearly three years. Parents referred previous episodes of fever andaphthae.

At the age of nine, he was admitted at the hospital with fever, erosive lesion in mouth

and decreased intake that started three days before.

Physical examination highlighted multiple aphthae on oral mucosa, hard palate and soft palate and cervical bilateral lymphadenopathies.

Blood count revealed leukocytosis and RCP of 2,9 mg/dl with a maximum during the hospital admission of 8,3 mg/dl.Chest radiograph showed a left lower lobe infiltrate suggesting pneumonia. He received amoxicillin-clavulanic acid and azithromycin.After 72 hours fever disappeared and oral lesion improved.

Serology for herpes simplex virus and bacteria were negative. *Mycoplasma pneumoniae* IgM and IgG were positives.

The patient was discharged with diagnosis of mucositis cause by *Mycoplasma pneumoniae*.

**Results:** The patient continued to present additional episodes of fever and oral lesions. A rheumatology study was conducted, showing negative immunodeficiency and autoimmunity. Despite the fact that the patient fulfilled the diagnosis criteria for PFAPA, a genetic study was requested and the potentially pathogenic mutation p.Lys695Arg was detected in the gene MEFV.

We finally made the diagnosis of FMF in our case. Colchicina was prescribed and the boy became asymptomatic in a few weeks.

**Conclusion:**

However, our genetical study revealed a heterozygous MEFV mutations affecting to exon 10 showing that Familial Mediterranean fever should be considered in differential diagnosis of PFAPA. In some patients, genetic screening could help to make diagnosis of autoinflammatory disease with a PFAPA-like presentation. In this hypothesis underlying MEFV gene mutations possibly lead to PFAPA-like clinical presentation in FMF patients.

Advances in genetic testing enable to identify genetic diseases with higher sensibility. Clinical presentation of autoinflammatory syndromes are often overlapped, especially in PFAFA syndrome, where an important rate of mutation in MEFV gene has been detected. We should not forget that FMF could have a PFAPA-like presentation in order not to misdiagnose this entity. Genetic test in selected patients could allow us to make the correct diagnosis in order to improve prognosis of our patients and families.

Advances in genetic testing enable to identify genetic diseases with higher sensibility. Clinical presentation of autoinflammatory syndromes are often overlapped, especially in PFAFA syndrome, where an important rate of mutation in MEFV gene has been detected. We should not forget that FMF could have a PFAPA-like presentation in order not to misdiagnose this entity. Genetic test in selected patients could allow us to make the correct diagnosis in order to improve prognosis of our patients and families.

**Disclosure of Interest**

None Declared

### P14 A patient diagnosed with sifd syndrome (Sideroblastic anemia with immunodeficiency, fevers, and developmental delay)

#### A. Paç Kisaarslan, S. Ozdemir Cicek, N. Şahin, H. Poyrazoglu

##### Pediatric Rheumatology, Erciyes University School of Medicine, Kayseri, Turkey

###### **Correspondence:** A. Paç Kisaarslan

**Introduction:** Immunodeficiencies with autoinflammatory diseases are increasingly being defined.Clinical findings are described wide range of system. It takes time to diagnose these syndromes. Fever and high acute phase reactants that cannot be explained by infection in immunocompromised patients should suggest autoinflammatory diseases.

**Objectives:** Here we present a patient with SIFD syndrome (Sideroblastic anemia with immunodeficiency, fevers, and developmental delay) with hypogammaglobulinemia, anemia, growth retardation, developmental delay and autoinflammatory findings.

**Methods:** Twenty Seven-month-old girl suffered from diarrhea and fever with high acute phase reactants for 4-month-old. Hypogammaglobulinemia and microcytic anemia were detected at 7-month-old. Intravenous immunoglobulin (IVIG) treatment was administered, but fever and diarrhea attacks were not resolved. Her colonoscopic and biopsy findings were normal. She had growth and developmental retardations at 18-month-old. Her attacks were resolved by steroid treatment. But, IL-1 blockade (anakinra) did not effect the attacks. Her autoinflammatory panel did not show any mutations. Parents denied a further investigation. The patient was administered monthly IVIG, and low dose steroid treatment for one year. The patient was admitted to our rheumatology department at 27-mounth old. On the physical examination, height SDS:-5.26, weight SDS: -5,27 , mental and motor retardation, microcytic anemia( not sideroblastic anemia) were detected. Her whole exon sequencing (WES) showed g.3188201_3188201 del NM_182916.2: c.679delA p.(I233fs*10) on exon 6, and K416E on exon 8 heterozygous mutations. After etanercept treatment, fever and diarrhea attacks resolved, she gained weight and started sitting.

**Results:** Sideroblastic anemia with immunodeficiency, fevers, and developmental delay (SIFD syndrome:OMIM 616084) is developed due to mutation of the tRNA nucleotidyltransferase 1 gene. The syndrome is characterized by fever, microcytic or sideroblastic anemia, B cell deficiency, HLH, growth and developmental delay, cerebral atrophy, deafness and blindness, arthritis, panniculitis, oral ulcers, subcutaneous nodules. IL-1 and TNF blockade, IVIG, and HSCT are recommended treatment of SIFD syndrome.

**Conclusion:** Immune deficiencies with autoinflammatory findings are defined increasing rate. Genetic analysis is a significant diagnostic methot of these diseases. The importance of WES analysis increases in autoinflammatory patients who cannot be diagnosed, especially in the presence of accompanying signs of immunodeficiency.

**Disclosure of Interest**

None Declared

### P15 Safety of anakinra in patients with Cryopyrin Associated Periodic Syndromes (CAPS) using a graduated pre-filled syringe

This abstract has not been included here as it has been previously published

### P16 A patient with candle-like phenotype and de novo frameshift mutation in the SAMD9L gene

#### A. Remy^1,2^, C. Borocco^3,4^, G. Sarrabay^5^, G. Boursier^5^, S. Fraitag^6^, B. Catteau^2,7^, H. Reumaux^2,8^, I. Koné-Paut ^3,9^

##### ^1^Pediatric Emergency, Infectious Diseases and Rheumatology Department, CHU Lille; ^2^Faculté de médecine, University of Lille, Lille; ^3^Pediatric Rheumatology Department, CHU Bicêtre, AP-HP, Le Kremlin-Bicêtre; ^4^University of Paris Saclay, Paris; ^5^Laboratory of Rare and Autoinflammatory Genetic Diseases, CHU Montpellier, Montpellier; ^6^Pathology Department, CHU Necker-Enfants Malades, AP-HP, Paris; ^7^Dermatology Department; ^8^CHU Lille, Lille; ^9^Faculté de médecine, University of Paris Saclay, Paris, France

###### **Correspondence:** A. Remy

**Introduction:** Chronic Atypical Neutrophilic Dermatosis with Lipodystrophy and Elevated Temperature syndrome (CANDLE) syndrome belongs to a group of rare monogenic systemic autoinflammatory diseases (SAID) also called interferonopathies. They originate in aberrant IFN production and signaling. Prototypic CANDLE syndrome is linked to recessive mutations in immunoproteasome subunits genes, e.g. subunit beta type 8 (PSMB8), affecting the clearance of damaged proteins via the proteasome (1).

**Objectives:** To underline the interest of pursuing the investigations when both the phenotype and the genotype of a patient does not come strictly within the framework of what has been previously known.

**Methods:** We report a case of a child with prominent inflammatory and cutaneous phenotype that appeared at birth. Despite initial next generation sequencing (NGS) of a small panel of autoinflammatory genes showed an heterozygous mutation in *PSMB8* gene, the patient did not present typical features of CANDLE. Further genetical investigations rescued diagnosis.

**Results:** The girl was born to unrelated Caucasian parents. From birth, she presented with urticaria that covered her face and limbs, became ecchymotic, and extended to palms and soles. Since the age of 3 months old, she developed unexplained recurrent fever (2 days long, twice a month) and painful edema of extremities without true arthritis. Blood work-up showed continuous and moderate inflammation during and in-between outbreaks. A diagnostic of SAID was suggested. The patient received anakinra with partial efficacy. Skin biopsy showed inflammatory infiltrate in the whole dermis that was compatible with the diagnosis of CANDLE. NGS of an auto-inflammatory genes panel did not find any variant with a putative pathogenic role notably in the *NLRP3*-associated autoinflammatory syndrome and mevalonate kinase deficiency. However, it showed one heterozygous p.(Thr74Ser) variant in the *PMSB8* gene (2). Despite the fact that our patient had prominent inflammatory and cutaneous phenotype, the constellation of criteria was incomplete and atypical for CANDLE syndrome. First, her skin examination by an experienced dermatologist did not show lipodystrophy. Then, other marked differences were the absence of hepatomegaly, of cytopenia, and the mildly elevated interferon signature. There was more than met the eyes. Genetic analysis was pursued with whole exome sequencing revealing a *de novo* frameshift variant p.(Ile876Leufs*15) in the Sterile Alpha Motif Domain–containing protein 9-Like (*SAMD9L)* gene. Treatment switch with JAK inhibitor (baricitinib) finally enabled a clear skin improvement.

In a recent study, *SAMD9L* mutations were identified in six patients among twelve with CANDLE-like phenotype (3). The overall phenotype of patients is very severe as two out of six patients are deceased at an early age and two other patients underwent bone marrow transplantation for severe cytopenia or interstitial lung disease. Our patient (and seemingly the previously described patient carrying the c.2626del/p.(Ile876Leufs*15) mutation) had milder phenotype than did the other five. So far, at 7 years old, she does not present more than a systemic autoinflammatory phenotype with panniculitis.

**Conclusion:** SAMD9L associated autoinflammatory disease is a new entity whose clinical feature overlaps with CANDLE. It is worth to be separated from CANDLE as it may predispose to early and severe complications that need to be prevented. Indeed, early and severe ILD or cytopenia may justify eligibility to hematopoietic stem cell transplantation. As a treatment concern, patients with SAAD may respond to JAK inhibiting therapies despite their interferon signature may be only moderately elevated(4). The functional consequences of *SAMD9L* gene mutations need to be clarified to optimize targeted therapy.

(1) Torrelo A. CANDLE Syndrome As a Paradigm of Proteasome-Related Autoinflammation. Front Immunol 2017;8:927.

(2) Insalaco A, Prencipe G, Pardeo M, et al. Involvement of the IFN-gamma pathway in a patient with candle syndrome carrying a novel variant of PSMB8 gene. Pediatr Rheumatol Online J 2014;12:P249.

(3) de Jesus AA, Hou Y, Brooks S, et al. Distinct interferon signatures and cytokine patterns define additional systemic autoinflammatory diseases. J Clin Invest 2020;130:1669–82.

(4) Sanchez GAM, Reinhardt A, Ramsey S, et al. JAK1/2 inhibition with baricitinib in the treatment of autoinflammatory interferonopathies. J Clin Invest 2018;128:3041–52.

**Disclosure of Interest**

None Declared

### P17 Antiinflammatory, antioxidant and anti-atherosclerotic effects of a combination of natural supplements on patients with FMF related AA amyloidosis: a non-randomized 24 weeks open label interventional study

This abstract has not been included here as it has been previously published

### P18 The effect of M694V homozygosity on the carotid intima-media thickness and flow mediated dilatation in patients with fmf related amyloidosis

This abstract has not been included here as it has been previously published

### P19 From mild to severe distinct faces of adenosine deaminase 2 deficiency in the single pediatric rheumatology center study

#### B. Sozeri, F. Demir, S. Caglayan, K. Ulu, T. Coskuner

##### Pediatric Rheumatology, University of Health Sciences , Istanbul, Umraniye Training and Research Hospital,, Istanbul, Turkey

###### **Correspondence:** B. Sozeri

**Introduction:** Deficiency of adenosine deaminase 2 (DADA2) is an autosomal recessive disorder characterized by poliarteritis nodosa-like symptoms, fever, livedoid racemose, early-onset stroke and mild immunodeficiency. The loss-of-function mutations in CECR1 gene which encodes the enzymatic protein adenosine deaminase 2 (ADA2) associated with the DADA2.

DADA2 is present with a spectrum of vascular and inflammatory phenotypes with highly varied clinical expression. The patients can present with isolated constitutional symptoms, arthralgia, myalgia, and/or livedoid rash. The severe phenotype of the disease is emerge as early-onset stroke, bone morrow suppression, neutropenia, liver failure and/or neurologic disorders It is currently not well understood what cause the variability in the severity of clinical phenotype.

**Objectives:** We aim to describe the clinical characteristics, genotype and treatment of patients diagnosed with DADA2 in our clinic.

**Methods:** All the patients diagnosed and followed up in Department of Pediatric Rheumatology, Umraniye Training and Research Hospital, Istanbul, Turkey, between 2016 and 2020. All symptomatic patients suspected with DADA2 were analyze by genetic test and ADA-2 enzyme activity, also relatives of index cases were also screened. ADA-2 enzyme activity was evaluated at Duke University Medical Center.

**Results:** We diagnosed 10 patients with DADA2, 5 of them had mild phenotype or asymptomatic, or they were relatives of index cases, and 5 of the patients had severe phenotype. Three of the patients were girls and seven were boysThe age of the patients at diagnosis was in the range of 2–28 years. The age of the patients at disease onset was also in the range of 2 months–14 years. Six of the patients were index cases and the other four were their relatives. Homozygous mutations in CECR1 were shown in all subjects.The frequency of phenotypic manifestations of the patients were seen as follow respectively; fever 80%, livedoid rash 70%, digital ulsers and/or amputations 20%, neurologic involvement 50%, gastrointestinal involvement 20%, hematological involvement 40%, and immunodeficiency 30%.The initial diagnosis of index cases were as follows; two classic PAN, one cutaneos PAN, one Behçet’s disease and two Diamond Blackfan syndrome. The activity of ADA2 was evaluated and clearly diminished in all patients (0-12.1 mU/g protein). 6 of the 10 patients were treated with anti-tumor necrosis factor therapy.Two patients were awaiting bone marrow transplantation.

**Conclusion:** The clinical manifestations of DADA2 vary from mild skin involvement to severe multisystemic vasculitis. It is suggesting that both residual ADA2 activity levels and gene mutation are important in clinical phenotype.

**Disclosure of Interest**

None Declared

### P20 Real-life indications of interleukin-1 blocking agents in hereditary recurrent fevers: data from the jircohort and a literature review

#### C. Vinit^1^, S. Georgin-Lavialle^2^, A. Theodoropoulou^3^, S. Atmane^4^, C. Barbier^5^, A. Belot^6^, F. Hofer^3^, M. Mejbri^3^, P. Pillet^7^, J. Pachlopnik^8^, S. Poignant^9^, C. Rebelle^7^, A. Woerner^10^, I. Kone-Paut^4^, V. Hentgen^1^

##### ^1^Versailles hospital, Versailles; ^2^Tenon hospital, Paris, France; ^3^Lausanne University Hospital, Geneva, Switzerland; ^4^Bicetre University hospital, Kremlin Bicetre, ^5^Grenoble Hospital, Grenoble; ^6^Lyon University hospital, Lyon; ^7^Bordeaux University hospital, Bordeaux, France; ^8^Kinderspital, Zurich, Switzerland; ^9^Nantes University hospital, Nantes, France; ^10^UKBB Hospital, Bâle, Switzerland

###### **Correspondence:** C. Vinit

**Introduction:** Interleukin (IL)-1 inhibitors represent the main treatment in patients with familial Mediterranean fever (and colchicine resistance or intolerance, crFMF), mevalonate kinase deficiency (MKD) and periodic tumor necrosis factor receptor-related syndrome (TRAPS). However, the reasons for use of IL-1 inhibitors in these diseases are still unclear.

**Objectives:** Identify real life situations that led to use anakinra or canakinumab in hereditary recurrent fevers (HRF), combining data from an international registry, the JIR cohort, and an up-to-date literature review.

**Methods:** We extracted data from a web-based registry: the JIRcohort, in which clinical information (demographic data, treatment, disease activity and quality of life) on patients with FMF, MKD and TRAPS was collected retrospectively. A literature search was conducted using Medline and EMBASE.

**Results:** We analyzed complete data of 93 patients with HRF (53.8% FMF, 31.2% MKD and 15.1% TRAPS). Data from both the registry and the literature review confirmed that the main reasons for use of IL-1 blockers were: failure of previous treatment (n=57, 61.3% and n=964, 75.3% respectively), severe disease complication or associated comorbidities (n=38, 40.9% and n=390, 30.4%), persistence of disease activity frequent attacks (n=44, 47.3% and n=1,023, 79.9%) and/or uncontrolled inflammatory syndrome (n=46, 49.5% and n=398, 31.1%), and worsening in patients' quality of life (n=36, 38.7% and n=100, 7,8%). No reasons were specified for 12 (16.4%) JIR cohort patients and 154 (12%) patients in the literature.

**Conclusion:** In the absence of standardized indications for IL1 inhibitors in crFMF, MKD and TRAPS, these results could serve as a basis for developing a standardized composite score that would help clinicians to assess the activity and severity of HRF at a specific time point in order to evaluate the need of a therapeutic escalation with IL-1 inhibitors.

**Acknowledgments**

keywords : hereditary recurrent fevers, indications, interleukin-1 blocking agents, anakinra, canakinumab

**Disclosure of Interest**

None Declared

### P21 Dysregulation of MIR-505-5P which targets CYP3A4 in colchicine-resistant familial mediterranean fever patients

#### T. H. H. Akbaba^1^, B. S. Ozdemir^1^, Y. Z. Akkaya-Ulum^1^, P. Mutlu^2^, S. Ozen^3^, B. Balci-Peynircioglu^1^

##### ^1^Medical Biology, Hacettepe University, ^2^Middle East Technical University, ^3^Hacettepe University, Ankara, Turkey

###### **Correspondence:** T. H. H. Akbaba

**Introduction:** Familial Mediterranean Fever (FMF) is an autosomal recessive inherited autoinflammatory disease. The well-known treatment of the disease is daily colchicine uptake. However, approximately 5% of patients do not respond to colchicine treatment despite using the highest tolerable colchicine. In addition to colchicine treatment, these patients receive anakinra, canakinumab, and similar drugs to prevent inflammation.

**Objectives:** This study aims to investigate the potential impact of miRNAs on drug resistance observed in FMF patients.

**Methods:** Microarray data obtained in our previous studies was re-evaluated for colchicine-resistant patients with severe phenotype and candidate miRNAs that are involved in drug metabolism were determined by performing bioinformatics analysis. miRNA-target gene studies were performed and miRNAs which have a role in the mechanism of drug resistance in FMF were determined. Then, 3'UTR luciferase activity experiments were carried out to determine the target gene. Then, target gene expression studies were performed.

**Results:** As a result of miRNA array analysis, miR-186-3p, miR-548a-3p, miR-7-5p were decreased, miR-505-5p and miR-4482-3p were increased in colchicine-resistant familial Mediterranean fever patients. miR-505-5p was found as a regulator of drug metabolism and drug resistance-related genes by bioinformatical tools and validated by qRT-PCR. Target gene studies performed in HEPG2 cell (hepatocellular carcinoma cell line) showed that miR-505-5p regulates CYP3A4 expression at the RNA level. MiR-505-5p – CYP3A4 interaction was shown by 3’UTR luciferase assay.

**Conclusion:** Functional analyzes related to colchicine metabolism are underway. A possibly explanation about drug resistance observed in complex FMF patients through miRNAs will allow the development of new drug targets for these patients in the future.

**Acknowledgments**

This project has been funded by The Technical and Scientific Research Council of Turkey, 218S522.

**Disclosure of Interest**

None Declared

### P22 An atypical periodic fever syndrome due to a mutation in the leucine rich repeat domain of NLRP3

#### E. A. Caseley^1^, J. Topping^1^, S. Savic^1,2,3^, M. F. McDermott^1^

##### ^1^Leeds Institute of Rheumatic and Musculoskeletal Medicine, St James's University Hospital; ^2^Department of Clinical Immunology and Allergy, St James’s University Hospital; ^3^National Institute for Health Research–Leeds Biomedical Research Centre, Chapel Allerton Hospital, Leeds, United Kingdom

###### **Correspondence:** E. A. Caseley

**Introduction:** Gain-of-function mutations in the NLRP3 inflammasome cause *NLRP3*-associated autoinflammatory diseases (*NLRP3*-AIDs); these mutations are rarely located in the leucine rich repeat (LRR) domain. Here we present a patient with a c.2753 G>A variant in *NLRP3* exon 7, resulting in the Arg918Gln (R918Q) mutation in the LRR domain of NLRP3(1), causing oral ulcers and hearing loss; she was significantly less responsive to anakinra treatment compared to previously reported cases of this mutation but has responded well to apremilast, a phosphodiesterase 4 inhibitor.

**Objectives:** We aimed to identify molecular mechanisms contributing to this patient’s unusual disease presentation and response to treatment, in addition to characterising changes in protein-protein interactions caused by the R918Q mutation.

**Methods:** We isolated CD14+ monocytes from the patient or three healthy controls (HCs) and analysed cell responses after NLRP3 stimulation using RT-PCR, ELISA or flow cytometry. Monocytes were also differentiated into M0, M1 or M2 macrophages, from which RNA was isolated and used for RNA sequencing (RNA-seq). Interactions between NLRP3 and NIMA-related kinase 7 (NEK7) were analysed *in silico* using PDBePISA and PyMOL, combined with immunoprecipitation and subsequent immunoblotting studies in HEK293T cells transfected with NLRP3 and NEK7.

**Results:** Elevated IL-1β release was seen in patient’s monocytes when unstimulated or stimulated with a priming stimulus alone compared to the HCs, a hallmark of *NLRP3*-AIDs. This was accompanied by a significant increase in IL-18 release in response to NLRP3 stimulation in the patient’s cells and elevated IL-6 in patient serum. Extracellular ASC specks, pivotal components of the NLRP3 inflammasome, were also more readily released by the patient’s monocytes. Transcriptomic profiling of NLRP3-R918Q macrophages showed similar M1 and M2 macrophage genotypes in patient’s cells compared to HCs, suggesting impaired mucosal tissue healing by M2 macrophages, which may contribute to the patient’s persistent oral ulcers. KEGG enrichment analysis further showed significant enrichment of inflammatory pathways, particularly the IL-17 and NF-kB signalling pathways. Upregulation of IL-17 indicates a skewing of T-helper responses towards a Th17 phenotype, as previously observed in *NLRP3*-AIDs(2), in addition to IL17A and NF-kB being a biomarker for apremilast efficacy(3), partly explaining this patient’s unusual response to therapy. Molecular characterisation of the R918Q mutation was also carried out, using the recently determined cryo-electron microscopy structure of NLRP3 in complex with its endogenous regulator, NEK7(4). The R918Q mutation was shown *in silico* to increase the electrostatic complementarity between the NLRP3 LRR domain and the interacting interface of NEK7. This was confirmed by co-immunoprecipitation experiments; cells transfected with the mutant NLRP3-R918Q showed increased binding to NEK7 compared to WT NLRP3.

**Conclusion:** Here we show that the *NLRP3*-R918Q mutation causes an atypical periodic fever syndrome with significant mucosal involvement. The involvement of factors such as a Th17 phenotype, in addition to increased NLRP3 inflammasome activity, go some way to explaining the lack of efficacy of anakinra treatment. We propose that the R918Q mutation enhances NLRP3 inflammasome activity by increasing interactions between the NLRP3 and NEK7 proteins, thereby lowering the threshold required for NLRP3 inflammasome activation.

**References**

1. Nakanishi H, Kawashima Y, Kurima K, Muskett JA, Kim HJ, Brewer CC, et al. Gradual symmetric progression of DFNA34 hearing loss caused by an NLRP3 mutation and cochlear autoinflammation. Otology & neurotology: official publication of the American Otological Society, American Neurotology Society [and] European Academy of Otology and Neurotology. 2018;39(3):e181.

2. Meng G, Zhang F, Fuss I, Kitani A, Strober W. A mutation in the Nlrp3 gene causing inflammasome hyperactivation potentiates Th17 cell-dominant immune responses. Immunity. 2009;30(6):860-74.

3. Medvedeva IV, Stokes ME, Eisinger D, LaBrie ST, Ai J, Trotter MW, et al. Large-scale analyses of disease biomarkers and apremilast pharmacodynamic effects. Scientific reports. 2020;10(1):1-11.

4. He Y, Zeng MY, Yang D, Motro B, Núñez G. NEK7 is an essential mediator of NLRP3 activation downstream of potassium efflux. Nature. 2016;530(7590):354-7.

**Acknowledgments**

This project has received funding from the European Union’s Horizon 2020 research and innovation programme under the grant agreement No: 779295

**Disclosure of Interest**

None Declared

### P23 SAA1 Polymorphisms contribute more than mefv mutations in renal AA-amyloidosis -preliminary results

#### D. Ait-Idir^1^, B. Djerdjouri^2^

##### ^1^Biology, Faculty of sciences, University M'hamed Bougara, Boumerdes; ^2^Faculty of Biological Sciences, University of Sciences and Technology Houari Boumediene, Algiers, Algeria

###### **Correspondence:** D. Ait-Idir

**Introduction:** Renal AA amyloidosis can develop with a high prevalence in untreated patients with familial Mediterranean fever (FMF, OMIM 249100). The MEFV and SAA1 genotypes are the two genetic risk factors that have been associated with the development of this serious complication.

**Objectives:** This study aimed to evaluate the involvement of each genetic factor in the occurrence of renal AAamyloidosis.

**Methods:** Sixty-two unrelated Algerian FMF patients were included in this study. Among them, 41 (19 men, 22 women; mean age: 36.26 ± 14.43 years) with biopsy-confirmed AA amyloidosis were recruited from the nephrology departments, and 21 (9 men, 12 women; 33, 80 ± 14, 71 years) without renal complications came from the internal medicine departments.

Sixty-two unrelated Algerian FMF patients were included in this study. Among them, 41 (19 men, 22 women;mean age: 36.26 ± 14.43 years) with biopsy-confirmed AA amyloidosis were recruited from the nephrology departments,and 21 (9 men, 12 women; 33, 80 ± 14, 71 years) without renal complications came from the internal medicinedepartments.

**Results:** Screening of the *MEFV* gene revealed a significant correlation between the M694I/M694I genotype and renal amyloidosis. The genotypes of the *SAA1* locus were differentially distributed between the two groups of patients. A significant predominance of SAA1.1/1.1 genotype was found in patients with amyloidosis, compared to those without this complication (p=0.001), while the SAA1.5/1.5 genotype was exclusively associated with patients without amyloidosis. Logistic regression analysis for the risk of development of amyloidosis revealed a 4.3times increase in risk in the M694I/M694I genotype (95% CI 1,35 – 14). However, this risk increases eight times in presence of the SAA1.1/1.1 genotype (OR 8.7; 95% CI 2.25 – 33).

**Conclusion:** Our preliminary results based on a small cohort of FMF patients suggest that genotypes at the SAA1 locus may predispose more than genotypes at the MEFV locus to the occurrence of renal AA amyloidosis.

**Acknowledgments**

We thank the physicians for their contribution in patients recruitment.

**Disclosure of Interest**

None Declared

### P24 Is interferonopathy caused nasal perforation in a patient with nijmegen breakage syndrome ?

#### S. S. Kilic

##### Pediatric Rheumatology, Uludag University Medical Faculty, Bursa, Turkey

**Introduction:** Nijmegen breakage syndrome (NBS) is a rare autosomal recessive disorder of chromosomal instability. It is characterized by short stature, microcephaly, a typical facial appearance, immunodeficiency, radiation sensitivity, and a strong predisposition to lymphoid malignancy. NBS is caused by a mutation in the *NBS1* gene, located at human chromosome 8q21.

**Objectives:** We present a case with NBS suspected having interferonopathy

**Methods:** A 14 years old girl was first admitted to our outpatient clinic with the complaint of perforated nasal septum, chronic otitis externa and persistent skin lesions. There was a parental consanguinity. Nasal septum perforation occurred at 10 years of age. Physical examination revealed; weight: 38 kg (<3P), height: 136 cm (<3P), microcephaly, crepitation in both lung bases, ulcerated lesion below the right eye and from right upper lip extending to the nasal septum which was perforated. While total blood count, erythrocyte sedimentation rate, IgG, IgG1, IgG3, IgA, IgE, NBT were normal, serum IgM <16,9 mg/dl (88-322 mg/dl) and IgG2: 183 mg/dl (236-605 mg/dl) levels were low. Lymphocyte subsets were normal except for slight low CD8 (16.3%, 489/mm^3^). Antibody responses (anti HBS and tetanus IgG) were negative. HLA ABC expression by flow cytometry was 99 % (MFI:13669). Histologic analysis of a nasal septum biopsy was unhelpful, revealing only a nonspecific polymorphic inflammatory infiltrate without perivascular distribution and an absence of vasculitis or granulomatosis. PPD was15x17mm, quantiferon measurement test and sputum culture were negative. Tests for leishmaniasis ere negative. Thorax CT showed bronchiectasis in the middle lobe of right lung. A homozygous JL2494 – *NBN* exon 3 mutation was found by kindly Anne Puel's lab. Nijmegen breakage syndrome was diagnosed. The patient commenced on IVIG treatment. Stem cell transplantation has been planned.

**Conclusion:** It has been showed that type I and III IFN signatures were elevated in patients with AT, Artemis deficiency, and SAVI. Although cutaneous granulomas have been reported in patients with NBS but interferonopathy has not been reported. We believe that, more information is needed on this issue.

**Disclosure of Interest**

None Declared

### P25 Patient Engaged Genomic Understanding and Interpretation (PENGUIN): a case study in patients with Familial Mediterranean Fever (FMF)

#### K. A. Maloney^1^, M. Giglio^1^, H. Zhang^1^, L. Jeng^1^, D. Sewell^2^, N. Ambulos^3^, A. Beitelshees^1^, E. Streeten^1^, D. Loesch^1^, N. Sampaio Moura^1^, N. Dueker^4^, K. Palmer^1^, R. Gore^5^, S. Kittner^6^, T. O'Connor^1^, J. Jagger^7^, T. Pollin^1^, S. Riley^5^

##### ^1^Medicine; ^2^Translational Genomics Laboratory; ^3^Microbiology and Immunology, University of Maryland School of Medicine, Baltimore; ^4^University of Miami Miller School of Medicine, Miami; ^5^Pediatrics; ^6^Neurology, University of Maryland School of Medicine, Baltimore; ^7^Familial Mediterranean Fever Foundation, Barboursville, United States

###### **Correspondence:** K. A. Maloney

**Introduction:** Many patients with FMF are motivated to seek genomic information not available through their medical providers. We explored a novel solution providing patients with direct access to their genomic data while mitigating adverse consequences of misinformation arising from some direct-to-consumer genetic testing.

**Objectives:** To meet patients’ objectives for informed access to genomic data by providing them with their own exome sequence in the setting of a professionally administered genomics educational workshop.

**Methods:** Thirty-two adults, residing in the U.S. or Canada were recruited via email from the FMF Foundation. All provided medical documentation of clinical and/or molecular diagnosis of FMF. Following informed consent, saliva samples underwent clinical exome sequencing and data were filtered to analyze variants in 30 auto-inflammatory (AI) genes and 59 clinically significant genes (“secondary findings”) deemed actionable by the American College of Medical Genetics and Genomics (ACMG). Participants were informed of pathogenic and likely pathogenic variants in AI and ACMG genes, and variants of unknown significance (VUS) in AI genes. They also received their raw exome data files. An educational workshop featured eight sessions on general genetics, personal data exploration using GenomeBrowse, pharmacogenomics, and AI diseases. Participants completed surveys before and two weeks after the workshop (e.g., FMF disease severity, health numeracy, genomics knowledge, health literacy, self-efficacy).

**Results:** The PENGUIN workshop was held virtually on November 20-21, 2020, utilizing Zoom and Slack. Participants’ ages were from 24-74, 63% were female, and 84% had university degrees. They had high numeracy and genomic literacy. Twenty-two (69%) participants had prior *MEFV* genetic testing, including one who had research-based exome sequencing. PENGUIN testing detected all previously reported disease-associated *MEFV* variants; however, three variants were reclassified as VUS. Molecular diagnosis was newly confirmed in six individuals with a prior clinical diagnosis of FMF. Eighteen participants harbored 28 VUS in 14 AI genes; some of these may be risk variants as part of a complex etiology. Only VUS were found in five participants. Two individuals had secondary findings. Immediate feedback from the workshop was extremely positive, with participants expressing strong appreciation for the opportunity to receive comprehensive genetic results, understand and explore their genomic data, and interact with FMF patients, scientists and clinicians in a collegial manner.

**Conclusion:** The genetic findings across the AI spectrum confirmed FMF patients’ perceptions of the paucity of genetic information through their medical providers, validating their incentive to seek genetic testing outside of the medical system. As the cost of genomic sequencing decreases, the PENGUIN model of patient engagement may be a valuable method for meeting FMF and other rare genetic disease patients’ legitimate quest for access to comprehensive genomic data and knowledge. This inaugural group of FMF patients was engaged and capable, and future studies will need to assess the wider uptake and success of such a program.

**Acknowledgments**

We would like to thank the participants of the PENGUIN workshop and funding from the University of Maryland Dean's Challenge Grant.

**Disclosure of Interest**

None Declared

### P26 An atypical presentation of NLRP3-associated autoinflammatory disease

#### L. Marois^1^, S. Ducharme-Bénard^2^, H. Chapdelaine^3^

##### ^1^Institut de recherches cliniques de Montréal, ^2^Internal Medicine, Hopital du Sacré-Coeur de Montréal, ^3^Immunologie, Institut de recherches cliniques de Montréal, Montréal, Canada

###### **Correspondence:** L. Marois

**Introduction:** *NLRP3*-associated autoinflammatory diseases (NLRP3-AID) are rare genetic autoinflammatory diseases associated with gain-of-function mutations in *NLRP3* coding for cryopyrin. We hereby report a case with unique manifestations of NLRP3-AID.

**Results:** A 36-year-old French Canadian male presented with a 3-week history of left knee arthritis and systemic inflammation (neutrophilia, microcytic anemia, thrombocytosis, elevated C-reactive protein (208 mg/L), and erythrocyte sedimentation (94 mm/h). Joint aspiration revealed 161 500 white blood cells/ml with 91% neutrophils, low glucose level, elevated lactate, and negative gram stain. Despite treatment with cefazolin then meropenem and repeated arthroscopic joint drainages for suspicion of septic arthritis, no improvement was noted after one week. Synovial fluid and blood cultures returned negatives. At that point, an underlying inflammatory disorder was suspected. Antibiotics were discontinued and oral prednisone was introduced, with prompt improvement in inflammatory markers and knee swelling. Work-up for autoimmune diseases revealed negative anti-nuclear antibodies and anti-cyclic citrullinated peptide antibodies, normal complement levels, positive rheumatoid factor, and HLA B27 antigen. Screening for viral hepatitis, human immunodeficiency virus, tuberculosis, streptococcal infection, and Lyme disease was negative. Radiographs of axial and peripheral joints were unremarkable, apart from a right knee effusion that also responded to steroid therapy. One week later, the patient complained of chest pain, with a concomitant increase in inflammatory markers. Imaging revealed a right loculated pleural effusion. Pleural liquid revealed low pH, elevated proteins, elevated LDH, and undetectable glucose level, despite a surprisingly low white blood cell count and negative gram stain. Antibiotics were resumed for suspected complicated parapneumonic effusion, and the dose of prednisone was halved. Pleural bacterial, fungal, and mycobacterial cultures eventually came back negative. Antibiotics were nevertheless continued for a total of 4 weeks. Prednisone was also continued at 20 mg daily. Upon discharge, knee synovitis and pleural effusion had resolved, and C-reactive protein had decreased to 105 mg/L.

Family history was unremarkable, with no consanguinity. On the other hand, the patient had a personal history of aseptic meningitis at 2 years, sensorineural deafness, seronegative juvenile idiopathic arthritis (history of recurrent fevers with flares of arthritis and high inflammatory markers), pericarditis requiring drainage at 6 years, as well as a global developmental delay whose investigation by cerebral CT scan revealed diffuse atrophy and pseudopapilloedema. Also, the investigation of failure to thrive has demonstrated eosinophilic enteropathy.

In light of the long-standing history of sensorineural hearing loss, physical and mental developmental delay, intermittent serositis, and elevated inflammatory markers, NLRP3-AID was suspected. DNA analysis by next-generation sequencing (NGS) revealed a heterozygous mutation in exon 3 encoding the NACHT domain of the *NLRP3* gene, c.1991T>C (p.Met662Thr). No NLRP3 mutation was detected in the patient’s parents. The mutation had previously been described in two cases of severe NLRP3-AID. Canakinumab was started at a dose of 150 mg every 2 months, but neurologic deterioration prompted a switch to anakinra 100 mg daily after a few months. The latter allowed clinical stabilization as well as normalization of cerebrospinal fluid analysis and inflammatory markers. The proband displayed unique manifestations of NLRP3-AID, namely the absence of an urticaria-like rash and peripheral eosinophilia with organ infiltration, as well as prominent serositis.

**Conclusion:** In summary, we hereby report a case of severe NLRP3-AID with peripheral and tissue eosinophilia and prominent serositis, but lacking urticaria. As a result, the absence of an urticarial rash should not curtail a diagnosis of NLRP3-AID in patients with other compatible manifestations. Furthermore, the association of autoinflammatory features and eosinophilia should raise suspicion for this diagnosis.

**Disclosure of Interest**

None Declared

### P27 Unravelling clinical and genetic spectrum of Mevalonate Kinase Deficiency (MVKD) in Libya

#### A. A. Abushhaiwia^1^, Y. Yusra AElfawires^1,2^

##### ^1^Paediatric Rheumatology, faculty of Medicine, Tripoli university ,Tripoli children's hospital; ^2^paediatric Rheumatology , faculty of Medicine, Tripoli university, Tripoli children's hospital , Tripoli , Libya

###### **Correspondence:** Y. Yusra AElfawires

**Introduction:** Hyper-IgD syndrome (HIDS, MIM #260920 and MIM #610377) is a rare spectral autosomal recessive autoinflammatory disorder caused by mutations in the mevalonate kinase (MVK) gene. HIDS share similar phenotype to the others hereditary periodic fever syndromes (HPFS), which include: recurrent fever, lymphadenopathy, hepatomegaly, rash, arthritis, abdominal pain. Specifically increased levels of biomarkers related to acute inflammation like serum amyloid A (SAA), C-Reactive Protein (CRP) is also a common finding to all HPFS. Serum levels of immunoglobulin D (IgD) is not a specific finding as it can also be present in other syndromes as PFAPA. A more specific feature, the aciduria mevalonica, can be found in the severe spectrum of patients with the deficiency of mevalonate kinase. The clinical presentation largely depends on the residual activity of mevalonate kinase ranging from virtually undetectable to a range of unfunctional but detectable levels. Due to decreased level of mevalonate kinase, RhoA remains unprenylated and increases the secretion of pro-inflammatory cytokines such as IL-1β that results in the development of the cardinal autoinflammatory symptoms. Rarity of the disease and lack of genetic testing for confirmation makes diagnosis challenging in Libya and in other middle-income countries. Interestingly colchicines do not prevent attacks, anti-TNF has a variable effect and only anti-IL1 is effective.

**Objectives:** To report for the first time the clinical and genetic findings in two patients from Libya.

**Methods:** We retrieved clinical data from patient’s records. Genomic DNA was isolated from peripheral blood and a target gene panel with all the 5 genes already known to be related to the HPFS was performed.

**Results: Case report**

An 11-year-old Arab Libyan girl, born of un-consanguineous parents. She has been evaluated, managed and admitted several times since the age of 50 days, without reaching a specific diagnosis. She presented to our Rheumatology clinic in January 2013, at age of 2 years and has been followed since. Presenting with recurrent attacks of fever (reaching 40.3°C), lasting 3-5 days, every 2–8 weeks accompanied by oral aphthae, abdominal pain and diarrhea. Associated with headache, abdominal pain, nausea, and vomiting frequently. Cervical LAP was almost always present in attacks. Leukocytosis (15,000mm3-26, 000mm3), increased ESR and CRP (50 100mg/L), the remaining variables were normal.IgD levels were normal (37mg/dL), negative urine movalenate kinase and elevated SAA (initially 2mg/L, later 199 mg\L and currently 5mg/L). Initially, attacks were occurring every 3 weeks, but frequency decreased as the child grew. Last year one attack occurred every 2-3 months. The diagnosis of FMF or HIgD syndrome was entertained initially and she was put on colchicines. However, she continued to have recurrent attacks despite high doses (1.5 mg) of daily colchicines.

Her 5-year-old sister (P2) had similar symptoms since the age of 2 years clinically characterized by recurrent attacks of fever that reached as high as 40.3°C associated with oral ulceration, joint pain and enlarged cervical lymph nodes. Headache, abdominal pain, nausea, and vomiting, similar to P1, were also present in some of the attacks. The initial attacks were occurring as frequently as every 3 weeks. Laboratory investigation during attacks revealed mild anemia. (Haemoglobin as low as 10.3 g/l) during the attacks with platelets were within normal limits, white blood cells count were as high as 24,400 always with fluctuant levels of acute reactant markers as erythrocyte sedimentation rate (ranged from 40 to 80 mm/h). She was put on colchicines but it did not achieve clinical control. Familial pedigree reconstruction allowed the suspicion of a recessive disorder and due to a suspicion of an inborn error of immunity a target gene panel was performed (MEFV, NRLP3, MVK, TNERS1A). Curiously a heterozygous mutation in exon 11 of the mevalonate kinase (MVK) gene variant c.1129GA and a premature stop codon c.2T>A, p.met1?. Therefore the clinical and diagnosis of MVK was established for the 2 children. We surrogated that the other mutation was missed in P1 and genetic sequencing in P1 is ongoing to confirm the other variant found in P2.

**Conclusion:** Here we report for the first time HIDS in Libya and we highlight red flags for the suspicion of such a specific disorder in children with recurrent fever. We also demonstrate the importance of genetic analysis for the final diagnosis as well as the right genetic evaluation in such a complex clinical scenario. Besides, the prevalence of hereditary autoinflammatory diseases remains unknown in Libya.

**Acknowledgments**

I would like to extend my appreciation and thanks to Dr Leonardo Oliveira Mendonca to revising this manuscript

**Disclosure of Interest**

None Declared

### P28 Deficiency of Interleukin-36 Receptor Antagonist deficiency (DITRA) with C.80T>C (P. LEU27PRO) mutation: report of the first Libyan child

#### A. A. Abushhaiwia^1^, Y. AElfawires^2^, N. Alhouni^3^

##### ^1^Paediatrics Department, Faculty of Medicine, Univeristy of Tripoli,Tripoli Children Hospital; ^2^Paediatrics Department, Faculty of Medicine, University of Tripoli, Tripoli Children Hospital; ^3^Dermatology Department, Tripoli Central Hospital, Tripoli, Libya

###### **Correspondence:** A. A. Abushhaiwia

**Introduction:** Loss-of-function mutations along the IL36RN gene defines a recently described recessively inherited autoinflammatory syndrome named deficiency of IL-36 receptor antagonist (DITRA). This genetically determined deficiency was first described in a subgroup of patients with generalized pustular psoriasis. It is a life-threatening condition characterized by recurrent episodes of severe skin inflammation, with pustule development, associated with fever, malaise, extracutaneous involvement, neutrophilia and a marked acute phase response. Recently there was a possible genotype and phenotype correlation regarding disease severity.  It was surrogated whether the presence of mutations that accounted for critical protein expression could be associated to severe DIRA phenotypes observed. At least, 10 null mutations with complete absence of the IL36RN antagonist (eg, c.80T>C/p. Leu27Pro) were associated with severe clinical phenotypes compared with mutations with decreased or unchanged protein expression.

**Objectives:** we report for the first time DITRA in a Libyan  child. We also briefly discuss potential provisional therapeutical interventions for the treatment of this rare disorder in middle income countries where neither anti-IL1 nor  anti-IL12/23 are available.

**Methods:** We retrieved clinical data from patient’s records. Genomic DNA was obtained from peripheral blood. A direct sequencing of the coding exons and the flanking splice signals of the IL36RN gene was performed using standard procedures.

**Results: Case Report**

A 4 year-old Arab Libyan boy, born from non-consanguineous parents came to our attention due to a suspicion of a systemic autoinflammatory condition. Of note, the index patient had two healthy brothers and an uncle diagnosed with psoriatic arthritis.  At the age of  2 years recurrent and severe episodes of generalized pustular psoriases requiring many hospital admissions started. Initially,  he was managed as of pustular psoriasis after a 6-month history of appearance. Skin lesions were characterized by diffuse pustules involving cheeks, perioral area associated with mouth ulcers. A rapidly progression of his condition could be observed characterized by worsening of general condition and generalization of the rash over different body parts.  Other clinical pictures frequently observed was fever; psoriatic nail changes (e.g. Pitting and onychomadesis) and ichthyosis altogether with joint involvement limiting his activity. Rash became severe with discharging pustules and failure to thrive could be observed. At his admission, widespread presence of scaly erythematous plaques with pinhead pustules involving the scalp, face, extremities, torso, and buttocks. The palms and soles were also affected. Nail pitting and onychomadesis. Marked hypertrichosis was also noted. At his admission a complete blood count demonstrated anemia HGB 8.1 g\dl and leukocytosis.However, during febrile episodes the patient’s white blood cell count increased to 17,000/ll with neutrophilia of 92%, ESR 50mm\h, and elevated CRP 20mg\dl level. No other condition, such as infection, could be observed over the time.  A skin biopsy demonstrated parakeratosis, spongiosis, and psoriasiform acanthosis, We could also observe an elevated level of  vitamin B12 757.90 pg\ml(reference range). Due to a suspicion of a systemic autoinflammatory condition, a genetic sequencing was requested. A homozygous already described mutation in the  IL36RN was found  (c.80T>C; .pLeu27Pro), thus confirming the diagnosis of DITRA.Once neither anti-IL1 (anakinra) neither anti-Il12/23 (secukinumab) is available in Libya , high doses of systemic steroids was initiated and could achieve clinical control. In order to avoid prolonged use of  steroids, the patient’s dose was gradually tappered. However, cutaneous exacerbation could be observed and an steroid spared agent, methotrexate, at a dose of 7.5 mg once per week was therefore added. Under this treatment, the patient progressed towards a good general condition. However, there wasn’t  complete improvement of his skin rash neither control of the febrile episodes.

**Conclusion:** DITRA is a rare disease that has to be considered in GPP with systemic inflammation and fever. Increased awareness of this distinct type of GPP caused by mutations in IL36RN is of major importance for future potential therapy directed at the IL36RA protein.

**Acknowledgments:**

**Disclosure of Interest**

None Declared

### P29 An early onset of skin rash: a case of 4-month-old infant

#### A. Mauro^1^, O. Francesca^2^, G. Antonietta^1^, M. Rosa^1^, A. Sabrina^1^, A. Francesca^1^, D. Carolina^1^, M. Stefania^1^, S. Esposito^1^, V. Tipo^1^

##### ^1^Emergency Department; ^2^Pediatrics, Aorn Santobono-Pausilipon, Naples, Italy

###### **Correspondence:** A. Mauro

**Introduction:** Dermatological diseases are a challenge for pediatricians. The skin is often involved in monogenic autoinflammatory syndrome with a wide range of cutaneous lesions.

**Objectives:** We describe a weird case of an early onset hyperkeratosis and maculo-papular rash.

**Methods:** A 4-month-old male was admitted to our Paediatric Emergency Unit due to history of rash since the age of one month. He was born to non consanguineous parents, at 39 weeks of gestational age following an uncomplicated pregnancy and delivery with normal neonatal phenomena. He had a good growth with no feeding difficulties and without any comorbidity.

At the beginning, suspecting atopic dermatitis, he was treated with topical corticosteroid, emollients, and oral antihistamine. Because of the lack of improvement of the eruption and no relief of the itch, the family paediatrician assumed a cow's milk proteins allergy, so the baby started feeding with a hydrolysed milk. Even this treatment had no success.

On examination, the child was apyretic, alert but too irritable. He presented whole body extended rash characterized by red papules, excoriation, and hyperkeratotic whitish crusts with crackings on the palmo-plantar surfaces.

Due to the early age of the onset of rash and the lack of rash improvement with previous treatment, the hypothesis of autoinflammatory disease was made, such as DITRA Syndrome.

**Results:** Routine blood tests revealed a leukocyte count of 44,60 × 10^9^ cells/L with a very high eosinophil count of 20,39× 10^3^ cells/L. Inflammatory markers (C-reactive protein , Erythrocyte sedimentation rate , Serum Amyloid A ) were within normal range . The infant screening for immunodeficiency, differential count, T-cell and B-cell subsets, quantitative immuno-globulins, and HIV test were all normal.

In consideration of absence of fever ad normal level of inflammatory markers, we decided to postpone genetic tests and we performed an accurate anamnesis and we evaluated the patient clinically again

During the baby examination our physicians noticed on the baby mother’s arms an erythematous papular and excoriated eruption with scratching. For this reason we performed an accurate anamnesis and a dermatoscopic examination of skin scrapings revealing numerous scabies mites and eggs.

**Conclusion:** A diagnosis of crusted or Norwegian scabies was made and he was treated with oral antihistamine, overnight application of topical 5% permethrin. The patient was discharged from the hospital after three days of treatment with complete resolution of cutaneous lesions and normalization of the blood tests. The aim of this case report is underlying the importance of an accurate anamnesis and clinical examination, also of the parents, to avoid delayed diagnosis

**Disclosure of Interest**

None Declared

### P30 Tumor Necrosis Factor Receptor-associated Periodic Syndrome (TRAPS) masquerading as chronic urticaria in a teenage girl

#### R. Naddei, R. Alfani, F. Mozzillo, T. Lastella, M. Amico, M. C. Carrella, M. Tripodi, M. Alessio

##### Pediatric Rheumatology Unit, Mother and Child Department, University of Naples Federico II, Naples, Italy

###### **Correspondence:** R. Naddei

**Introduction:** Skin manifestations are frequent in systemic auto-inflammatory diseases (SAIDs). Skin rash frequently accompanies systemic symptoms such as fever, malaise and joint involvement during recurrent autoinflammatory episodes.

**Objectives:** To describe clinical presentation of a young girl diagnosed with Tumor Necrosis Factor receptor-associated periodic syndrome (TRAPS) at the age of 16 due to chronic urticaria.

**Methods:** Case report.

**Results:** A 13-year-old girl referred to our Unit for an urticarial rash, started one month earlier. Single skin lesions lasted up to 24 hours, were localized at trunk and limbs and followed by arthralgias and then, 2 weeks later, by low-grade evening fever (up to 37.5 °C). The rash was mildly pruritic. Since the second day after rash onset, she had been treated with oral antihistamines and betamethasone (0,03 mg/kg/day) for 2 weeks, followed by oral methylprednisolone for 4 days (0,2 mg/kg/day) with only partial improvement of skin lesions. The day after glucocorticoid withdrawal, she presented fever (up to 39.5°C) and rash worsening, thus she was admitted to our Unit. Her past medical history was unremarkable, except for adenotonsillectomy performed at the age of 7 due to sleep apneas. Her father was affected with psoriasis and, since adulthood, episodic hidradenitis. At presentation, she presented papular wheals at trunk and face and coxalgia at passive flexion. Laboratory investigations showed marked elevation of acute phase reactants (C-reactive protein, CRP, 120,9 mg/L with normal values <5 mg/L and erythrocyte sedimentation rate, ESR, 79 mm/h) and slight neutrophilic leucocytosis (neutrophils 9290 cells/mmc). Fibrinogen was elevated (681 mg/dl) while procalcitonin was negative. No evidence of infection was detected at a thorough serological and microbiological screening. Antinuclear antibody, rheumatoid factor and complement were normal. Abdomen ultrasound showed slight hepatosplenomegaly. Autoimmune thyroiditis with normal thyroid function was detected. Bone marrow aspiration revealed no signs of hemophagocytosis or cellular atypia. Skin biopsy showed dermal neutrophils and lymphocytes infiltrate. During the hospitalization, lasted two weeks, the patient presented spontaneous progressive clinical amelioration with fever and arthralgias disappearance few days after admission and progressive reduction of inflammatory indexes. At discharge, CRP was 6,6 mg/L, ESR was 30 mm/h and a mild elevation of Serum amyloid A (SAA) was detected (60,1 mg/L, normal values <3,47 mg/L). One month later, CRP, ESR and SAA were normalized. Spontaneous sporadic episodes of short-duration (less than 24 hours) urticarial rash persisted over the following three months and then disappeared. She presented overall well-being for the following two years. At the age of 15, she newly developed persistent spontaneous urticarial rash without fever or arthralgias. Skin lesions appeared mainly during evening, were pruritic, localized at face, thighs and back of the hands and steroid-dependent. Inflammatory markers were newly elevated: CRP was 20 mg/L, ESR 40 mm/h and SAA 166 mg/L. Due to elevation of inflammatory markers in association with rash reappearance, next generation sequencing (NGS) analysis for SAIDs was performed and showed the heterozygotic variant c.362G>A, p.(Arg121Gln) of *TNFRSF1A* gene, consistent with TRAPS. Therefore, about six months after chronic urticaria reappearance, the patient started subcutaneous anakinra (100 mg/day) with prompt resolution of skin rash. At one-month follow-up after anakinra starting, the patient did not experience any urticaria relapse and inflammatory markers were normal.

**Conclusion:** In this 16-year-old female, TRAPS was diagnosed due to spontaneous chronic urticaria, associated with inflammatory markers increase. She presented fever and arthralgias only at the onset, occurred at the age of 13. This case showed that chronic urticaria can characterize the clinical course of TRAPS, even though fever and other systemic signs may not be present. Elevation of inflammatory indices should give rise to the suspicion of SAID in patients with chronic urticaria, also in case of a late onset. Anakinra was effective in skin rash resolution in our patient. Written informed parental consent for publication was given.

**Disclosure of Interest**

None Declared

### P31 Chronic recurrent multifocal osteomyelitis: a first case report from Libya

#### A. A. Abushhaiwia^1^, Y. AElfawires^2^, A. Ateeq^3^

##### ^1^paediatric Rheumatology, faculty of Medicine,Tripoli university, Tripoli children's hospital; ^2^paediatric Rheumatology , Faculty of Medicine, Tripoli university , Tripoli children's hospital; ^3^paediatric Rheumatology , Tripoli children's hospital , Tripoli , Libya

###### **Correspondence:** A. A. Abushhaiwia

**Introduction:** Chronic recurrent multifocal osteomyelitis (CRMO) is a rare idiopathic inflammatory disease that affects mainly children and young adults. The clinical signs and symptoms are nonspecific, hindering and delaying diagnosis. Radiological and histopathological tests are essential for its definition .To our knowledge, there are no reports of CRMO in Libya, this is the first case report, which was initially misdiagnosed and treated by multiple courses of different types of antibiotics is presented. We describe the clinical and laboratory features and treatment of it

**Objectives:** To present a case of CRMO to demonstrate the diagnostic importance of clinical, laboratory, mainly, imaging tests, and avoids misdiagnoses or late diagnoses**.**

**Methods:** The diagnosis of CRMO was based on evidence of recurrent osteomyelitis with radiographic evidence of chronic osteomyelitis involving at least two sites in the absence of infectious cause in a child less than 14 years old

**Results:** A 14-year-old Libyan boy, born of non-consanguineous healthy parents, Was referred to our Department of Paediatric Rheumatology in December 2020 with a history of episodes of bone pain in different sites sometimes associated swelling and hotness but no redness, Symptoms had been started since at the age 10 years, he was evaluated several times without a specific diagnosis being made and he had received various antibiotics and several admissions as a case of acute or chronic osteomylities and bone biopsy had been taken from different site (left tibia, left and right ulna) revealed chronic osteomylities and  no bacterial growth in the  different hospitals in Libya and outside Libya without specific diagnosis, in  2016 the patient sought medical care because of  aching pain over the right anterior aspect of leg associated with swelling, hotness but no redness, the pain not radiated, was worse with rest, The patient was otherwise healthy with no history of fevers, chills, or weight loss. He had neither history of previous surgeries nor a family history of bone or joint abnormalities, including tumors. He underwent MRI left leg, which revealed acute osteomylities, the patient’s white blood count (WBC) was normal, erythrocyte sedimentation rate (ESR) was normal 5mm/hr (0–20 mm/hr), and C-reactive protein (CRP) was normal at <5mg/L (<5 mg/L). He received oral antibiotic, an open bone biopsy was performed on right tibia in Italy and revealed no bacterial growth, no specific pathological finding seen in sub matted materials treated with broad spectrum antibiotics without improvement but he had spontaneous remission for one year. In 2018 he had another episode of bone pain but over the extensor aspect of right forearm associated with swelling and hotness, MRI right forearm that showed proximal ulna metaphysis periosteal with evidence of intra medullar small fluid collection of high signal intensity on T2 image, low signal intensity on T1 image surrounded by bone marrow edema on STIR image concluded proximal acute osteomyelitis, in order to exclude malignancy CTscan right forearm with 3D post processing was normal, received also antibiotics , sought medical advice in Jordan  for more diagnostic workup due Radiologist suggested the lesion could be due to tumoral involvement like Ewing’s sarcoma, or oesteomylities or eosinophilic granuloma , MRI right  forearm with IV contrast  in Jordan was revealed diffuse and significant bone infiltration and bone marrow edema involving the proximal third of ulna associated with periosteal edema.CTscan right forearm  without contract showed suspicious infiltrative bone disease in proximal two third of ulna with periosteal reaction and elevation. According to the radiographic findings, the patient was candidate for open 2nd time bone biopsy from right ulna showed chronic osteomylities no bacterial growth, received oral antibiotics and NSAID (ibuprofen syrup) for 3 weeks. His symptoms disappeared for 2 years. In June 2020 he had pain in left forearm, 3^rd^ biopsy performed MRI of the left forearm was hypointense in T1 and hyperintense in T2 and STIR, suggesting an inflammatory process. He assumed NSAID for a week. 6 months later, the patient came to our Department because of pain and swelling in left forearm. Based on his clinical history, physical examination, laboratory, laboratoristic and radiological findings and bone biopsy CRMO was diagnosed.  Patient started anti-inflammatory treatment (naproxen) for 3 months and his conditions improved. NSAID treatment has been suspended.

**Conclusion:** CRMO should be considered in any a child with chronic bone pain to perform a correct diagnosis and start an adequate treatment. Timely diagnosis of these disorders is crucial to avoid potential complications

**Disclosure of Interest**

None Declared

### P32 Emotional distress as a trigger for PFAPA attacks

#### Y. Levinsky^1^, Y. A. Butbul^2^, T. Tal^1^, M. Natour^1^, K. Kedar^1^, N. Dagan^1^, G. Amarilyo^1^, L. Harel^3^

##### ^1^Schneider Children's medical center of Israel, Petah tikva; ^2^Rambam Health Care Campus, Haifa; ^3^ Schneider children's Medicalcenter of Israel, Petah Tikva, Israel

###### **Correspondence:** Y. Levinsky

**Introduction:** There is scarce information regarding potential triggers for PFAPA attacks.

**Objectives:** To examine whether an emotional stress serves as a trigger for these attacks.

**Methods:** Active PFAPA Patients aged 3-12 years from two Israeli centers were enrolled. Patients parents were reached via phone call and asked about the occurrence of PFAPA attacks in the preceding 2 weeks of a "stressful period" which was assumed to be (and later validated by an emotional distress scale questionnaire) within two weeks of returning to school after the first Israeli COVID-19 lockdown. Each patient served as its own control and parents were re-reached two weeks during summer break where COVID-19 outbreak was in remission (i.e. un-stressful period). The difference between occurrences of attacks during these 2 periods was recorded

**Results:** One hundred and six pediatric patient were enrolled in the study. Mean age was 7.37± 2.9. The mean emotional distress questionnaire was higher in the first period compared to the second period (35.6± 8.1 versus 32.1± 7.7, respectively, P = 0.047). In the "Stressful period" 41 (38.7%) reported at least 1 attack during the preceding 2 weeks compared to 24 (22.6%) in the "unstresfull period" (p = 0.017).

**Conclusion:** Here we provide first evidence that emotional stress may induce PFAPA attack.

**Disclosure of Interest**

None Declared

### P34 Efficacy of jak-inhibitor in treatment of a patient with ADA2 deficiency (DADA2)

#### V. I. Burlakov^1^, A. Kozlova^1^, E. Raykina^2^, M. Kurnikova^2^, N. Kan^1^, Z. Nesterenko^1^, S. J. Kelly^3^, M. S. Hershfield^3^, A. Shcherbina^1^

##### ^1^Immunology, ^2^Laboratory of Molecular Biology, Federal Research and Clinical Center of Pediatric Hematology, Oncology and Immunology, Moscow, Russia, Moscow, Russian Federation, ^3^Duke University School of Medicine, Durham, United States

###### **Correspondence:** A. Kozlova

**Introduction:** DADA2 is a rare disease caused by bi-allelic mutations in the *ADA2* gene. It is characterized by a variable phenotype that includes systemic inflammation, vasculitis and polyarteritis nodosa with strokes, humoral immunodeficiency and bone marrow failure. Recent studies demonstrated increased interferon signature in DADA2, placing this disease in the group of type I interferonopathies.

**Objectives:** To assess effectiveness of ruxolitinib in treatment of a patient with DADA2.

**Methods:** DADA2 diagnosis was confirmed by detection of homozygous c.39C>A, p.C13* *ADA2* mutation using targeted next generation sequencing panel and confirmed by Sanger sequencing. Plasma ADA2 enzymatic activity was measured in extracts of the dried plasma spots using the high-performance liquid chromatography method.

**Results:** A boy with DADA2 born to nonconsanguineous parents of the Crimean Tatar descent was referred to our Center at the age of 11 years. His symptoms included:

-Anemia, which presented in the first months of life, progressed over time with the need of monthly erythrocyte transfusions despite the treatment with steroids up to 2 mg/kg. Bone marrow smear showed isolated red cell hypoplasia.

-Livedo vasculitis since 5 years of age.

-Recurrent fevers.

-Periventricular ischemic lesions on the MRI brain scans at the age of 9 years.

Laboratory tests at admission showed also mild inflammatory activity, lymphopenia (Table 1) with decrease in all lymphocyte populations: CD3+CD4+ 0,196 cells/μl, CD3+CD8+ 0,139 cells/μl, CD19+ 0,009 cells/μl.

The patient was deficient in plasma ADA2 enzymatic activity 4,3 mU/g vs 75,9 mU/g for a healthy control sample (lab reference ranges for controls and carriers are 130 ± 53 U/g and 55 ± 22 U/g, respectively).

The patient was started on etanercept 0,8 mg/kg weekly with gradual discontinuation of steroids and vasculitis’ resolution, yet he developed multi-lineage cytopenia with positive Coombs test (Table 1), that was treated with steroids at 1 mg/kg for 3 months and a course of rituximab 4 x 375 mg/m^2^ with partial response. He developed severe Cushing syndrome.

He was then started on ruxolitinib 10 mg/m^2^ daily with gradual steroid discontinuation.

At the 12 months follow-up the patient was clinically asymptomatic, had no fever flares or signs of vasculitis. Laboratory tests showed marked increase of platelet and lymphocyte counts and normalization of all other parameters. He had no severe infections or signs of treatment toxicity.

**Conclusion:** DADA2 is a complex and diverse life-threatening disease, often with poor response to various modes of treatment. In our patient, ruxolitinib was well tolerated and effective in acquiring and maintaining remission of vasculitis and cytopenia. This experience deserves further investigation in a larger cohort of patients.

**Table 1 (abstract P34).** Treatment modalities and outcomes in patient with DADA2

Treatment	Clinical symptoms			Laboratory tests					
	Vasculitis	Hepato-splenomegaly	Fever	Hb, g/l	PLT, 10^9^/l	WBC, cells/μl	NEU, cells/μl	LYM, cells/μl	CRP, mg/l
Before treatment	+++	+++	+++	59	n/a	n/a	n/a	n/a	n/a
Steroids, 0,1-30 mg/kg	+	-	+/-	133	109	3740	2403	760	16,4
Etanercept, 0,8 mg/kg	-	-	-	52	49	610	200	270	4,62
Etanercept 0,8 mg/kg + steroids 1 mg/kg	-	-	-	144	62	3898	2447	535	1,6
Ruxolitinib, 10 mg/m^2^	-	-	-	154	91	5890	4317	967	1,4

Hb – hemoglobin, PLT – platelet count, WBC – white blood cell count, NEU – neutrophil count, LYM – lymphocyte count, CRP – C-reactive protein. Laboratory test results show median values for the observation time

**Disclosure of Interest**

None Declared

### P35 Safety and effectiveness of biosimilar of adalimumab ABP501 in the treatment of juvenile idiopathic arthritis: pera research group experience

#### F. G. Demirkan^1^, K. Ulu^2^, K. Öztürk^3^, S. G. Karadağ^4^, S. Özdel^5^, H. E. Sönmez^6^, F. Çakmak^1^, F. Demir^2^, B. Sözeri^2^, N. Aktay Ayaz^1^

##### ^1^Pediatric Rheumatology, Istanbul University/Faculty of Medicine; ^2^Pediatric Rheumatology, University of Health Sciences, Ümraniye Research and Training Hospital; ^3^Pediatric Rheumatology, Istanbul Medeniyet University, School of Medicine, Goztepe Research and Training Hospital; ^4^Pediatric Rheumatology, University of Health Sciences, Bakırköy Dr. SadiKonuk Training and Research Hospital, istanbul; ^5^Pediatric Rheumatology, University of Health Sciences, Dr. Sami Ulus Obstetrics and Gynecology, Pediatric Health and Disease Training and Research Hospital, Ankara; ^6^Pediatric Rheumatology, Kocaeli University, Kocaeli School of Medicine, Kocaeli, kocaeli, Turkey

###### **Correspondence:** F. G. Demirkan

**Introduction:** Over the past twenty years, biologic products and subsequently biosimilars, have dramatically enhanced the treatment of several chronic pediatric inflammatory conditions particularly juvenile idiopathic arthritis (JIA).

**Objectives:** We aimed to review the real-life data regarding adalimumab biosimilar treatment in children with JIA and make a contribution to the literature about its efficacy and safety.

**Methods:** This retrospective, multi-centric study included children with a rheumatic disease who were followed-up by tertiary pediatric rheumatology centers across Turkey. Patients were followed for at least 24-weeks from the initiation of adalimumab biosimilar treatment with prescription indications, clinical responses, disease activity scores, and adverse events reported.

**Results:** The study included 43 pediatric patients with a median age of 15.4±2.6 (5-18) years; 60 % of them were male. The diagnoses of the group were as follows: JIA in 41 patients (95%); uveitis secondary to sarcoidosis in 1 patient (2%) and iridocyclitis in 1 patient (2%). JIA categories were oligoarticular (n=12, 27%), enthesitis-related (n=20, 46%), psoriatic (n=6, 14%), and polyarticular (n=3, 7%). Seven patients with JIA developed uveitis during the disease course.

The first treatment option of the experts was adalimumab biosimilar (ADL) in 33, etanercept in 9, and secukinumab in 1 patient. A standard dose of ADL biosimilar 40 mg once-in-2-week was started to the patients. Etanercept was switched to adalimumab biosimilar due to allergic reactions (n=2), development/activation of uveitis (n=3), psoriasis activation (n=1), and arthritis (n=2). In one patient with psoriatic arthritis, as the arthritis was not responsive to secukinumab, ADL biosimilar switching was made.

In the 3^rd^ month of follow-up, uveitis was in remission in 5 patients. Clinical remission and response status were evaluated with Juvenile Arthritis Disease Activity Score 27 score. A significant decrease in the scores determined the clinical effectiveness of ADL biosimilar in JIA patients (p=0.001). One patient at the third month of therapy had compliance problems with the biosimilar, so a switch to the original ADL molecule was rendered.

Of the 41 patients who received ADL biosimilar; 34 patients had no active complaints during follow-up. No adverse events were observed.

**Conclusion:** In rheumatology, the majority of previous reports of biosimilars were concerning adults, yet data with reference to the effectiveness and safety of biosimilars in pediatric rheumatic diseases is scarce.^2^ Real-life data on drug safety and efficacy need to be collected to increase evidence base data and in this context, this report reflects the prescription patterns and treatment strategies for a frequently preferred biosimilar drug in pediatric rheumatology with real-life experience for the first time.^2,3^ ABP-501 is a suitable and cost-effective treatment option for patients with the chronic rheumatic disease when adalimumab use is indicated.

**Keywords:** adalimumab, biosimilar, amjevita, juvenile idiopathic arthritis

**Disclosure of Interest**

None Declared

### P36 Anakinra and canakinumab for patients with R92Q-associated autoinflammatory syndrome: a multicentre observational study from the AIDA Network

#### C. Gaggiano^1^, D. Rigante^2^, J. Hernández-Rodríguez^3^, A. Vitale^1^, M. Tarsia^1^, A. Soriano^4^, G. Lopalco^5^, M. A. Jaber^6^, R. Giacomelli^7^, E. Więsik-Szewczyk^8^, M. Cattalini^9^, M. Frassi^9^, M. Piga^10^, G. Ragab^11^, F. Zunica^9^, A. Floris^10^, V. Sabato^12^, M. T. Hegazy^11^, O. Araújo^3^, L. Pelegrín^3^, B. Frediani^1^, A. Fabbiani^1^, A. Renieri^1^, S. Grosso^1^, L. Cantarini^1^

##### ^1^University of Siena, Siena; ^2^Fondazione Policlinico Universitario A. Gemelli IRCCS and Università Cattolica Sacro Cuore, Rome, Italy; ^3^Hospital Clinic of Barcelona, IDIBAPS, University of Barcelona, Barcelona, Spain; ^4^Arcispedale Santa Maria Nuova-IRCCS, Reggio Emilia; ^5^University of Bari, Bari; ^6^Santa Chiara Hospital, Trento; ^7^University of L'Aquila, L'Aquila, Italy; ^8^Central Clinical Hospital of the Ministry of National Defense, Military Institute of Medicine, Warsaw, Poland; ^9^University of Brescia and Spedali Civili of Brescia, Brescia; ^10^University and AOU of Cagliari, Cagliari, Italy; ^11^Cairo University, Cairo, Egypt; ^12^University of Antwerp and Antwerp University Hospital, Antwerpen, Belgium

###### **Correspondence:** C. Gaggiano

**Introduction:** The gold standard treatment of TNF receptor-associated periodic syndrome (TRAPS) is the inhibition of IL-1, the final effector of the stress-response mechanisms activated by the deregulated cellular homeostasis in TRAPS patients. Given the uncertainty about the molecular mechanism and pathogenic significance of the R92Q variant, the therapeutic response to IL-1 inhibitors in patients with recurrent inflammatory attacks due to the presence of this specific mutation is worth an extensive investigation.

**Objectives:** This study aims at describing the therapeutic outcome of patients carrying the R92Q variant in the *TNFRSF1A* gene treated with anakinra (ANA) or canakinumab (CAN) and identifying any factors predictive of complete response to IL-1 inhibition.

**Methods:** This is an observational study involving 13 international centres from the AIDA Network. Demographic, clinical, laboratory, clinimetric and therapeutic data of patients treated with ANA or CAN for recurrent inflammatory attacks due to the presence of the R92Q variant were retrospectively collected and analysed.

**Results:** Data about 20 treatment courses with IL-1 inhibitors (16 with ANA and 4 with CAN) from 19 patients were collected. Mean age at disease onset was 20.2±14.8 years. In 5 cases (26%) the R92Q variant was found in a family member affected by recurrent fever. The therapeutic response was complete in 13(68%) and partial in 2 patients (11%); treatment failure was observed in 4 cases (21%). Median AIDAI decreased from 10(IQR 28) to 0(IQR 1) at the 12-month follow-up visit (*p*<0.001). Mean ESR and median PCR dropped respectively from 40.8±24.8 to 9.1±4.5 mm/h (*p*<0.001) and from 3.0 (IQR 1.9) to 0.3 (IQR 0.3) mg/dl (*p*<0.001) after 12 months of treatment. A steroid-sparing effect was observed from the third month of treatment (*p*<0.01). Thirteen patients (65%) were still on treatment at the last follow-up visit (median duration of treatment 17 (IQR 38) months). The presence of R92Q mutation in a symptomatic relative (*p*=0.022), the relapsing remitting disease course (*p*<0.001) and the presence of migratory erythematous skin rashes during fever attacks (*p*=0.005) were associated with complete efficacy of IL-1 inhibitors.

**Conclusion:** R92Q patients showed a favourable response to ANA and CAN, particularly when the mutation segregated in a family member and when a relapsing-remitting disease course or TRAPS typical skin rash were observed. In the subgroup of patients not taking advantage of IL-1 blockage different molecular mechanisms underlying the autoinflammatory picture are likely to exist.

**Disclosure of Interest**

None Declared

### P37 Fetal exposure to canakinumab: a report of three pregnancies

#### U. Ilgen^1^, S. Eyüpoğlu^2^, E. Yayla^3^, O. Küçükşahin^4^

##### ^1^Rheumatology, Trakya University, Edirne; ^2^Nephrology; ^3^Rheumatology, Ankara University; ^4^Rheumatology, Ankara Yıldırım Beyazıt Univeristy, Ankara, Turkey

###### **Correspondence:** U. Ilgen

**Introduction:** Interleukin-1 (IL-1) blockade during pregnancy is well tolerated but safety data is limited particularly for canakinumab, under which a total of only ten pregnancies were reported to date.

**Objectives:** To provide additional safety data of canaknumab based on three pregnancies of two cases, one with familial Mediterranean fever (FMF) and the other with Muckle-Wells syndrome (MWS).

**Methods:** A data sheet consisting of general disease characteristics and a detailed pregnancy and postpartum follow-up information consisting of the age and disease activity at conception, timing of the last canakinumab dose with respect to pregnancy week, disease activity and canakinumab use during and after pregnancy, type and timing of delivery and feeding, status of the baby during intrauterine, neonatal, and infancy period, was completed for each pregnancy.

**Results:** The patients were 29- and 31-year-old women with MWS and FMF, respectively. The first had two pregnancies under canakinumab. Both were complicated by renal amyloidosis but were attack-free with normal acute phase reactants and stable proteinuria (240 mg/day and 550 mg/day, respectively) for years before conception and both were under monotherapy with canakinumab. Pregnancy outcomes were provided in the Table. The first patient with a mid-trimester miscarriage held canakinumab 14 weeks before the following conception until the 31st week of gestation. During this period she had attacks partially responsive to low-to-moderate dose steroids and on-demand anakinra but proteinuria progressed to 2600 mg/day with persistent elevation in acute phase reactants, for which canakinumab was resumed at the 31st week of gestation. Both patients were under canakinumab over the entire course of breastfeeding. No significant infections were reported in the infants. Anti-hepatitis B surface antigen titers were 218 and 470 IU/L in the sixth month and 402 and 620 IU/L in the second year of age (Three doses of hepatitis B vaccine in the 0, 1, and 6 months were administered according to the national immunization program).

**Conclusion:** Considering all 13 reported cases of pregnancy under canakinumab, two resulted in miscarriage, one in the first and one in the second trimester. Underlying disease was active in the one with the first trimester miscarriage. All fetuses were exposed to canakinumab during embryogenesis with no reported anomalies. Most cases continued canakinumab throughout pregnancy with no preterm labor or severe infection in the infant. Hepatitis B vaccination in the infant was shown to be successful in two cases.

**Table 1 (abstract P37).** Pregnancy outcomes under canakinumab

	Age at pregnancy	Last cana dose before conception	Doses and duration of cana after conception	Date and type of delivery	Birth weight	APGAR score	Fetoplacental anomaly, IUGR, or developmental delay	Feeding	Current age of the child
1*	25 years	150 mg, 2 weeks	150 mg at 6 and 14 weeks	16 week, miscarriage	-	-	No phenotypic or chromosomal anomaly	-	-
2*	26 years	150 mg, 14 weeks	150 mg at 31 weeks and 4-to-6-weekly thereafter	37 week, C/S	2.9 kg	8,9,10	No anomaly, IUGR, or delay	Breastfed 16 months	2.5 years
3	28 years	150 mg, 4 weeks	150 mg 8-weekly throughout pregnancy	39 week, C/S	3.4 kg	9,10,10	No anomaly, IUGR, or delay	Breastfed 8 months	2 years

cana, canakinumab; C/S, Caesarean section; IUGR, intrauterine growth retardation. * the same patient

**Disclosure of Interest**

None Declared

### P38 The comparison of vaccination rates of children with rheumatic diseases and their healthy peers

#### Ö. Akgün^1^, F. G. Demirkan^1^, F. Çakmak^1^, M. Erdemir^2^, G. Keskindemirci^2^, G. Kavrul Kayaalp^1^, N. Akay^3^, R. Eker Ömeroğlu^1^, E. G. Gökçay^2^, N. Aktay Ayaz^1^

##### ^1^Pediatric Rheumatology; ^2^Social Pediatrics; ^3^Pediatrics, Istanbul Faculty of Medicine, Istanbul, Turkey

###### **Correspondence:** Ö. Akgün

**Introduction:** Patients who are followed-up in the pediatric rheumatology outpatient clinic are at risk due to immune dysfunction caused by both their primary disease and by the drugs they use. As they are more prone to infection, these patients should be protected carefully from vaccine-preventable diseases.

**Objectives:** Our aim is to find out the vaccination rates in patients diagnosed with a pediatric rheumatologic disease (PedRD) and to assess their vaccination status comparing with their healthy peers. Another objective of the study is to evaluate factors that may lead to incomplete vaccination in patients with PedRD.

**Methods:** The electronic healthcare recording system data of patients with PedRD who were followed in the pediatric rheumatology out-patient clinic in Istanbul University Faculty of Medicine between October 2019 and October 2020 were reviewed and 102 patients (aged 0-18) whose vaccination records were available are included in the study. One hundred two age-matched peers without any chronic disease and whose vaccination data were available through electronic healthcare recording system were included as the control group. Incomplete vaccination status and missing vaccines were identified according to the national vaccination schedule individualized by age. Fifty- one patients (50%) have the diagnosis of juvenile idiopathic arthritis (JIA), 38 patients (37.2%) have the diagnosis of periodic fever syndromes and 13 patients (12.74%) have the diagnosis of connective tissue diseases.

**Results:** The proportion of the females was 67% in the patient group (n=68) and 57% in the control group (n=58). The median age of the patient group was 8.1(1.9-15.5) and the mean age control group was 7.5(±3.4) years. There was no difference between the patient and the control group in terms of age and gender. The median age at diagnosis was 3.3(0.3-11.4) years. Incomplete vaccination rate was 35.3 % in the patient group and 6.9 % in the control group (P<0.0001). The most common neglected vaccine was the first dose of oral poliovirus vaccine (OPV) (n=19, 18.6%), followed by the second dose of OPV (n=18, 15.7%) and the first dose of measles, rubella, mumps vaccine (n=13, 12.7%). Patients were divided into four groups according to the immunosuppressive antirheumatic treatments they received: nonbiologic disease modifying antirheumatic drugs (DMARDs), biological DMARDs, nonbiological + biological DMARDs and others (colchicine, non-steroidal anti-inflammatory drugs, hydroxychloroquine). Although the patients who received biologic therapy had the highest rate of missing vaccine at 43%, no significant difference was detected between the treatment groups.

**Conclusion: :** It is important to determine the vaccination status of patients followed by PedRD and to take measures against vaccine-preventable diseases by increasing the awareness of physicians and patients in this regard. As a result, questioning the status of the vaccine and to plan a catch-up immunization schedule for patients with incomplete vaccines should be a part of the routine rheumatologic examinations. Electronic registration systems can fill an important gap in solving this problem by giving warnings in case of vaccine deficiency which can be convenient in busy outpatient clinics.

Key Words: Vaccination, Pediatric rheumatic disease, biologic therapy

**Disclosure of Interest**

None Declared

### P39 Willingness to get the COVID-19 vaccine among children with rheumatic diseases: a survey study to parents

#### Ö. Akgün, F. G. Demirkan, F. Ataman Çakmak, G. Kavrul Kayaalp, O. Köker Turan, A. Tanatar, N. Aktay Ayaz

##### Pediatric Rheumatologist, Istanbul Faculty of Medicine, Istanbul, Turkey

###### **Correspondence:** Ö. Akgün

**Introduction:** Severe acute respiratory syndrome coronavirus-2 (SARS-CoV-2) is the virus that causes coronavirus disease 2019 (COVID-19), first reported from the Wuhan city of China in December 2019, affected all the world in a few months and became a global health emergency of primary international concern and was announced a pandemic by the World Health Organisation. Although the disease is showing a milder course in children, vaccination of this age group may be important due to increased number of the multisystem inflammatory syndrome (MIS-C) and vaccines role in prevention of the spread of infection in children who have had asymptomatic disease. As vaccine refusal and hesitancy are increasing around the world, a new problem has emerged for children with pediatric rheumatic diseases who are already prone to infection.

**Objectives:** This study aimed to investigate the parental acceptance of COVID-19 vaccination for children with chronic rheumatic diseases when the vaccine is approved in childhood and to evaluate the background characteristics of willingness to get COVID-19 vaccine*.*

**Methods:** The study included an analysis of a cross-sectional online questionnaire to the parents of the patients diagnosed with a chronic rheumatic disease at the Pediatric Rheumatology Outpatient Clinic of Istanbul Faculty of Medicine. A message containing a link to the actual questionnaire was sent to their phones simultaneously which covers their view for the SARS CoV-2 infection, sociodemographic data, thoughts about the vaccine, their child's behavior during the pandemic related to the disease, and completed by self- response method. Clinical information was accessed from the medical records of the patients.

**Results:** The prevalence of parents’ acceptance of COVID-19 vaccination for their children was 39.5% (30/76). The proportion of parents who were hesitant about the vaccination of their children was 47.4%. Whereas, the rates of acceptance of the vaccine by mothers and fathers themselves were 48.7% and 52.6%, respectively. Factors for vaccine referral were side effects (47.4%), limited information about the vaccine (45.2%) and possible ineteraction of the vaccines with the medications of the patients (40.2%). Parents were able to give multiple answers to this question. There was no significant relationship between the parents' educational status and their acceptance of the vaccine (respectively p=0.09, p=0.10)

**Conclusion:** Children can serve as a reservoir that will undermine efforts to end the pandemic. While the vaccines have yet no approval for children <18 years, vaccinating children against the COVID-19 virus will contribute to the pandemic control, and the recovery of the global economy. The preliminary results of this survey targeting parents may be guide for physicians while promoting vaccination in the near future.

key words: Covid-19, vaccine, survey. pediatric rheumatic disease

**Disclosure of Interest**

None Declared

### P40 Live attenuated MMR booster vaccine in children with rheumatic disease: a single center study

#### F. Çakmak^1^, Ö. Akgün^1^, F. G. Demirkan^1^, A. Tanatar^1^, G. Kavrul Kayaalp^1^, G. Keskindemirci^2^, R. Eker Ömeroğlu^1^, E. G. Gökçay^2^, N. Aktay Ayaz^1^

##### ^1^Pediatric Rheumatology; ^2^Social Pediatrics, Istanbul Faculty of Medicine, Istanbul, Turkey

###### **Correspondence:** F. Çakmak

**Introduction:** Juvenile Idiopathic Arthritis (JIA) is a heterogeneous, chronic inflammatory disease of unknown cause which begins before 16 years of age. Vaccination is the most effective way to prevent infectious diseases. In May 2017, European League Against Rheumatism-Paediatric Rheumatology European Society (EULAR-PReS) Task Force for Vaccination, based on small case series and expert opinion updated the recommendations for live vaccines in children.

**Objectives:** In this study, it is aimed to collect the data of patients with JIA and other rheumatic diseases who received live attenuated booster measles-mumps-rubella (MMR) vaccine during treatment with biological therapy and present if any disease flares or adverse reactions occured after vaccinations.

**Methods:** The files of the patients followed-up in İstanbul University Istanbul Faculty of Medicine with the diagnosis of JIA or other rheumatic diseases who received biological treatment were analyzed retrospectively. According to the current national vaccination schedule, the MMR vaccine booster dose is administered in the first year of primary school, so we evaluated from electronic vaccination records whether any of our patients had received live attenuated booster vaccinations for MMR while being treated with biologics. Demographic data and information on disease subtypes, disease activity at the time of the booster vaccination, and adverse reactions after vaccinations (disease flare or vaccine-related event) were collected from the files of the patients retrospectively.

**Results:** Among 72 patients under biologic therapy, 14 patients (7 girls and 7 boys) had received live attenuated booster measles-mumps-rubella (MMR) during treatment with biologics. The mean age of diagnosis was 43,7 (9-73) months and the mean age of booster vaccination was 76,2 (70-84) months. The mean follow-up time was 54 months (23-84) and the mean time to initiate biologics before vaccination was 14 months (4-45). Seven of these patients had oligoarticular juvenile idiopathic arthritis (JIA), 3 had systemic onset JIA, 2 had enthesitis related arthritis, 1 had psoriatic arthritis and 1 had colchicine resistant familial Mediterranean fever. Eight patients received booster MMR while on biologic treatment only and 6 were under both methotrexate and biologic therapy. During vaccination, 4 (%30) patients had low and 3 had moderate (%23) disease activity, and 7 (%50) patients were in remission on medication.

Out of 14 patients who had MMR booster, only 1 patient with the diagnosis of systemic onset JIA under methotrexate and tocilizumab therapy had a maculopapular rash after vaccination. None of the patients reproted any disease flare.

**Conclusion:** In the literature, data regarding live attenuated vaccination of patients under biological therapy is limited. At the evaluation of our cohort from electronic vaccination records and patient files, it was noticed that the live attenuated booster MMR vaccine was seemingly safe for children with rheumatic diseases who were receiving biologic therapy. However, to conclude that live vaccination is effective and safe, this data should be supported by multicentric and larger cohorts.

**Disclosure of Interest**

None Declared

### P41 Towards the Identification of disease-modifying factors in CTLA4 insufficiency

#### B. Grimbacher, N. Mitsuiki, M. Krausz, L. Gámez-Díaz, C. Schwab, F. Emmerich, N. Camacho Ordonez, S. Posadas-Cantera, H. Hengel, M. Proietti, A. Caballero-Garcia-de-Oteyza

##### Institute for Immunodeficiency, Universital Hospital Freiburg, Freiburg, Germany

###### **Correspondence:** B. Grimbacher

**Introduction:** Heterozygous mutations in *CTLA4* lead to an inborn error of immunity characterized by immunodeficiency and multi-organ autoimmunity. Interestingly, the penetrance of these deleterious mutations is only about 70%, and their expressivity is highly variable.

**Objectives:** To identify modifying factors explaining the reduced penetrance and the variable expressivity.

**Methods:** We compared two groups of patients with CTLA4 mutations: 1) affected mutation carriers; 2) unaffected mutation carriers. We analyzed the exome sequence, the HLA type, the microbial stool content, the infection history, and epigenetic signatures. In addition, we looked for somatic mutations in *CTLA4* and pathway-related genes.

**Results:** When analyzing the WES data of mutation carriers, we did not identify additional genes which were only mutated in affected but not in the unaffected mutation carriers, or *vice versa*. Moreover, during our studies, we became aware of a monozygotic twin pair with a discordant phenotype, additionally arguing for somatic or environmental modifying factors. In >50 patients tested, we did not identify any additional somatic mutation in candidate genes. The HLA type, however, may add an additional susceptibility factor, as we observed that the haplotype HLA-DRB1 *15:01 and -DQB1 *06:02 in CTLA-4-insufficient patients might be associated with a severe immune dysregulation phenotype. Interestingly, the microbial composition in stool was different in affected CTLA4 mutation carriers when compared to unaffected mutation carriers. Details will be reported at the meeting. The infection history was not revealing, as for all infectious agents investigated, there were infected and not-infected mutation carriers in both groups. The analysis of the epigenetic signatures is pending and will be presented at the meeting.

**Conclusion:** According to our current assessment, the HLA-type and the microbiome are the most promising determinants of disease activity in patients with CTLA4-insufficiency.

**Disclosure of Interest**

None Declared

### P42 High prevalence of autoinflammatory diseases in autosomal dominant NFΚB1 insufficiency

#### B. Grimbacher, K. Thoma, on behalf of AG Grimbacher, M. Fliegauf

##### Institute for Immunodeficiency, Universital Hospital Freiburg, Freiburg, Germany

###### **Correspondence:** B. Grimbacher

**Introduction:** NF-κB1 (haplo)insufficiency due to heterozygous damaging variants in *NFKB1* has been recognized as an inborn error of immunity with immune dysregulation. *NFKB1* encodes the transcription factor precursor p105, which undergoes processing to produce the mature p50.

**Objectives:** Due to the highly heterogeneous immunological phenotypes, including incomplete penetrance (70%) and age-dependent disease severity, attempts to establish clear genotype-phenotype correlations have been unsuccessful so far. Therefore, we set out to better describe the phenotype of patients with variants in *NFKB1*. Moreover, functional evaluation of each identified *NFKB1* sequence variation was attempted to demonstrate causality.

**Methods:** We use a standard cell culture model, based on transient overexpression of mutant NF-κB1 proteins in HEK293T cells, to test the expression, subcellular localization, precursor processing, DNA binding and promoter activation. In addition, we test protein interactions, cytoplasmic retention/nuclear translocation and DNA-binding competition, following experimentally simulated pathway activation.

**Results:** Autoinflammatory symptoms in NFKB1 insufficiency include oral and genital aphthous ulcerations (18.5%), non-infectious episodes of fever and systemic inflammation (12%) as well as vasculitis (4.6%). Multi-organ autoinflammatory diseases (29.6%), including Behçet's disease (5.6%), are among most frequent clinical complications in affected mutation carriers besides hypogammaglobulinemia (88.9%), respiratory infections (83%) and reduced switched memory B cells (60.3%). The clinical spectrum further comprises autoimmunity (57.4%), lymphoproliferation (52.4%), non-infectious enteropathy (23.1%), opportunistic (15.7%) and gastrointestinal (28.6%) infections, and malignancy (16.8%).

Data regarding the prevalence of autoinflammatory complications in patients with mutations in *NFKB1* are currently being updated and will be presented at the meeting. The disease-causing mechanism presumably originates from imbalanced ratios of NF-κB signaling components, and altered NF-κB signaling dynamics.

**Conclusion:** Due to the high frequency of autoinflammation associated with NF-κB1 insufficiency, mutational and functional analyses of *NFKB1* mutations should be considered, particularly when autosomal-dominant autoinflammatory diseases are evaluated.

**Disclosure of Interest**

None Declared

### P43 Unraveling the mysteries of an unusual monogenic autoinflammatory disease presenting as an idiopathic inflammatory myopathy

#### J. Álvarez Troncoso

##### Internal Medicine, Hospital Universitario La Paz, Madrid, Spain

**Introduction:** We present the case of a 49-year-old woman from the United States of America with a previous diagnosis of urticaria-vasculitis since adolescence with the need for high-dose steroids and episodes of unclassified arthritis.

**Objectives:** To describe an atypical case of a 49-year-old woman diagnosed with an idiopathic inflammatory myopathy with positivity for anti-TIF1 antibodies that was actually a monogenic autoinflammatory disease.

**Results:** In 2015, she was diagnosed with adenocarcinoma of the cervix treated with radical hysterectomy and right adnexectomy without the need for chemotherapy or radiotherapy.

In the subsequent follow-up she presented absence of recurrence on PET-CT (at 1, 2 and 5 years). However, due to the persistence of arthritis, an immunological study was performed with only weak positivity for anti-TIF1 antibodies. On EMG she had mild myopathic changes. Muscle biopsy was anodyne (without HLA-I or inflammatory infiltrates), muscle MRI and the rest of the study was normal, including repeatedly normal CPK. However, in the presence of arthritis and skin lesions of urticarial vasculitis, treatment with methotrexate (MTX) and steroids was started with incomplete clinical improvement.

In July 2020, she attended first time to our consultation for the follow-up of myopathy due to anti-TIF1. The history was notable for the appearance of cold-induced urticaria / bruising, low grade fever and worsening of the arthritis during the last years. This ‘’cold intolerance” forced her to move to another city. She also mentioned that her teenage daughter had started 2 years ago with unstudied cold-induced urticaria. Given these data, a skin biopsy of urticaria and a study of systemic autoinflammatory diseases (SAID) directed at NLRP3 (but including the complete usual panel of SAID) was performed. The skin biopsy showed periecrine and perivascular neutrophils in the middle and deep dermis with no edema, compatible with CAPS.

Treatment with daily subcutaneous anakinra 100 mg was started with complete resolution of all skin lesions and arthritis, allowing the withdrawal of steroids and initiating a decrease in MTX. The genetic study of mother and daughter is pending at the time of case presentation.

**Conclusion:** CAPS or cryopirinopathies comprise three autosomal dominant conditions with different disease severity (FCAS, MWS, CINCA/NOMID). All CAPS are caused by mutations in the NLRP3 gene, which encodes NLRP3 protein or cryopyrin and lead to constitutive activation of NLRP3 inflammasome and IL-1β overproduction. CAPS skin biopsies are neutrophilic urticarial dermatosis with no or mild dermal edema and neutrophilic epitheliotropism (neutrophils around or within eccrine glands or ducts, or inside the epidermis). NLRP12-AD and NLRC4-AD are SAID with similar cutaneous and clinical characteristics but different cutaneous histopathology.

TIF1 is an E3 ubiquitin-ligase reported to be involved in DNA repair, dermatomyositis and cancer among others. Besides, there is increasing evidence that the expression of the NLRP3 inflammasome is dysregulated in many cancers. At the same time, some studies demonstrate that TIF1 might influence tumors via NLRP3 signaling. This could explain both clinical manifestations of our patient and TIF1 expression.

**Disclosure of Interest**

None Declared

### P44 Unilateral proptosis in a child with Behçet’s disease

#### F. G. Demirkan^1^, O. Akgun^1^, G. Kavrul Kayaalp^1^, F. Cakmak^1^, M. A. Kılıc^2^, E. P. Yıldız^2^, S. Sencer^3^, A. Unuvar^4^, N. Aktay Ayaz^1^

##### ^1^Pediatric Rheumatology; ^2^Pediatric Neurology; ^3^Neuroradiology; ^4^Pediatric Hematology, Istanbul University/Faculty of Medicine, Istanbul, Turkey

###### Correspondence: F. G. Demirkan

**Introduction:** Central nervous system (CNS) involvement is a rare complication of Behçet's disease (BD) in children and may present in the form of parenchymal changes or as damage to the vascular structures in its nonparenchymal form.

**Objectives:** A teenager having BD complicated with recurrent cerebral venous thrombosis is presented.

**Methods:** A-14 year-old boy followed up in dermatology and ophthalmology clinics for eight years with BD, was referred to the pediatric rheumatology clinic with complaints of headache, periorbital pain, diplopia, and blurred vision. His pathergy test was positive. He had a history of recurrent and unremitting oral ulcers. He was under daily colchicine therapy.

**Results:** On his physical examination, he was afebrile and ill-appearing. He had multiple painful aphthosis in his buccal mucosa, an averagely 1 cm in diameter, but no genital ulcer. Bilateral conjunctival bulbar hyperemia was present. He had also painful eye movements due to the bulging of the right eye anteriorly out of the orbita defined as proptosis. The rest of the neurologic and systematic examination was otherwise unremarkable. Ophthalmologic examination revealed bilateral optic disc swelling with established disc oedema and proptosis of the right eye.

The patient’s white blood count was 7.6 10^3^/μl with normal differential; red cell count was 10.9 g/dL; hematocrit, 31.9%, and platelets were 579 10^3^ /μl. C-reactive protein was 89.5 mg/L and D-dimer level was 640 μg/L.

CNS imaging by magnetic resonance imaging (MRI) and magnetic resonance venography (MRV) reported left transverse sinus thrombosis including left sigmoid sinus and internal jugular vein. Thrombophilia screening tests for identifying a hypercoagulable state showed no abnormality and no additional hereditary or acquired thrombosis risk factors were detected. He was treated with intravenous methylprednisolone pulse therapy (30 mg/m^2^/day) for 3 days. He was anti-coagulated with low molecular weight heparin. Acetazolamide and topiramate were initiated due to the optic disc oedema. At discharge, immunosuppressive therapy with azathioprine and anticoagulant therapy with enoxaparin was prescribed.

Control MRV after 2 months revealed significant regression in the thrombus. The patient had no complaints during the following 5 months and proptosis was recovered. D-dimer level was 200 μg/L. Acetazolamide and topiramate were stopped when ophthalmologic evaluation showed significant regression regarding disc oedema.

After 5 months, he presented with severe headache and tinnitus. On eye examination, optic disc swelling and oedema were progressed in the left eye. In MRV, a new thrombus image was reported at the same anatomical localization as 5 months ago which affected the left transverse sinus and sigmoid sinus. The D-dimer level was 190 μg/L and there was no significant abnormality in remaining laboratory examinations. Anticoagulant therapy was continued with enoxaparin. Methylprednisolone bolus was given and cyclophosphamide was initiated as immunosuppressive treatment.

Eventually, he is asymptomatic and has developed no new symptoms related to the disease. Eye examinations are performed periodically and there is no progression in fundus examination. His control cranial MRV revealed significant regression in the thrombosis. Ultimate D-dimer level is 190 μg/L. Monthly six doses of cyclophosphamide is planned in the treatment.

**Conclusion:** CNS involvement in children is a rarely reported manifestation of BD and this report has a distinct clinical view of a teenage boy presenting with proptosis and recurrent thrombosis.

**Key words:** Behçet's disease, cerebral *venous sinus thrombosis*, proptosis

**Disclosure of Interest**

None Declared

### P45 RIPK1 deficiency in a patient with severe arthritis

#### G. Kavrul Kayaalp^1^, F. Çakmak^1^, G. Yeşil Sayın^2^, A. D. Aslanger^2^, E. Yücel^3^, N. Aktay Ayaz^1^

##### ^1^Pediatric Rheumatology; ^2^Medical Genetics; ^3^Pediatric Immunology and Allergy, Istanbul University, Faculty of Medicine, İstanbul, Turkey

###### **Correspondence:** G. Kavrul Kayaalp

**Introduction:** Receptor Interacting Protein Kinase 1 (RIPK1) is a widely expressed cytosolic protein kinase found in protein complexes that mediate signal transduction from cell surface receptors. It has an essential role in controlling signaling pathways related to inflammation, apoptosis and necroptosis. RIPK1 deficiency causes impaired mitogen activated protein kinase signals and irregular cytokine production. Immunodeficiency, intestinal inflammation, and progressive polyarthritis have been reported in patients with *RIPK1* mutations, however the current knowledge about the clinical aspects of the disease is still scarce.

**Objectives:** Here we report a novel *RIPK1* mutation in a patient with severe arthritis and some distinct clinical features.

**Methods:** Clinical and laboratory features of the patient were described.

**Results:** Case presentation: An 18 months-old male has presented with recurrent febrile episodes and arthralgia in his multiple joints. His family reported febrile episodes in which he was diagnosed with respiratory tract infection (RTI) since three months of age. At the ages of 8 months and 16 months, febrile attacks were accompanied by severe arthralgia for 3 days with elevation of acute phase reactants. Family history revealed the diagnosis of familial Mediterranean fever (FMF) in his grandparent, uncles and cousins and rheumatoid arthritis in his great-uncle.

His physical examination was unremarkable. Blood count, acute phase reactants, liver and kidney function tests, urinalysis and serum immunoglobulin examinations were within normal limits for age. *MEFV* gene analysis was performed which revealed a heterozygous mutation in *V726A*. The patient was commenced on colchicine considering the diagnosis of FMF. After the colchicine treatment, his family reported a decrease in the number of febrile episodes, however at the age of two, joint swelling was accompanied to febrile attacks. Autoinflammatory genetics panel was negative. There were arthritis and limited range of motion in both knees, elbows and small joints of hands and feet. The patient was diagnosed with polyarticular juvenile idiopathic arthritis (JIA) due to persistent arthritis and intravenous pulse methylprednisolone treatment was given for 3 days which was switched to oral methylprednisolone and tapered. Methotrexate was initiated subcutaneously once a week. After the treatment, his arthralgia was subsided and a significant improvement was observed in his ability to walk, however joint swelling did not improve. The patient underwent to whole exome sequencing and a homozygous missense mutation in *RIPK1* at c.1849C>G [p.(His617Asp)] was detected, which was not previously reported in the literature and the databases. Whole body MR imaging revealed microatelectasis in both lungs, arthritis in bilateral glenohumoral and coxofemoral joints, edema and inflammation in adjacent soft tissues, and trochanteric bursitis. Control levels of serum immunoglobulin G, A and M were found to be low for his age. Low immunoglobulin levels and the history of frequent viral RTIs and microatelectasis in lungs were found consistent with humoral immunodeficiency, and intravenous immunoglobulin treatment was commenced once a month.

**Conclusion:** In this case, severe arthropathy was prominent with systemic inflammation. The absence of serious infections other than RTI, later onset of low immunoglobulin levels, and the absence of signs of intestinal inflammation were the distinct features of the patient. This case emphasizes that the diagnosis of RIPK1 deficiency should be considered in treatment-resistant arthropathies in childhood, especially in the presence of signs of inflammation, and the importance of genetic consultation in resistant cases.

**Disclosure of Interest**

None Declared

### P46 Complex genetic diagnosis of DADA2 in a patient with an ADA2 single allele mutation: the long and winding road

#### J. R. Marques Soares^1^, S. Buján Rivas^1^, R. Solans Laqué^1^, J. I. Aróstegui Gorospe^2^

##### ^1^Internal Medicine, Hospital Vall D´Hebron (Barcelona) SPAIN; ^2^Immunology, Hospital Clínic i Provincial - Idibaps, Barcelona, Spain

###### **Correspondence:** J. R. Marques Soares

**Introduction:** Deficiency of adenosine deaminase 2 (DADA2) (ORPHA: 404553) is a rare monogenic autoinflammatory disease with an autosomal recessive inheritance, deriving from biallelic *loss-of-function* mutations in the *ADA2* gene, which encodes for the homonym protein. Its heterogeneous clinical phenotype can range from multisystemic vascular inflammation, often mimicking polyarteritis nodosa (PAN), to hematological involvement (including cytopenias and bone marrow failure) and mild humoral immunodeficiency in the form of hypogammaglobulinemia and B cell lymphopenia. Because it was first described in 2014, prior cases were often diagnosed as PAN.

**Objectives:** To expand the knowledge about genetic basis of DADA2 with special attention to high suspicion cases with unconclusive genetic results, specially when the ADA2 activity test are unavailable.

**Methods:** Review of clinical charts, biochemistry and autoimmunity profiles and genetic tests of the proband and his 4 alive siblings pedigree. Genetic analysis of DNA samples obtained frompatients under informed consent, according to the Helsinki declaration.

**Results:** In the case hereby presented, after numerous tests, a first diagnosis of PAN-like disease was established in a 30-year-old male with no relevant familial history and a personal history of Raynaud’s phenomenon, livedo reticularis, digital ischemia, early-onset strokes, optic and peripheral neuropathy, cardiomyopathy, mesenteric and renal aneurysms, hypertension, vertigo, and multiple other systemic inflammation manifestations. Given the clinical history, atypical onset, the presence of hypogammaglobulinemia and disease progression despite several cyclophosphamide (CP) bolus and high-dose corticosteroid therapy, DADA2 diagnosis was considered.

At the age of 37 years a first genetic testing was performed through direct sequencing of *ADA2* via Sanger analysis using DNA extracted from peripheral blood. One of the most common pathogenic *ADA2* variants - p.G47R - was identified in a simple heterozygous genotype. These results led to hypothesize the presence of a structural gene variant in this locus that could justify the range of the previously mentioned clinical features. A whole genome sequencing (WGS) was executed without solid bioinformatic analysis results. Therefore, multiplex ligation-dependent probe amplification (MLPA) of the *ADA2* gene was performed, revealing a heterozygous genomic deletion that encompass exon 7 and flanking intronic boundaries. The Sanger exon 2 and exon 7 analyses of the 4 siblings identified an heterozygous p.G47R variant on proband's mother while the exon 7 avaluation concluded a trans inheritance pattern. The ADA2 activity was finally performed 11 years after the first diagnosis of PAN. As expected, the proband p.G47R variant / exon 7 deletion in trans manner produced an almost complete lack of activity. ADA2 activity for her mother, was reduced at 50%.

In the reported case, disease remission was achieved after initiation of anti-tumor necrosis factor (anti-TNF) therapy with Adalimumab.

**Conclusion:** DADA2 is a monogenic autoinflammatory disease with a wide clinical spectrum mimcicking PAN. Despite the lack of familar history, complex genetic changes can drive to a refractory PAN-like clinical picture. In this enviroment, the seric ADA2 activity is a useful screening tool with limited availability. Genetic diagnostic test with a more specific approach using WGS and/or MLPA can be the key for the final diagnosis.

**Acknowledgments**

Special acknowledge for dr. Solans, first physician who suspected the disease, and all the laboratory crews of the Immunology Department of Hospital Clínic i Provincial - IDIBAPS, and the Biochemistry Section of the Hospital Vall Hebron.

**Disclosure of Interest**

None Declared

### P47 Clinical course of aicardi-goutières syndrome in a girl with cholestatic liver disease and global neurological impairment

#### A. Diana^1^, R. Naddei^1^, A. Di Stazio^1^, F. Di Dato^1^, G. Cappuccio^1^, G. Terrone^1^, N. Brunetti-Pierri^1^, S. Volpi^2^, R. Iorio^1^, M. Alessio^1^

##### ^1^Department of Translational Medical Sciences, Section of Pediatrics, University of Naples Federico II, Naples; ^2^Center for Autoinflammatory Diseases and Immunodeficiency, IRCCS Istituto Giannina Gaslini, Genoa, Italy

###### **Correspondence:** R. Naddei

**Introduction:** Aicardi-Goutières Syndrome (AGS) is an autoinflammatory leukodystrophy characterized by global neurologic dysfunction, associated to an increased expression of type I interferon (IFN)-stimulated genes (the so-called IFN signature).

**Objectives:** To describe the clinical course of a 10-year-old girl diagnosed with AGS.

**Methods:** Case report.

**Results:** The girl was born at 37 weeks of gestation to non-consanguineous parents by urgent caesarean section due to fetal distress. Her mother suffered from congenital hearing loss and intellectual disability. Birth weight was 2150 g (small for gestational age). Since the first days of life, she presented hypotonia, thrombocytopenia (platelets [PLT]: 29400/mmc) and splenomegaly, so she was admitted to a Neonatal Intensive Care Unit (NICU) where she remained for four weeks. Suspecting neonatal sepsis, she underwent intravenous antibiotic treatment, soon after discontinued due to negative blood, urine and cerebrospinal fluid (CSF) cultures. TORCH showed positivity for rubella- and CMV-IgG, as well as maternal TORCH all over the pregnancy. Urine, plasma and CSF CMV-DNA was negative. Few days later, she presented fever and severe anemia requiring red blood cells transfusion in association with Staphylococcus Hominis sepsis, treated with intravenous antibiotics. At one-month follow-up, PLT were normalized and have remained normal since then. Echocardiography revealed anomalous pulmonary venous return, that required, at 8 months of life, coil embolization. Cerebral ultrasound showed lenticulostriate vasculopathy. During her stay in NICU, the patient also started a protein hydrolysate formula due to repeated episodes of vomiting. At the age of 4 years, cow’s milk proteins were reintroduced.

At the age of 4 months, she presented vomit and lethargy. She was admitted to a local hospital where anicteric cholestatic hepatitis (SGOT 1321 U/L, ALAT 1834 U/L, GGT 231 U/L), acidosis and hyperammonemia were found. She was treated by providing a glucose-based solution with pH and ammonium normalization and then she was transferred to our Department, due to persistence of transaminases and GGT elevation. Abdomen ultrasound was normal. IgG and IgM for CMV resulted positive, as well as urine CMV-DNA, therefore CMV-related hepatitis was suspected. Ursodeoxycholic acid (UDCA) was started. Transaminases persisted slightly elevated the following months, although CMV-IgM and urine CMV-DNA normalization. Eight months later, UDCA was discontinued but liver enzymes increased (SGOT 198 U/L ALAT 271 U/L), thus UDCA was resumed with progressive laboratory normalization. An extensive diagnostic work-up ruled out metabolic and autoimmune diseases. MRCP was normal. Array-CGH, karyotype and a NGS for genetic cholestatic liver diseases resulted negative. At the age of 8, UDCA was discontinued without any following alteration of liver function.

During early infancy, developmental delay was noted in association with spastic paraplegia and microcephaly. Brain MRI, performed at the age of 1 and 9, showed radiated crowns and semioval centers hyperintensity, lateral ventricles slight dilation and two symmetrical hyperintense areas at pontine tegmentum. She also presented IgA deficiency, mild psoriasis and dysmorphic elements (broad forehead, very high and external eyelids, long eyelashes, fetal pads, protruding auricles and supernumerary teeth).

At the age of 10, whole-exome sequencing showed the *de novo* variation c.2159G>A, p.(Arg720Gln) in the *IFIH1* gene, consistent with AGS. Therefore, blood IFN signature was investigated, resulting markedly elevated. She underwent a thorough autoimmune screening, which was unremarkable. Brain MRI was repeated, resulting stable compared to the previous ones, as well as neurologic evaluation.

**Conclusion:** AGS syndrome should be suspected in infants with idiopathic cholestatic liver disease, in particular when a history of unexplained neonatal thrombocytopenia is present, even before the neurological impairment onset. In this regard, IFN signature may be useful in the diagnostic work-up. Given the disease prognosis and the lack of approved therapeutic options, we are going to start an off-label treatment with a JAK/STAT inhibitor in this patient, although some issues are unsolved, such as the risk of liver toxicity associated to JAK/STAT inhibitors, the real effectiveness to treat a long-course brain disease and the difficulty in monitoring treatment efficacy. Written informed parental consent for publication was given.

**Disclosure of Interest**

None Declared

### P48 Myositis as an unusual presentation of ADA2 deficiency

#### M. Rossano^1^, F. Baldo^1^, S. Torreggiani^1^, L. Baselli^1^, S. Giliani^2^, S. Lanni^1^, A. Petaccia^1^, G. Filocamo^1^, F. Minoia^1^

##### ^1^Fondazione IRCCS Ca’ Granda, Ospedale Maggiore Policlinico, Milan; ^2^ASST Spedali Civili di Brescia , Brescia, Italy

###### **Correspondence:** M. Rossano

**Introduction:** ADA2 deficiency (DADA2) is a complex monogenic autoinflammatory condition characterized by protean manifestations, including haematological, cutaneous, articular and vascular involvement. Diagnosis is often difficult, due to the highly variable clinical phenotypes and the indolent course of the disease, often becoming overt when a major event occurs.

**Objectives:** To describe an unusual case of ADA2 deficiency presenting with myositis, due to compound heterozygosity for a missense mutation and a novel deletion in *ADA2.*

**Methods:** A 7-year old boy came to our attention for intermittent bilateral leg pain. Clinical evaluation was unremarkable, with normal strength. Blood tests revealed elevated inflammatory markers and normal lactic dehydrogenase (LDH) and creatinkinase (CK). Moderate lymphopenia was observed with low count of both CD19+ and NK cells, elevated CD3+HLA-DR+ cells, and low levels of IgM; double negative αβ T lymphocytes were normal (Table 1). Magnetic resonance (MRI) showed bilateral signs of polymyositis of the lower limbs. He subsequently developed nocturnal fever, multiple inguinal and axillary lymphadenopathy and splenomegaly. An infectious workup was negative, as well as an autoimmunity panel including myositis specific and associated autoantibodies, and serum markers of malignancies. PET-scan showed multiple tracer uptake in axillary, inguinal and abdominal lymph nodes (SUV max 2.2). A myogenic pattern of active denervation was confirmed by electromyography. Muscle biopsy revealed non-specific signs of inflammation with scarce macrophagic infiltration; no alteration of muscle fibres nor perifascicular atrophy or clear signs of vasculitis were reported. Bone marrow and lymph node biopsy with flow cytometry were negative for malignancy.

Due to the extremely painful musculoskeletal involvement associated to the development of recurrent infiltrative burning nodular skin lesions of both hands and feet, a symptomatic treatment with indomethacin was started with dramatic and prompt relief, but persistence of elevated acute phase reactants.

Genetic sequencing of ***ADA2*** revealed compound heterozygosity for a novel frameshift deletion in the catalytic domain, c.1100_1113del:p.I367Tfs*41, inherited from the father, and a maternally inherited missense variant, **c.1148G>A:p.G383D ,** previously described in compound heterozygosity in a patient with DADA2. ADA2 functional analysis confirmed pathological low activity of the enzyme. Brain angio-MRI was unremarkable. Etanercept treatment was started with rapid response of muscoloskeletal and skin involvement, normalization of inflammatory markers and regression of lymphoproliferation.

**Results:**

**Conclusion:** We report an unusual case of DADA2 presenting with myositis, which is seen in less than 1% of ADA2 deficiencies, due to a novel *ADA2* deletion leading to premature protein truncation, in compound heterozygosity with the recently described c.1148G>A mutation. The pathogenic role of the latter, so far reported in only two siblings in compound heterozygosity, is supported by our data. DADA2 phenotype spectrum is constantly increasing and description of peculiar cases could improve genotype/phenotype correlation and lead to significant progress in diagnosis, management and prognosis of these patients. Written informed parental consent for publication was given.

**Table 1 (abstract P48).** See text for description

Weeks from disease onset	4	6	14	16	21	43	46	56	61
Therapy			Indomethacin start			Etanercept start			
CRP (mg/dl)	2,64	3,19	5,13	2,08	0,9	2,53	0,05	0,05	0,05
ESR (mm/h)	52	63	52	40	35	44	13	4	2
Hb (g/dl)	11,1	9,9	9,9	9,4	10,2	9,5	10,3	11,3	12
WBC (cell/mm3)	5400	6100	7050	4990	5060	5540	4650	3260	4020
N (cell/mm3)	3760	4270	4760	2840	2910	3320	2210	1050	1890
L (cell/mm3)	850	750	1390	1380	1310	1470	1790	1570	1440

**Disclosure of Interest**

None Declared

### P49 A case of undifferentiated systemic autoinflammatory disorder

#### M. Tardi^1^, F. Annunziata ^2^, M. L. Pennacchio ^2^, F. Paciello^2^, B. Raffaele^1^, R. Sottile^1^, L. Martemucci^1^, F. Orlando^1^

##### ^1^Rheumatology Unit, Department of Pediatrics, Santobono-Pausilipon Children Hospital; ^2^Department of Translational Medical Sciences, Section of Pediatrics, University of Naples Federico II, Naples, Italy

###### **Correspondence:** M. Tardi

**Introduction:** Autoinflammatory diseases are a group of diseases related to dysfunction or dysregulation of innate immunity and can cause serious morbidity and mortality by affecting multiple organ systems. Patients with symptoms commonly found in autoinflammatory disorders may not fit a specific diagnosis, either because their clinical phenotype is not diagnostic or genetic tests are negative.

**Objectives:** To report an unsolved case of uSAID with successful anti- IL-1 treatment.

**Methods:** A twelve years old male with recurrent fever associated with fatigue, myalgia and oral aphthosis.

**Results:** Since the age of 11 years the patient had recurrent febrile episodes, with high fever (maximum temperature 40°C) of variable duration (range 3-10 days), monthly, associated with fatigue, myalgia, periorbital oedema and sometimes oral aphthosis. There was no rash and no history of genital aphthosis. The boy was born full term from physiological pregnancy and unrelated parents. Family history revealed mother with history of similar symptoms. At the admission, during a febrile episode, laboratory assessment showed low white blood cells count (3540/uL, n.v. 5000-19000), raised C reactive protein (CRP) (100,84 mg/l, n.v < 5), raised erythrocyte sedimentation rate (ESR)(101 mm/h, n.v. 0-13) and anemia (hemoglobin 8.9 gr/dl, n.v. 10-18). Covering the possibility of a bacterial infection, the patient was treated with broad spectrum antibiotics. Infections were excluded. Fecal calprotectin showed not pathological values. Chest x-ray, echocardiogram and electrocardiogram were normal. Abdominal ultrasound showed a liver and spleen increased volume. Bone marrow aspirate resulted negative. Because of the persistence of fever, he started on Indomethacin therapy with response. a whole body MRI STIR sequences was performed showing symmetrical signal hyperintensity of the cancellous bone of the distal femur, tibia and radius. On the basis of clinical history and imaging a chronic recurrent multifocal osteomyelitis (CRMO) was suspected. Because of the worsening of symptoms and laboratory parameters, even with maximum dosage of the drug, , after few months he switched to Colchicine therapy. He showed clinical and laboratory wellness associated with resolution of lesions to MRI study, but also in that case, after few months symptoms recurred and the initial dosage of drug was gradually increased, without benefits. Genetic tests for autoinflammatory diseases were performed, resulting negative. In the last hospitalization MRI STIR was repeated, confirming previous findings. He also underwent to bone biopsy, that excluded malignancy. In consideration of persistence of fever, associated with fatigue and myalgia and increased inflammatory markers (CRP 111,3 mg/L, ESR 49 mm/h), Anakinra (anti IL-1) was introduced (2 mg/kg/die), with ready significant clinical and laboratory response.

**Conclusion:** The spectrum of autoinflammatory diseases is rapidly expanding owing to recent developments in molecular sciences and genetics. However, for some autoinflammatory disorders no genetic alteration can be found. These cases are defined uSAID and Anakinra seems to be a feasible treatment option. Our patients presents features of different autoinflammatory disorders (recurrent fever, oral aphthosis, peri orbital oedema, symmetrical hyperintensity STIR lesions at MRI, leukopenia in acute phase, elevation of inflammatory indexes) but no mutations of associated genes were found. We expect to perform whole exome sequencing.

**Disclosure of Interest**

None Declared

### P50 Early-onset recurrent neuroinflammation and arthritis: a diagnostic and therapeutic challenge

#### S. Torreggiani^1^, M. Pozzato^1,2^, G. Filocamo^1^, S. Lanni^1^, S. Guez^1^, F. M. Triulzi^1^, A. Tozzo^3^, F. Andreetta^3^, P. Cavalcante^3^, M. Iascone^4^, C. Agostoni^1,2^, N. Bresolin^1,2^, S. Barbieri^1,2^, F. Martinelli Boneschi^1,2^, F. Minoia^1^, R. Dilena^1^

##### ^1^Fondazione IRCCS Ca’ Granda Ospedale Maggiore Policlinico; ^2^Università degli Studi di Milano; ^3^IRCCS Istituto Neurologico C. Besta, Milan; ^4^ASST Papa Giovanni XXIII, Bergamo, Italy

###### **Correspondence:** S. Torreggiani

**Introduction:** Neuroinflammation is increasingly identified in pediatric patients, as an isolated finding or in the context of autoimmune and autoinflammatory conditions. Due to marked clinical heterogeneity and phenotypic overlap, diagnosis is often challenging.

**Objectives:** To describe an unusual case of early-onset recurrent central nervous system (CNS) inflammation and demyelination associated with arthritis.

**Methods:** Whole exome sequencing (WES) was performed, followed by targeted reanalysis of neuroinflammation related genes.^1^

**Results:** A previously healthy 13-month-old girl was admitted to our hospital due to hyporeactivity and asymmetric tetraparesis with dystonic posturing, a week after an upper respiratory infection. Brain MRI showed symmetric talamic lesions, with involvement of the surrounding white matter and the right optic tract. CSF studies including PCR for HSV1-2, VZV, CMV and anti-MOG antibodies were normal. She was diagnosed with acute disseminated encephalomyelitis (ADEM) and treated with high dose intravenous methylprednisolone and 2 g/kg IVIG. Due to the age at onset and the basal ganglia involement**,** a Biotin-Thiamine-Responsive Basal Ganglia Disease was excluded by sequencing of the *SLC19A3* gene. Further metabolic investigations including mitochondrial DNA analysis were also negative. Three months later her neurologic exam was unremarkable with the exception of mild right lower limb dystonia; her brain MRI showed an almost complete resolution of previous abnormalities.

At 19 months of age, 17 days after the anti measles, mumps, and rubella vaccine, the patient presented with acute onset of strabismus, vertical nystagmus and ataxia. Brain and spinal cord MRI showed new lesions in the supra- and infratentorial white matter and in the optic nerves, and a longitudinally-extensive transverse myelitis (LTEM) lesion at C2-C4 level. As in the first episode, inflammatory markers were negative. CSF studies were normal, with oligoclonal bands, anti-aquaporin and anti-MOG antibodies negative both on serum and CSF. Complement function was normal and blood IFN signature was negative. A transient positivity for ANA (1:640) and for p-ANCA was observed but not confirmed in further testing. HLA-B51 was detected. WES was not contributive, despite a reanalysis of > 250 neuroinflammation related genes^1^. She again required high dose intravenous methylprednisolone and was started on periodic 2 g/kg IVIG for 12 months. Despite almost complete regression of neurologic signs, MRI lesions improved but persisted.

Nine months after the last neurological event, the patient developed arthritis involving the temporomandibular joint, the right knee and ankle, requiring treatment with intra-articular steroid injections. Based on the joint involvement and the positivity of HLA-B51, she was also started on methotrexate and colchicine. At the age of 38 months, she is neurologically stable with fluctuant right lower limb dystonia, despite incomplete regression of right ankle arthritis.

**Conclusion:** We described a case of early-onset recurrent neuroinflammation presenting with features of ADEM, MOG Associated Disease (MOGAD) and Neuromyelitis optica spectrum disorder (NMOSD), associated with arthritis. Clarifying the immunologic process underlying this phenotype will help guide treatment.

1. McCreary D, Omoyinmi E, Hong Y, et al. Development and Validation of a Targeted Next-Generation Sequencing Gene Panel for Children With Neuroinflammation. JAMA Netw Open. 2019;2(10):e1914274.

**Disclosure of Interest**

None Declared

### P51 chronic mucocutaneous lesions revealed underlying rare actinopathies-related disorders (Two case reports)

#### H. M. Abd El-Lateef

##### Pediatric Rheumatology Immunology department, Ain Shams University, Cairo, Egypt

**Introduction:** Actinopathies are a growing group of monogenic immune attributed diseases related to the defects in remodeling pathways of actin cytoskeleton.

**Objectives:** To confirm the significance of Immuno-Actinopathies role in the pathogensis of an expanding group of autoinflammatory diseases and primary immunodeficiencies.

**Methods:** Two patients referred with a history of chronic mucocutaneous lesions and selected laboratory immune phenotype abnormalities.

First, A 3 years old female patient of 1^st^ cousin consanginous parents presented with spontaneous vascuilitic cutaneous ulcerations in the inguinal flexure& the thighs (Biopsy confirmed) and multiple vulval abscesses.She had a serial history starting at the age of 2 months with bleeding per rectum & eczema diagnosed as cow milk protein intolerance, an althrough sequences of recurrent infections (bilateral purulent otitis media(P.aeruginosa) , skin abscesses &Staphylococcal pneumonia) and multiple confirmed food allergies during weaning trials. Second, A 5 years old male patient of 1^st^ cousin consanginous parents,presented with multiple spontaneous cutaneous sterile ulcerations,punched out lesions, necrotic demal lesions & paperous shiny skin in both upper& lower limbs together with acquired mild microstomia (a result of recurrent attacks of stomatitis), fissured lips, distorted tongue mucosal lining and decayed teeth. He had a chronic history of attacks of periodic fever .

**Results:** On physical examination, first patient was below 3^rd^ centiles for weight & height, coarse facies& no organomegaly or lymphadenopathy.Significant lab studies was in the form of chronic fluctuating thrombocytopenia(3- 36 X 10³μL) with small platelet volume(<7fl), high IgA (679mg/dl)&significantly high IgE(3600-7500 IU/L).Patient responded markedly to repeated sessions of pulse steroid theray (Methylprednisolone; 30mg/kg x 5 days) paired with IVIG followed by oral steroids and was referred for HSCT(Hematopoietic stem cell transplantation).Genetic studies revealed ARPC1B deficiency.

On physical examination, second patient was on 25^th^ centiles for weight&height, IQ 75, facies(frontal bossing, wide nostrils& long dense eye lashes) and no organomegaly or lymphadenopathy. Significant lab studies was in the form of high inflammatory markers (Neutrophilia,high ESR&CRP), low seum IgG(440 mg/dl) and high seum IgA(270 mg/dl).Patient responded partially to pulse steroid theray(Methylprednisolone; 30mg/kg x 5 days) and IVIG. Marked improvement was noted post an add–on Subcutaneous weekly Methotrexate injections with oral steroids. Genetic studies revealed a Homozygous WDR1mutation.

**Conclusion:** Refractory mucodermal disintegrity in pediatrics can clue to serious rare immunorelated disorders.Actinopathies paves the complexity of merging autoinflammatory states and immunodeficiencies.HSCT is a fundemental curative therapeutic line. Written informed parental consent for publication was given.

**Disclosure of Interest**

None Declared

### P52 A novel presentation of a hereditary periodic syndrome: which variant is responsible?

#### S. Al-Mayouf, L. Akbar

##### Pediatric Rheumatology- King Faisal Specialist Hospital and Research Center, Riyadh, Saudi Arabia

###### **Correspondence:** L. Akbar

**Introduction:** Recently, plasminogen, its activators, and its receptors have gained more attention in inflammation regulatory processes, including release proinflammatory signaling molecules, and thus its role has implications for a wide spectrum of clinical manifestations.

**Objectives:** Heres’ we presented a case of homozygous plasminogen variant managed initially as periodic fever syndrome.

**Methods: Case report**

A 9-year-old boy presented at the age of 18-months with periodic fevers and vomiting every 3 weeks, lasting for 48-72 hours. Heterozygous variant of *MEFV* gene was detected (c.442G>C; p.E148Q). Over a period of 7 years he developed recurrent headaches, abdominal pain, dysphagia, failure to thrive, eosinophilic esophagitis, recurrent otitis media, pneumonias, bronchial asthma like exacerbations, eye redness, tearing, delayed wound healing, periodontitis, and loss of teeth. Also, he was found to have Pseudotumor cerebri. Immunological and infectious work-up were inconclusive. Colchicine and short courses of steroids were initiated. However, anakinra was added with a partial response. It is worth mentioning that his parents are first-degree cousins; he has a twin sister with severe cyclic GI manifestations, with remarkable response to anakinra. Other siblings had sickle cell disease suffered from similar complaints as the index case with varying degrees in severity. With the constellation of findings and the inadequate response to therapies, whole-exome sequencing was obtained revealing homozygous variant in plasminogen gene; coding for plasminogen. However, his twin was found to have heterozygous plasminogen variant.

**Conclusion: **Our case had a constellation of findings cannot fully be explained by one classified autoinflammatory disease. Although we have no definite diagnosis; we believe that plasminogen gene variant, with the coexistence of *MEFV* gene variant might contribute to the clinical manifestations. Further studies needed to confirm this finding and allow more definitive conclusion. Written informed parental consent for publication was given.

**Disclosure of Interest**

None Declared

### P53 Identification and characterization of vexas syndrome caused by somatic mutations in UBA1

#### D. Beck, M. Ferrada, K. Sikora, A. Ombrello, D. Ospina Cardona, L. Wilson, P. Hoffman, K. Manthiram, I. Aksentijevich, P. Grayson, D. Kastner

##### National Institutes of Health, Bethesda, United States

###### **Correspondence:** D. Beck

**Introduction:** Identifying the causes of adult-onset rheumatic diseases remains a challenge, and limits diagnosis, prognosis, and targeted treatment. We hypothesized that mutations in genes regulating the post-translational modification ubiquitin, previously implicated in autoinflammatory diseases, may define new rheumatic disorders.

**Objectives:** This study aimed to identify novel genetic causes of autoinflammation using a "genotype" first approach.

**Methods:** We analyzed peripheral blood exome sequence data from 2,560 individuals with inflammation-related diagnoses for deleterious mutations in >800 ubiquitin-related genes. Sanger sequencing, digital droplet PCR, immunoblotting, immunohistochemistry, flow cytometry, and transcriptome/cytokine profiling were performed. CRISPR/Cas9 knockout zebrafish provided an *in vivo* model to assess *UBA1* gene function.

**Results:** Sixty adult males with autoinflammation and hematologic disease were identified with somatic mutations in *UBA1*, an X-linked gene, encoding the major E1 enzyme that initiates cytoplasmic ubiquitylation. Mutations were primarily identified at methionine 41, a highly conserved residue, and these mutations were not observed in exome sequences from over 80,000 healthy controls. Among affected individuals, mutations were found in more than half of hematopoietic stem cells, exclusively in peripheral blood myeloid cells, and not in lymphocytes or other non-hematopoeitic tissues. Patients developed an often-fatal, treatment-refractory inflammatory syndrome in late adulthood, with fevers, cytopenias, characteristic vacuoles in myeloid and erythroid precursors cells, dysplastic bone marrow, neutrophilic cutaneous and pulmonary inflammation, chondritis, and vasculitis. Patients fulfilled clinical criteria for inflammatory (relapsing polychondritis, Sweet syndrome, polyarteritis nodosa, giant cell arteritis) and hematologic (myelodysplastic syndrome or multiple myeloma) conditions. We have named this disease VEXAS (Vacuoles, E1 ubiquitin activating enzyme, X-linked, Autoinflammatory, Somatic) syndrome. Mutations at p.Met41 resulted in loss of the cytoplasmic isoform of UBA1 and decreased ubiquitylation and, unexpectedly, the expression of a novel, catalytically inactive, toxic isoform, in mutant, but not wildtype, lineages. We have further identified a genotype-phenotype correlation, suggesting specific risk factors for severe disease progression. Mutant peripheral blood cells, and depletion of *UBA1 in vitro,* exhibited activated innate immune pathways and evidence for unfolded protein response (UPR). Knockout of the zebrafish UBA1 cytoplasmic isoform homologue caused systemic inflammation.

**Conclusion:** By querying exomes for mutations in ubiquitylation genes, we have defined a novel disorder, VEXAS) syndrome, which connects seemingly unrelated adult-onset inflammatory syndromes and hematologic diseases and establishes a precedent for a new molecular taxonomy of rheumatic diseases. Our work also reveals somatic mutations as an underrecognized cause of adult-onset rheumatic diseases.

**Disclosure of Interest**

None Declared

### P54 Sharpenia, a novel autoinflammatory disorder caused by dysregulation in TNF-mediated cell death

#### H. Oda^1^, K. Manthiram^1^, H. Kuehn^1^, A. Rao^2^, D. Beck^1^, M. Nehrebecky^1^, A. Ombrello^1^, T. Romeo^1^, J. J. Chae^1^, I. Aksentijevich^1^, D. Kastner^1^

##### ^1^NIH, Bethesda, United States; ^2^Manipal Hospital, Bangalore, India

###### **Correspondence:** H. Oda

**Introduction:** The linear ubiquitin chain assembly complex (LUBAC) consists of HOIP, HOIL1, and SHARPIN and is essential for linear ubiquitination of NF-κB and other immune pathways. Patients with HOIP and HOIL1 deficiencies present with severe immunodeficiency, autoinflammation, and excessive glycogen storage. The role of cell death pathways in LUBAC deficiencies has not been investigated. *Sharpin*-deficient mice exhibit severe TNF-dependent skin inflammation due to enhanced apoptosis and necroptosis in the keratinocytes; however the role of SHARPIN in human disease is unknown.

**Objectives:** We aimed to investigate a 14 year-old boy from a consanguineous family in India who presented with childhood-onset polyarthritis, parotitis, colitis and hepatic glycogen deposition. No skin inflammation or immunodeficiency was noted.

**Methods:** We utilized whole exome sequencing in the proband and his unaffected parents to identify a genetic cause of the disease.

**Results:** We identified a homozygous frameshift mutation c.220dupC in *SHARPIN* in the proband. Patient cells revealed no detectable SHARPIN protein and markedly reduced expression of HOIP and HOIL1, suggesting destabilization of the entire LUBAC. Besides, mutant cells displayed a reduced activity of the canonical NF-κB pathway. We then study the role of apoptosis in the Sharpenia proband and other patients with LUBAC deficiency. We observed dysregulated apoptosis ex vivo, in vitro, and in vivo in all three LUBAC-deficient samples. Of note, the extent of apoptosis correlated with the severity of the disease. We identified a stark difference of inflammatory signatures between blood and synovial fluid samples in the Sharpenia patient, suggesting tissue-specific inflammation as the cause of this disease.Anti-TNF therapy achieved the complete resolution of inflammation, which further corroborates the role of TNF-mediated cell death in the pathogenesis of autoinflammation. Intriguingly, despite the absence of clinical immunodeficiency, the adenoids of the SHARPIN deficiency patient showed a remarkably diminished germinal center formation, which was also notable in HOIP and HOIL1 deficiency patients.

**Conclusion:** We identified the first case of human SHARPIN deficiency in a patient with autoinflammation and subclinical immunodeficiency. We re-define human LUBAC deficiency as an autoinflammatory disease caused by dysregulation in cell death pathways.

**Disclosure of Interest**

None Declared

### P55 New monogenic autoinflammatory disease due to homozygous loss of function mutation in tank binding kinase 1

#### P. Pimpale Chavan^1^, I. Aksentijevich^1^, D. Bogunovic^2^, J. Taft^2^, S. Khubchandani^3^, R. Khubchandani^4^

##### ^1^NHGRI, National Institutes of Health, Bethesda; ^2^Icahn School of Medicine at Mount Sinai, New York, United States; ^3^Section of Histopathology; ^4^Section of Pediatric Rheumatology, NH SRCC Children's Hospital, Mumbai, India

###### **Correspondence:** P. Pimpale Chavan

**Introduction:** TANK binding kinase 1 (TBK1) is a protein kinase with an important role in innate immunity by regulating IFN-I and NF-κB signaling, and TNF-induced RIPK1-dependent cell death. To date, only 3 cases (one sibship and the index patient) have been identified to have disease-causing variants in the *TBK1* gene.

**Objectives:** We describe the first case report of an Indian patient of Hindu ancestry with a homozygous predicted deleterious missense mutation in the *TBK1* gene.

**Methods:** A 8 year old male born of third degree consanguineous marriage was first seen at 4 years 7 months of age with a history of onset at 18 months. He presented with irregularly spaced episodic large and small joint arthritis lasting for few hours to 2-3 days, polymorphic skin rash described by his previous physician notes as ulcerative, nodular, urticarial, and one episode of seizures and status epilepticus.

**Results:** His weight (11.5kg) and height (93cm) were below 5^th^ centile for age, and he was developmentally normal. There was no lymphadenopathy or organomegaly and he had a 2 cms café au lait spot on the posterior aspect of the left knee. Laboratory investigations revealed hemoglobin of 7.9 -11.8 g/dL, white blood cells 8600-23700/mm^3^, platelets 3,00,000-7,19,000/mm^3^, ESR 7-90mm/Hr, CRP 13-69mg/dL, and Immunoglobulin profile were normal for age and gender. Anemia, neutrophilic leukocytosis, thrombocytosis, and raised acute phase reactants normalized between the episodes. Bone scan, MRI, and CT brain were normal.

Suspecting a systemic autoinflammatory disease (SAID), a next-generation sequencing SAID gene panel (53 genes) was utilized but was not contributory. Meanwhile, he was started on colchicine which had to be discontinued after the family reported an increase in the frequency of rash. His episodes responded well to p.r.n paracetamol or naproxen and the family was reluctant to add treatment without a definitive diagnosis. At age 7 years 9 months, he developed periorbital swelling, anasarca, and nephrotic range proteinuria and was started on prednisolone @2mg/kg by a nephrologist with a diagnosis of nephrotic syndrome. A kidney biopsy was performed when he was on steroid treatment, which revealed minimally involved glomeruli with no signs of chronicity or amyloid deposits and immunofluorescence was negative while electron microscopy revealed flattening of the foot processes. The proteinuria responded well to steroids. Whole exome sequencing was performed in the proband and his unaffected parent and identified a homozygous missense mutation in the kinase domain of the *TBK1* gene, c.634T>G (p.Y212D). Both the parents and elder female sib are healthy carriers for this variant. This missense substitution is classified as Likely Pathogenic by ACMG classification criteria with a CADD score of 24.9. Preliminary functional experiments support the pathogenicity of this variant.

The patient has been started on Infliximab biosimilar molecule since 3 months to which he has responded well with the decrease in episodes and duration /intensity of the pain of arthritis, no further skin rash and steroids have been tapered to low dose.

**Conclusion:** We report a new autoinflammatory disease that might be caused by a hypomorphic pathogenic variant in *TBK1*. Further studies are necessary to confirm the clinical significance of this finding. Written informed parental consent for publication was given.

**Disclosure of Interest**

None Declared

### P56 Nailfold capillaroscopy: a sensitive method for evaluating microvascular involvement in children with SARS-COV-2 infection

#### F. Çakmak^1^, A. Demirbuga^2^, D. Demirkol^3^, S. Gümüş^4^, S. Hançerli Torun^2^, G. Kavrul Kayaalp^1^, R. Eker Ömeroğlu^1^, A. Somer^2^, M. Uysalol^4^, R. Yıldız^4^, N. Aktay Ayaz^1^

##### ^1^Pediatric Rheumatology; ^2^Pediatric Infectious Diseases; ^3^Pediatric Intensive Care Unit; ^4^Pediatric Emergency Unit, Istanbul Faculty of Medicine, istanbul, Turkey

###### **Correspondence:** F. Çakmak

**Introduction:** The coronavirus (SARS-CoV-2) pandemic, known as COVID-19 has spread all over the world in a short period of time and caused the death of more than 2 million people to date. Although in severe cases, it mainly progresses as a serious lung disease such as pneumonia or acute respiratory distress syndrome (ARDS), numerous extrapulmonary manifestations due to systemic hyperinflammation associated with COVID-19 have been described. The hyperinflammatory state and the viral invasion may result in endothelial dysfunction and capillaroscopic examination of the nailfold may be a feasible method for monitoring the microvascular circulation in SARS-CoV-2 infection.

**Objectives:** With this study, we aimed to investigate the microvascular circulation in patients diagnosed with COVID-19 and multisystem inflammatory syndrome in children (MIS-C) by nailfold videocapillaroscopy (NVC).

**Methods:** Thirty-one patients with SARS-CoV-2 infection, 26 of whom were diagnosed with COVID-19 and 6 with MIS-C, and 58 healthy peers were included in the study. All fingers except the thumbs were examined paying greater attention to the ring finger of the non-dominant hand for the presence of any abnormality bilaterally and two images from eight fingers were obtained from both the study and control groups. Sixteen images were examined for the morphology of capillaries, presence of pericapillary edema, microhemorrhage, avascular area, and neoangiogenesis. These parameters were assessed as present or absent, and the presence of signs in at least two fingers was recorded as capillary abnormality in both groups. Capillary length, capillary width, apical loop, arterial and venous width, and intercapillary distance were measured from three consecutive capillaries from the ring finger of the non-dominant hand.

**Results:** COVID-19 patients showed significantly more capillary ramification (p<0.001), capillary meandering (p=0.04), microhemorrhage (p<0.001), neoangiogenesis (p<0.001), capillary tortuosity (p=0.003). Capillary density (p=0.002) and capillary length (p=0.002) were significantly lower in the patient group while intercapillary distance (p=0.01) was significantly longer compared with healthy volunteers. Morphologically, patients with MIS-C had a higher frequency of capillary ramification and neoangiogenesis compared with COVID-19 patients (p=0.04). Patients with capillary abnormalities had significantly higher levels of C-reactive protein (CRP) and D-dimer (CRP; 16.4 *vs* 2.2, p=0.04 and D-dimer; 900 *vs* 340, p=0.04).

**Conclusion:** Children diagnosed with COVID-19 and MIS-C present with several microvascular abnormalities on NVC examination. MIS-C is an emergency phenomenon in which evidence suggests activation of ECs as the key determinant in the pathogenesis of the disease, and NVC may be a useful non-invasive, valid method for assessing the microcirculatory status of children with MIS-C. As a preliminary one, our study may take attention to the use of NVC for follow-up of patients with SARS-CoV-2 infection during clinical course and management.

Key Words: COVID-19, Nailfold Videocapillaroscopy, microcirculation, endotheliitis

**Disclosure of Interest**

None Declared

### P57 Early recognition of multisystem inflammatory syndrome temporally associated to COVID-19 in the emergency department: a single tertiary care centre experience

#### A. Mauro^1^, M. Maglione^1^, F. Orlando^2^, A. Giannattasio^1^, R. Margherita^1^, T. Gagliardo^1^, L. Zenzeri^1^, S. Lenta^1^, S. Muzzica^1^, C. D'Anna^1^, C. Di Lillo^1^, R. Mancusi^1^, V. Tipo^1^

##### ^1^Emergency Department; ^2^Pediatrics, Aorn Santobono-Pausilipon, Naples, Italy

###### **Correspondence:** A. Mauro

**Introduction:** Multisystem Inflammatory Syndrome in Children (MIS-C) is a new and life-threatening disease temporally associated to Covid-19.

**Objectives:** The aim of the study is to analyze the clinical, laboratoristic and instrumental features of patients with diagnosis of MIS-C at the onset in order to early recognize the disease.

**Methods:** We retrospectively reviewed clinical records of children admitted to our Emergency Department between April 2020 and March 2021, who were ultimately diagnosed with MIS-C associated with SARS-CoV2. Data collected included all clinical and laboratory parameters at presentation to the Emergency Department. We also recorded data regarding the duration of fever and hospitalization and the presence of abnormalities at chest X-ray, abdominal and cardiac ultrasound.

**Results:** Clinical and laboratory data of the twenty-seven children retrospectively enrolled, including symptoms at presentation to the Emergency Department, are summarized in Table 1. Median duration of fever was 4 days (range 1.5 – 7). With the exception of fever, abdominal pain and diarrhea were the most frequent complaints at presentation. No significant differences were found between laboratory parameters in children with or without abdominal pain, diarrhea, vomit, conjunctivitis or rash.

Heart ultrasound showed no abnormalities in 11 out of 27 children (41%). Findings in other children were mainly represented by mild pericardial effusion (29.6%) and mild mitral valve insufficiency (25.9%). Minor abnormalities in the interventricular septal dynamics were detected in 3 subjects (11.1%). Abdominal ultrasound was unremarkable in 5 out of 27 patients (18.5%). Most children (51.8%) had mild-to-moderate peritoneal effusion, which was often associated with ileal loops wall thickening (29.6%). The thickened segments were mostly located in proximity of the ileo-cecal valve or of the appendix. Mesenteric lymphadenitis was found in eleven children (40.7%).

No significant differences were found in clinical or laboratory parameters between children with abnormal heart or abdominal ultrasounds and those without pathologic findings at these exams. Chest X ray at presentation showed no significant abnormalities in most patients, and only the child who died one day after admission showed bilateral basal opacities.

**Conclusion:** The collected data allow to identify clinical and laboratoristic tic elements of patients admitted to Emergency care unit to provide early recognition of the MIS-C .The study included a modest sample size and for this reason the generalizability of results is limited. A national multicentre study is ongoing.

**Table 1 (abstract P57).** Clinical and laboratory data of the study population

Clinical data	(n = 27)	Clinical data	(n = 27)	Laboratory Data	n = 27	Laboratory Data	n = 27	Laboratory Data	n = 27
***Gender, n (%)***		***Symptoms at presentation, n (%)***		***SARS-CoV2 serologic status, n (%)***		Hemoglobin	12.2 g/dL (9.4 – 15.5)	ALT	24 U/L (8 – 115)
Male	14 (52)	Fever	26 (96.3)	IgG+ / IgM-	24 (88.9)	Platelet count	197 10^3^/μL (62 – 513)	LDH	540 U/L (317 – 1014)
Female	13 (48)	Abdominal pain	16 (59.2)	IgG+ / IgM+	2 (7.4)	CRP	112.3 mg/L (9.15 – 420.8)	Albumin	3.4 g/dL (2.1 – 4.5)
***Age, yrs***	7.1 (0.8-12.3)	Diarrhea	14 (51.8)	IgG- / IgM+	1 (3.7)	PCT	2.38 ng/mL (0.16 – 188)	Triglycerides	193mg/dL (70 - 850)
***History of SARS-CoV2*** ***infection/contact, n (%)***		Vomit	12 (44.4)	***Blood tests***		Fibrinogen	598 mg/dL (223 – 1109)	Creatinin	0.43 mg/dL (0.15 – 1.63)
Previous infection	5 (18.5)	Conjunctivitis	13 (48.1)	WBC count	11.74 10^3^/μL (4.16 – 28.72)	D-dimer	1016 ng/ml (8.8 - 14776)	CK	80 U/L (16 - 5930)
Close contact with SARS-CoV2+ subject	9 (33.3)	Skin rash	11 (40.7)	Neutrophil count	9.41 10^3^/μL (3.29 – 24.64)	Ferritin	635.8 μg/L (31.3 – 8115)	BNP	199.5 pg/mL (5 - 5000)
Both	8 (29.6)	Other	0 (0)	Lymphocyte count	1.29 10^3^/μL (0.36 – 15.93)	AST	31 U/L (13 – 245)	Troponin	25.2 ng/L (0.02 - 1523)

Abbreviations: WBC, white blood cells ; CRP, C-reactive protein; PCT, procalcitonin; AST, aspartate aminotransferase; ALT, alanine aminotransferase; LDH, lactate dehydrogenase; CK, creatin kinase; BNP, brain natriuretic peptide

**Disclosure of Interest**

None Declared

### P58 Multisystem inflammatory syndrome in children: clinical characteristics, diagnostic findings and therapeutic interventions at a tertiary care center in South of Italy

#### F. Orlando^1^, A. Giannattasio^2^, F. Paciello^3^, A. Mauro^2^, M. Tardi^4^, S. Muzzica^2^, C. D'Anna^2^, M. Rosa^2^, M. Maglione^2^, R. Sottile^4^, L. Martemucci^4^, V. Tipo^2^

##### ^1^Department of Pediatrics, AORN Santobono Pausilipon, Napoli; ^2^Pediatric Emergency Unit, AORN Santobono Pausilipon; ^3^Department of Translational Medical Sciences, Section of Pediatrics, University of Naples Federico II; ^4^Department of Pediatrics, AORN Santobono Pausilipon, Naples, Italy

###### **Correspondence:** F. Orlando

**Introduction:** Multisystem inflammatory syndrome in children (MIS-C), also known as paediatric inflammatory multisystem syndrome temporally associated with COVID-19 (PIMS-TS), is a condition characterised by persistent fever, elevation of inflammatory indexes and evidence of organs involvement or shock.

**Objectives:** To describe clinical characteristics, diagnostic findings and therapeutic interventions of monocentric cohort of MIS-C.

**Methods:** Diagnosis of MIS-C was done following CDC criteria. Patients were hospitalised at Santobono-Pausilipon Children’s Hospital in Naples, Italy, from November 2020 to March 2021.

**Results:** MIS-C was diagnosed in 29 patients, 14 males (48.3%). Mean age at diagnosis was 7,2 years old (range 4 months-12,9 years). Contact with SARS-CoV-2–positive patient emerged in 18/29 patients (62%) while 5/29 patients (17,2%) reported symptomatic COVID-19 in the weeks before. SARS-CoV-2 serologic assayrevealed IgG +/IgM- in 100% of the patients. No one presented concurrent conditions but obesity in 6/29 (20,7%). Mucocutaneous involvement was evidenced in 21/29 patients (72%), gastrointestinal symptoms 22/29 (75.9%), cardiac involvement in 27/29 (93,1%). The most frequent symptoms were fever (100%), conjunctivitis (65.5%), abdominal pain (62%), diarrhoea (48,2%), rash (44,9%), vomiting (31%) and cheilitis (31%). Laboratory findings are summarised in table 1. Troponin resulted elevated in 16/29 (55,1%), associated elevation of BNP was evidenced in 12/29 (62%). Electrocardiography showed alterations in 25/29 (86,2%) while echocardiography in 21/29 (72%). Concerning therapy, 27/29 (93%) patients underwent parenteral antibiotics at the admission. Intravenous immunoglobulin (IVIG) was performed in 25/29 (86,2%) of patients. Due to cardiac involvement 13/29 patients (44,8%) received bolus of steroids. 4/29 patients (13,8%) presented worsening of clinical and laboratoristic parameters during treatment with steroids, requiring Anakinra. One patient died due to cardiogenic shock at the admission.

**Conclusion:** Mucocutaneous, gastrointestinal and cardiac involvement are the most common manifestations in our cohort, as also reported in literature. Biologic treatment was necessary in minority of patients. MIS-C is a new emerging condition and represent a challenge to paediatricians due to the severity of presentation. More data are needed to better define incidence and prognosis of that condition.

**Disclosure of Interest**

None Declared

### P59 COVID-19 pandemic - related chilblains: clinical and immunological characterization of an Italian cohort

#### S. Signa^1,2^, F. Manunza^3^, C. Pastorino^3^, P. Bocca^1^, A. Corcione^1^, F. Penco^1^, A. Bertoni^1^, I. Airoldi^4^, G. Viglizzo^3^, F. Morandi^4^, S. Rosina^5^, S. Viola^5^, C. Occella^3^, M. Acquila^6^, M. Podestà^4^, A. Ravelli^5^, E. Castagnola^2^, M. Gattorno^1^, S. Volpi^1^

##### ^1^UOSD Centro Malattie Autoinfiammatorie e Immunodeficienze; ^2^UO Malattie Infettive; ^3^UO Dermatologia; ^4^Laboratorio Cellule Staminali post natali e Terapie Cellulari; ^5^UOC Clinica Pediatrica e Reumatologia; ^6^UO Laboratorio Analisi, IRCCS G. Gaslini, Genoa, Italy

###### **Correspondence:** S. Signa

**Introduction:** During COVID-19 pandemic, acute acral chilblain-like lesions (ACBLL), reminiscent of lupus pernio, were observed during both first and second COVID-19 peak among patients with highly suspected (but mostly unconfirmed) infection with SARS-CoV-2.The aetiology of this phenomenon has not been elucidated yet and pathogenetic mechanism remains unknown. Several studies have investigated cytokine and chemokine profile in patients with COVID-19 but an accurate characterization of ACBLL patients is lacking.

**Objectives:** We aimed to describe the clinical, laboratory and immunological features of children presenting with ACBLL referred to our Institute during the COVID-19 pandemic spread.

**Methods:** We prospectively collected data of children referred to our Institute from April 1^st^ 2020 to February 28^th^ 2021. We investigate the presence of SARS-CoV2 infection through RT-PCR from nasopharingeal swabs and three different serologic kit.. All patients underwent a laboratory work-up including coagulation, viral serology and autoantibodies panel. Finally, we analysed peripheral blood IFN signature, a panel of inflammatory biomarkers in serum/plasma by a flow cytometry bead array (CXCL10, CXCL9, IL-6, IL-1β,TNFα) and the presence of SARS-CoV2 T specific lymphocytes.

**Results:** We examined 36 children during the first peak, and 11 children during the second COVID-19 peak (F: 28; median age 12 y), at a median delay of 26 days after symptoms onset (2-73 days). Fifteen patients (31%) presented non-specific systemic symptoms preceding ACBLL onset. Nine patients (19%) reported a possible contagion from a close contact. All patients presented stereotypical features resembling classical chilblains with acral erythematous-edematous violaceous plaques and nodules localized on the toes (n=35, 74%), the fingers (n=5, 10%) or on both sites (n=7, 15%). SARS-CoV-2 RNA detection resulted negative except for 2 patients. Furthermore, ten patients observed during the first wave showed a recurrence during the second (F:6), which developed 1-4 weeks after the second COVID-19 peak; the clinical features were comparable to those of the previous episode. Five of them (50%) reported non-specific systemic symptoms before onset and/or close contact with SARS-CoV2 positive subject. Repeated SARS-CoV-2 specific IgG/IgA tests were negative for all patients except for three cases (two of them with positive swabs). Neither common virus serology nor coagulation studies revealed significative results. Two patients presented positive ANA and anti β2 glycoprotein, respectively. A positive IFN signature was detected in 12/ 33 patients (36%).Among the 35 patients tested, the cytokine array showed high levels of IP10 (n= 35, range 12.4-739 pg/ml, n.v. 0.0-0.2 pg/ml) and a mild increase of IL-6 (n=21, range 2.4-401 pg/ml, n.v. 0.5-2.2pg/ml), without alterations of CXCL9, IL-1β and TNFa. The detection of SARS-CoV2 specific lymphocytes showed the presence of SARS-CoV2 specific lymphocytes in 9/17 (52%) patients tested (validated with positive and negative controls), only one of them with a positive serological test.

**Conclusion:** Albeit the pathogenetic mechanism of ACBLL remains to be elucidated, our preliminary results showed a significant increase in serum IP10 levels, not frankly associated with a peripheral blood IFN signature, which is instead a characteristic of pernio-related chilblains. We also proved the presence of a T-specific memory against in 50% of the tested patients, despite the negativity of coltures and serological tests, strengthening the link between SARS-CoV2 infection and this peculiar clinical manifestation.

**Disclosure of Interest**

None Declared

### P60 Diagnostic utility of genetic testing in autoinflammatory diseases - current situation in Belgium

#### A. Le Goueff^1^, F. Vandergheynst^1^, G. Smits^2^

##### ^1^Internal Medicine Department; ^2^Genetic Laboratory, Hôpital Erasme, Brussels, Belgium

###### **Correspondence:** A. Le Goueff

**Introduction:** Autoinflammatory diseases (AID) are a group of rare monogenic illnesses, leading to uncontrolled activation of the innate immune system and presenting with recurrent flares of systemic and localized inflammation. Diagnosis is confirmed by the detection of a mutation in an AID-related gene and improvements in sequencing techniques have enabled the discovery of new entities.

**Objectives:** The aim of our study is to explore the impact of evolving genetic testing methods for AID in Belgium and to determine whether increasing gene panels generate a higher diagnostic rate.

**Methods:** Retrospective study of 2620 patients that underwent sequencing for a clinical suspicion of AID in Belgium, between January 2015 and December 2020. Sequencing was performed through a 10-gene panel between 2015 and 2017, a 25-gene panel between 2018 and 2020 and mendeliome technology with a 66- and a 502- *in silico* gene panel in 2020.

**Results:** Diagnostic rate increased along with the expansion of the gene panel with a diagnostic yield of 16% with 10 genes, 18% with 25 genes and 23% with 502 genes. Factors that raised the diagnostic rate were Mediterranean ethnicity, abdominal and cardiorespiratory symptoms and absence of mucocutaneous, musculoskeletal, ophthalmologic or ENT symptoms.

**Conclusion:** Our study is the first nationwide study for autoinflammatory genetic testing and the first use of mendeliome technology for AID diagnosis. Although we confirmed that the bigger the gene panel, the higher the diagnostic rate, this technology generated inevitably a higher financial and human cost although the majority of diagnoses remained amongst the four original hereditary recurrent fevers (HRFs).

**Acknowledgments**

We thank the whole team from the ULB Centre of Human Genetics for their help and their support and especially Mélanie Delaunoy.

We also thank the statisticians, Jazon Bouziotis and Mélina Houinsou Hans for their work.

**Disclosure of Interest**

None Declared

### P61 Characteristics of hla linked dress reactions to inhibitors of IL-1 AND IL-6 in still's disease

#### V. Saper^1^, S. Prahalad^2^, S. Canna^3^, M. Ombrello^4^, M. Kostik^5^, A. Ravelli^6^, E. Cassidy^7^, S. Bhattad^8^, O. Kasapcopur^9^, T. Hahn^10^, O. Phadke^11^, E. Isupova^5^, J. Tibaldi^6^, C. Stingl^12^, A. Casey^13^, H. Wobma^13^, T. Klouda^13^, M. Alvarez^14^, G. Espada^14^, K. Torok^15^, A. Robinson^15^, M. Corriea Marques^4^, J. Hollenbach^16^, M. Fernandez Vina^1^, L. Tian^1^, E. Mellins^1^ on behalf of the Drug Hypersensitivity consortium

##### ^1^Stanford University, Stanford; ^2^Emory University, Children's Healthcare of Atlanta , Atlanta; ^3^Children's Hospital of Philadelphia, Philadelphia; ^4^NIAMS, Bethesda, United States; ^5^Saint-Petersburg State Pediatric Medical University, Saint-Petersburg, Russian Federation; ^6^IRCCS Istituto Giannina Gaslini, Genova, Italy; ^7^Geisinger Medical Center, Danville, United States; ^8^Aster CMI Hospital, Bangalore , India; ^9^Istanbul University-Cerrahpasa, Istanbul, Turkey; ^10^Penn State Health Children's Hospital, Hershey; ^11^Emory University School of Medicine and Children’s Healthcare of Atlanta, Atlanta; ^12^Spectrum Health, Grand Rapids; ^13^Boston Children's Hospital, Boston, United States; ^14^Hospital de Niños Dr RIcardo Gutierrez, Buenos Aires, Argentina; ^15^UPMC Children's Hospital of Pittsburgh, Pittsburgh; ^16^University of San Francisco, San Francisco, United States

###### **Correspondence:** V. Saper

**Introduction:** A subset of Still's patients receiving treatment with IL-1 and IL-6 inhibitors developed features (e.g., eosinophilia, atypical rash) that raised the possibility of drug reaction with eosinophilia and systemic symptoms (DRESS).

**Objectives:** To identify features of DRESS that distinguish it from active disease in Still's patients.

**Methods:** We organized a multi-center (48 institutions, 11 countries), retrospective case/control study of Still's patients treated with anakinra, canakinumab, or tocilizumab (anti-IL-1/IL-6). Inclusion as a case required physician suspicion of a drug reaction during anti-IL-1/IL-6 treatment. Drug-exposed controls from the same sites (institutional controls) were randomly selected Still’s subjects not satisfying the case definition. Each subject was scored for DRESS using the validated scoring system, RegiSCAR for DRESS, which specifies that features may be asynchronous and discontinuous. Demographic and clinical features of cases and controls were compared; frequencies of some features were compared to population frequencies. HLA allelic frequencies were compared in 71 DRESS cases and 30 drug-tolerant controls.

**Results:** Still’s onset age was enriched for <2.5 years in cases vs institutional controls; no significant differences were found in frequency of onset age >10 years, adult (>16y) onset of Still's, sex, or MAS occurrence pre-treatment. Interestingly, ten cases reported serious comorbidities: four with severe congenital heart disease (CHD),~40X expected live birth rate for CHD, pre-term birth (3) resulting in bronchopulmonary dysplasia (BPD) in 2 (~30X expected live birth rate), congenital lung disease (1), severe neonatal pneumonia (1), and 2 with CHD and 1 with BPD were among four with Trisomy 21 (~50X expected live birth rate). Comorbid conditions were mostly (7/10) among those with Still’s onset age <2.5 years. Eosinophilia and atypical rash were markedly enriched in cases vs controls, as expected (*P* values 7.1 x10^-31^ and 2.5 x10^-28^, respectively). Unexpectedly, differences were also observed in frequencies of abdominal pain (*P*=9.6 x 10^-6^) and of transfusion in absence of macrophage activation syndrome (MAS) or bleeding (*P*= 1.9x10^-4^). MAS during treatment occurred almost exclusively in DRESS cases (*P*= 6.2 x 10^-16^). HLA-DRB1*15 alleles were strikingly associated with DRESS, but did not distinguish DRESS cases with versus without diffuse lung disease (DLD) developing during anti-IL-1/IL-6, MAS or comorbid conditions. Preliminary data on outcome after careful drug removal include improvement or resolution of lung disease in 11 cases stopping drug with ≥3mos of follow up.

**Conclusion:** A number of clinical features characterize serious DRESS to IL-1/IL-6 inhibitors in Still’s disease patients. These features are shared by subjects with (~80%) and without the HLA risk allele. For the former, HLA testing allows pre-prescription risk determination. DRESS to IL-1/IL-6 inhibitors in Still’s may be enriched in those with younger onset age, co-occurring cardiopulmonary disease, prematurity, or Trisomy 21. Patient outcome with drug removal, as indicated for DRESS, will be important to evaluate.

**Disclosure of Interest**

None Declared

**Table 1 (abstract P61).** See text for description

	DRESS cases	Institutional controls	*P* value	OR (95CI)
Still’s onset age < 2.5 years	43% (29/67)	19% (19/100)	8.9 x 10^-4^	3.2 (1.6-6.5)
Abdominal pain	24% (16/67)	2% (2/100)	9.6 x 10^-6^	15.4 (3.4-70)
Transfusion (no MAS or bleeding)	13% (9/67)	0% (0/100)	1.9 x 10^-4^	Infinite
Atypical non-evanescent rash	90% (60/67)	5% (5/100)	2.5 x 10^-28^	98.6 (34-286)
Absolute eosinophils >700/ul	79% (53/67)	0% (0/100)	7.1 x 10^-31^	infinite
	**DRESS cases**	**Drug-tolerant controls**		
HLA-DRB1*15:XX (all ancestries)	79% (56/71)	7% (2/30)	not applicable across ancestries	
HLA-DRB1*15:01 (White)	79% (38/48)	0% (0/19)	8.2 x 10^-10^	infinite
Anti-IL-1 exposure	96% (68/71)	86% (26/30)	NS (0.19)	
Tocilizumab exposure	27% (19/71)	40% (12/30)	NS (0.24)	
Elevated AST/ALT	75% (53/71)	13% (4/30)	1.1 x 10^-8^	19 (5.9-62)
	**DRESS** *without* **DLD**	**DRESS** *with* **DLD**		
HLA-DRB1*15:XX	68% (15/22)	84% (41/49)	not applicable across ancestries	
MAS during drug	59% (12/22)	71% (34/49)	NS (0.29)	

### P62 Actinopathies that can be mistaken as hyper ige autosomal recessive ARPC1B deficiency is an actinopathy, this pathology is similar way to Hyper IgE syndrome with recurrent infections, eczema, IgE elevation and vasculitis

#### N. Gomez Hernandez^1^, M. Ortega Cisneros^1^, M. A. Yamasaki Nakashimada^2^, A. Delgado Bañuelos ^1^, R. Benitez Serrano^1^ on behalf of Institute National Pediatric Mexico , City

##### ^1^Instituto Mexicano Del Seguro Social, Guadalajara; ^2^IInstituto Nacional Pediatria, Ciudad de Mexico, Mexico

###### **Correspondence:** N. Gomez Hernandez

**Introduction:** Actinopathies that can be mistaken as Hyper IgE

Introduction :

Autosomal recessive ARPC1B deficiency is an actinopathy. Defective actin polymerization affects hematopoietic cells, impairing their migration and immunological synapse , characterized by leukocytosis, eosinophilia, platelet abnormalities and hypergammaglobulinemia; and clinically, by eczema and food allergy ,infections caused by bacteria, fungi and viruses, vasculitis, and bleeding diathesis.

**Objectives:** Case Report

28-year-old male, originally from Michoacan Mexico, his parents are first cousins. The patient began a three-month-old developed an abscess with an anorectal fistula that required surgery at 10 months he was hospitalized for an infectious gastroenteritis and a few weeks later once again for a urinary tract infection. This patient presents eczema and recurrent abscesses from one year of age, multiple episodes of otitis media and sinusitis,.

At the age of 8 years he was admitted to the National Institute of Pediatrics of Mexico City with a one-month history of cough and fever, as well as two months of painful erythematous subcutaneous nodules on both legs. Physical examination revealed a keloid scar on the abdomen after Nissen fundoplication, a normal BCG scar, diffuse crackles throughout both lung fields, and nodular erythematous lesions on both legs. Laboratory tests showed Hb 9.8 g / dl, leukocytes 15,200 / μl, neutrophils 73%, lymphocytes 17%, eosinophils 5% , 317,000 / μl platelets. IgE 2000 IU / ml, IgG 1870 mg / dl , IgM 420 mg / dl and IgA 968 mg / dl . He also had a negative ANCA and low positive ANA, while tuberculin and yeast tests were negative. Sputum cultures and gastric aspirates for fungal and bacterial organisms were also negative. The CT scan of nasal and pulmonary sinuses revealed pansinusitis and saccular bronchiectasis located in the right lung. Skin Biopsy of the leg lesions showed septal and lobular paniculitis with predominant infiltration of lymphocytes and histiocytes.

Initially, a diagnosis of HIES with erythema nodosum was made: the patient's NIH scoring system was 39, with a positive result diagnosis for scores greater than 20

Treatment with amoxicillin, thalidomide, and prednisone was started with improvement of respiratory symptoms and resolution of skin lesions. At age 9 he had multiple flat warts involving the trunk. The skin biopsy revealed a human papillomavirus infection. The patient was treated with topical retinoids leading to a gradual resolution of the warts, and thalidomide and corticosteroids were discontinued. In January 2003 he developed thrombocytopenia (63,000 / μl). At age 10, a lung biopsy was performed due to persistent cough and wheezing despite taking inhaled steroids and prophylactic antibiotics: results revealed obliterative bronchiolitis organizing pneumonia. Mesenteric vasculitis was detected, initiating management with oral cyclosporine and continued with MMF 1 g / day and monthly intravenous gamma globulin at a replacement dose in the Institute Mexican Secure Social .

Immunosuppressive treatment was suspended because, at the age of 21 he developed a disseminated molluscum contagiosum on the trunk, which is why weekly subcutaneous application of interferon alpha is indicated with total resolution of his lesions. He has a sequencing study for variants STAT 3, ERBB21P, CARD 11, DOCL8 IL6ST, PGM3, SPINK 5 with negative result. It is finally documented ARPC1B.

**Conclusion:** ARPC1B deficiency phenotype the clinical defect appears to be characterized by recurrent bacterial and viral infections, extensive eczema, allergies, thrombocytopenia, and skin vasculitis

This phenotype is expressed in a similar way to Hyper IgE syndrome with recurrent infections, eczema, IgE elevation and vasculitis .This particular case made us think about the importance of having a genetic diagnosis. Written informed patient consent for publication was given.

**Disclosure of Interest**

None Declared

### P63 Allergic reactions of COVID-19 vaccine in a teaching hospital

#### P.-C. Chen^1^, K.-H. Yang^2^

##### ^1^Taipei City Hospital, Taipei, Taiwan, Province of China; ^2^Pharmacy, Taipei city hospital, Taipei, Taiwan, Province of China

###### **Correspondence:** P.-C. Chen

**Introduction:** Because of the epidemic, countries have begun to administer the COVID-19 vaccine. The adverse reactions (ADRs) following the vaccine can be divided into three categories. Categories are allergic reactions including anaphylaxis, vasovagal reaction including vasovagal syncope and vaccine side effects including local and systemic. Allergic reactions occurring within 5-30 minutes are considered as autoimmune problems, and the problems caused are usually unpredictable and may be serious.

**Objectives:** For further analysis of the types of allergic reactions, medical professionals could remind the patient, prepare the responder as soon as possible, and as a reference for diagnosis and related treatment.

**Methods:** This study is a cross-sectional study that analyzes the notification data of adverse drug reaction cases in a regional hospital from March 30 to July 31, 2021, including the AstraZeneca® (AZ) brand and the Moderna® brand. And analyze whether the discomfort immediately after vaccination is Allergic reactions, vasovagal reaction or vaccine side effects.

**Results:** The total number of COVID-19 vaccines administered under the AZ® brand was 27,131, and Moderna® had a total of 9,203 people. A total of 80 adverse events were received. The AZ ® brand had 75 and the Moderna® brand had 5 ADRs. Among the ADRs of AZ®, there are 17 allergic reactions (22.67%), 1 vasovagal reaction reaction, and 57 vaccine side effects. Among the ADRs of Moderna®, there are 3 allergic reactions (60.00%), 0 vasovagal reaction reactions, and 2 vaccine side effects.

Out of a total of 20 Allergic reactions, 20 cases require additional processing to deal with side effects. These side effects include problems related to the injection site (redness at the injection site*2, itching at the injection site*5, rash at the injection site*3, soreness at the injection site, pain at the injection site, swollen left upper arm*2, red upper left arm, lower arm Itchy rash on the other side of the armpit*2), skin-related problems (red rash on the chest with oxygen, hives*2, itchy rash on the face and neck*2, skin rash*4), other problems (wheezing, vomiting*3, red and swollen eyes, chest tightness, dizziness). Among them, one case was reported as a "serious adverse event", who died eight hours after vaccination.

**Conclusion:** In the ADR of AZ®, 22.67% of allergic reactions is allergic reactions, which is 0.63 ‰ of the number of people who were administered. Although the proportion is not high, the adverse reactions caused by autoimmunity are likely to become very serious. Medical professionals should pay more attention to these information before administering the vaccine.

**Acknowledgments**

Taipei City Hospital

**Disclosure of Interest**

None Declared

